# Molecular Characterization of Rickettsial Agents in Ticks (Acari: Ixodidae) from Sri Lanka

**DOI:** 10.4269/ajtmh.21-0995

**Published:** 2022-04-11

**Authors:** Gregory A. Dasch, Marina E. Eremeeva, Maria L. Zambrano, Ranjan Premaratna, S. A. M. Kularatne, R. P. V. Jayanthe Rajapakse

**Affiliations:** ^1^ Rickettsial Zoonoses Branch, Centers for Disease Control and Prevention, Atlanta, Georgia;; ^2^ Jiann-Ping Hsu College of Public Health, Georgia Southern University, Statesboro, Georgia;; ^3^ University of Kelaniya, Ragama, Sri Lanka;; ^4^ University of Peradeniya, Peradeniya, Sri Lanka

## Abstract

Because the majority of spotted fever group rickettsiae are transmitted to humans by tick bites, it is important to understand which ticks might play a role in transmission of rickettsial pathogens in Sri Lanka. The purpose of our study was to conduct molecular surveillance of 847 ticks collected in different locations in central Sri Lanka to determine which were infected with *Rickettsia* and Anaplasmataceae. Molecular methods were used to identify the ticks and the agents detected. Most ticks (*Amblyomma*,* Haemaphysalis*, and *Rhipicephalus*) were collected by flagging, and lower number was collected from dogs, cattle, pigs, a pangolin, and tortoises. Five spotted fever genotypes were identified: a *Rickettsia africae-*like agent in *Amblyomma* larvae, *Rhipicephalus massiliae* and a related genotype identified in association with the tropical type of *Rhipicephalus sanguineus* from dogs and *Rhipicephalus haemaphysaloides* from dogs and cattle, and *Candidatus R. kellyi* and another novel genotype (SL94) in *R. haemaphysaloides.* Twenty-three ticks were positive for Anaplasmataceae, including one *Anaplasma* and two *Ehrlichia* genotypes. Because the sequence database for both ticks and rickettsial agents from Sri Lanka and southern India is not extensive, additional molecular characterization of the tick species of Sri Lanka and their rickettsial agents is required to understand their pathogenic potential more completely. However, several of the agents we identified in this survey may well be pathogenic for humans and domestic animals, and should be considered as a part of epidemiological surveillance and patient management.

## INTRODUCTION

Sri Lanka is an island country in South Asia situated in the Indian Ocean south of India. The country’s tropical climate is influenced by the prevailing ocean winds. Most of the eastern, southeastern, and northern parts of Sri Lanka are more arid, whereas the central highlands and southwestern parts of the country are more humid and get more rainfall.^1^ Thus, a range of environmental habitats suitable for different animals are present. As in many tropical countries, mosquito-borne diseases were common in Sri Lanka for many decades. However, in 2012, Sri Lanka reported a zero incidence of autochthonous malaria and received WHO certification of its malaria-free status in 2016.^2^^,^^3^ This situation shifted the predominance of vector-borne and zoonotic diseases of concern within the country, resulting in additional attention to the diagnosis of dengue, leptospirosis, and rickettsioses.^2^

The recognition of scrub typhus, caused by *Orientia tsutsugamushi* and transmitted by *Leptotrombidium* mites, in Sri Lanka dates to World War II; it is known to occur in both the hill country and lowlands.^4^^,^^5^ More recently, the frequent occurrence of spotted fever rickettsioses has been demonstrated serologically in both ecozones in Sri Lanka, but particularly in the central hill country.^6^^–^^9^ Depending on the study setting and cohort of the participants, the incidence of spotted fever group (SFG) rickettsiosis-positive individuals ranged from 9.7% among 883 febrile patients in southern Sri Lanka to 63.8% among patients with compatible symptoms in the central province of the country.^7^^,^^10^ Because antigen from African strains of *Rickettsia conorii* is commonly used for serodiagnostic tests in Sri Lanka, it is sometimes interpreted incorrectly as exposure to *R. conorii*, as this is a group-specific test.^11^^–^^15^ However, testing of sera of pediatric patients against a panel of eight different SFG rickettsial antigens, and subsequent cross-adsorption and Western blot analysis suggested that exposure to different species of SFG *Rickettsia* occurs in Sri Lanka.^16^ The descriptions of Sri Lankan patients with SFG rickettsioses includes some severe clinical manifestations, including the development of acute vasculitis, arthritis, and fern leaf skin necrosis.^7^^,^^8^^,^^17^^,^^18^ To date, there are only two publications reporting polymerase chain reaction (PCR)-based diagnostic findings in febrile patients from Sri Lanka.^18^^,^^19^ The first article reports detection of the 17-kDa SFG specific protein antigen gene in the skin biopsies of patients with fern leaf necrosis^18^; however, the specific etiological agent is uncertain because of significant genus-level nucleotide sequence conservation of this gene among SFG rickettsiae.^20^^,^^21^ A second article described a returning traveler from the jungle of Sri Lanka who allegedly experienced a febrile illness with enlarged lymph nodes, a maculopapular rash, and an eschar; this patient tested PCR positive for *Rickettsia sibirica mongolotimonae*,^19^ although the sequence of the amplicon was not provided for analysis and comparison. Nevertheless, both reports provide direct molecular confirmation of SFG rickettsioses occurring in Sri Lanka.

People living in the hilly central region in Sri Lanka often sleep on the ground and commonly experience intra-aural tick infestation (otoacariasis), resulting in facial palsies.^22^ At least four different genera of Ixodid ticks, including *Dermacentor*,* Amblyomma*,* Rhipicephalus*, and *Hyalomma* species, were found in otoacariasis patients in Sri Lanka.^23^ These patients exhibited seroconversion to SFG rickettsiae and prompt recovery after doxycycline treatment.^22^ A country-wide surveillance effort identified as many as 12 Sri Lankan tick species that attach readily to people, and 19 tick species were found on peri-domestic animals.^24^ Furthermore, 21 tick species were collected from diverse wild animals, including five different *Amblyomma* species infesting reptiles.^24^^,^^25^ Domestic animals shared tick species typically found on wildlife, suggesting that natural habitat destruction and forest fragmentation may cause wild animals to enter urban and semi-urban neighborhoods, so close contact between wild and domesticated animals can occur. Finally, bovine anaplasmosis resulting from *Anaplasma marginale* is a serious threat to the cattle industry of Sri Lanka, as in many other tropical and subtropical countries.^26^ The occurrence of human and canine Anaplasmataceae infections has not been reported in Sri Lanka.

The purpose of our study was to conduct molecular surveillance on ticks collected in different locations in central Sri Lanka to determine which were infected with *Rickettsia* and Anaplasmataceae. Molecular methods were used to identify the ticks and the agents that were detected.

## MATERIALS AND METHODS

### Tick collection.

Ticks were collected from different domestic animals that are in close association with the homes and farms of people in Sri Lanka, and by flagging near domiciles upon receiving the owner’s consent. Collection at the Wasgamuwa National Park was done after receiving approval from the local wildlife manager. Additional samples were collected from the animals housed at or brought for examination to the University of Peradeniya Teaching Farm (near Kandy). All ticks were placed in vials containing 70% ethanol and were kept refrigerated. Ticks were examined individually and identified for genus, sex, and life stage using standard taxonomic keys.^1^^,^^24^^,^^27^

### DNA extraction.

Ticks were surface disinfected though sequential washes with 10% bleach, 70% ethanol, and sterile distilled water; air-dried; frozen in liquid nitrogen; and crushed using sterile Kontes pestles (Kimble-Kontes, Vineland, NJ). The powder was then resuspended in 200 μL nuclei lysis solution (Promega, Madison, WI) supplemented with ethylenediaminetetraacetic acid and proteinase K (QIAGEN, Valencia, CA), and incubated overnight at 56°C. DNA was extracted using a Wizard SV 96 Genomic DNA Purification System (Promega) and a Biomek 2000 Laboratory Automation Workstation (Biomek, Fullerton, CA), as described previously.^28^ Each DNA was eluted with 100 μL of distilled water and stored at 4°C before testing. Adults and nymphs were processed individually, and larvae were processed in pools (2–11 larvae per pool, depending on their origin and collection site), resulting in a total of 304 DNA samples for further testing.

### Molecular identification of ticks.

The ticks were identified primarily by amplification and sequencing a fragment of their 12S ribosomal RNA (rRNA) mitochondrial gene using T1B and T2A primers (Table 1) according to a previously described protocol.^29^^,^^30^ Fragments of COII gene (ticks SL94, SL141, SL148, SL154, SL193, SL199, and SL203) and internal transcribed spacer 2 (ITS2) (SL91, SL94, SL141, SL193, and SL199) were also amplified and sequenced for selected ticks.^31^ Primer sequences and associated information for individual gene fragments are listed in Table 1.

**Table 1:** Primers used in this study

Target organism	Target gene	Application	Primer name	Sequence 5'-3'	Reference
Tick	Mitochondrial 12S ribosomal RNA gene	Speciation of ticks (end point PCR)	T1B T2A	AAACTAGGATTAGATACCCT AATAGCGACG GGCGATGT	Beati and Keirans^29^
	COII gene		COIIF COIIR	TCAGAACAYWCYTTYAATCAAAAT CCACAAATTTCTGAACATTGWCCA	Beati et al.^31^
	ITS2		F2LITS2 McLR	TGAGGGTCGGATCAYATATCA GTGAATTCTATGCTTAAATTCAGGGGGT	Beati et al.^31^
SFG *Rickettsia*	*omp*A	Testing for SFG rickettsiae (SYBR - Green PCR)	Rr190-547 Rr190-701	CCTGCCGATAATTATACAGGTTTA GTTCCGTTAATGGCAGCATCT	Eremeeva et al.^32^
	*omp*A	Speciation of SFG rickettsiae (end point PCR)	Rr190-70 Rr190-701 Rr190-602	ATGGCGAATATTCTCCAAAA GTTCCGTTAATGGCAGCATCT AGTGCAGCATTCGCTCCCCCT	Eremeeva et al.^33^
	*omp*B		120-M59 120-807	CCGCAGGGTTGGTAACTGC CCTTTTAGATTACCGCCTAA	Roux and Raoult^72^
	*sca*4		D1f D928R	ATGAGTAAAGACGGTAACCT AAGCTATTGCGTCATCTCCG	Sekeyova et al.^73^
	*glt*A		RpCS877F RpCS1258R	GGGGACCTGCTCACGGCGG ATTGCAAAAAGTACAGTGAACA	Eremeeva et al.^33^
Anaplasmataceae	16S rRNA gene	Testing for *Ehrlichia* and *Anaplasma* (SYBR-Green PCR)	SYBR-F SYBR-R	AACACATGCAAGTCGAACGG CCCCCGCAGGGATTATACA	Eremeeva et al.,^34^ Li et al.^35^
	*gro*EL	Speciation of *Ehrlichia* and *Anaplasma* (nested PCR)	GRO607F GRO1294R GRO677F GRO1121R	GAAGATGCWGTWGGWTGTACKGC AGMGCTTCWCCTTCWACRTCYTC ATTACTCAGAGTGCTTCTCARTG TGCATACCRTCAGTYTTTTCAAC	Takano et al.^36^
Proteobacteria	16S rRNA gene	Broad-range assay (end point PCR)	Rick16SF1 Rick16SR4	GTATGCTTAACACATGCAAGTCGAAC TCCGCGATTACTAGCGATTCC	Weisburg et al.^37^

PCR = polymerase chain reaction; rRNA = ribosomal RNA; SFG = spotted fever group.

### Detection of *Rickettsia* DNA.

Individual tick DNA samples were tested using SYBR Green PCR targeting the 547-701-nucleotide (nt) fragment of *omp*A of the SFG rickettsiae.^32^ Each 20-μL PCR reaction contained 4 μL of tick DNA, 0.0625 mM final concentration of each forward and reverse primer, 3 mM magnesium chloride, 1 mM deoxynucleoside triphosphate, and 2 μL of 10× SYBR Green Master Mix. DNA from *Rickettsia montanensis* strain OSU85-930 or *R. sibirica* strain 246 grown in VERO E6 cells were used as positive controls; sterile distilled water was used as a negative control. All reactions were run for 50 cycles, followed by melting curve analysis of the amplicon. DNA from samples testing positive for the SYBR Green OmpA gene fragment was analyzed further using conventional or semi-nested PCR to amplify longer portions of *omp*A, *omp*B, *sca*4, and *glt*A according to previously described protocols (Table 1).^33^ PCR results were evaluated by electrophoresis in 1.2% agarose gels stained with 0.5 μg/mL ethidium bromide.

### Detection of Anaplasmataceae DNA.

A portion of the 16S rRNA gene of Anaplasmataceae was detected with a SYBR Green PCR assay and melting curve analysis.^34^^,^^35^ Setup and data acquisition was similar to those described for SFG rickettsiae, with the exception of the specific primers. DNA from *Ehrlichia chaffeensis* Arkansas grown in the canine macrophage cell line DH82 was used as a positive control; sterile distilled water was used as a negative control. Tick DNA samples testing positive were analyzed further with a nested PCR assay targeting the *gro*EL gene of Anaplasmataceae and broad-range 16S rRNA gene PCR.^36^^,^^37^

### Sequencing and sequence analysis.

Individual amplicons, including *omp*A, *omp*B, *sca*4, and *glt*A fragment genes of *Rickettsia*, the *gro*EL amplicon of Anaplasmataceae as well as selected tick gene amplicons of the expected sizes were excised from the gel and the DNA was recovered using Wizard PCR Preps according to the manufacturer’s instructions (Promega, Madison, WI). The purified amplicons were sequenced in both directions using PCR primers and the ABI PRISM BigDye^TM^ Terminator Cycle 3.1 Sequencing kit (Applied Biosystems, Bedford, MA). Sequencing reads were edited and contigs were assembled using Sequencher 5.3 (Gene Codes, Ann Arbor, MI). Primer sequences were removed from assembled contigs, and sequences were analyzed using the National Center for Biotechnology Information (NCBI) Basic Local Alignment Tool (BLAST) search engine. Unique sequences generated during this study were submitted to NCBI GenBank under the following accession nos.: MZ546455-MZ546485, tick 12S mitochondrial rRNA gene; MZ965079-MZ965083, tick ITS2 region; MZ970599-MZ970604, tick COII gene; MZ965077-MZ965078, *Coxiella* endosymbiont 16S rRNA gene; MZ970589-MZ97059, *Ehrlichia* and *Anaplasma gro*EL gene; MZ970593-MZ970598, *Rickettsia glt*A; MZ970572-MZ970588, *Rickettsia omp*A; MZ970567-MZ970572, *Rickettsia omp*B; and MZ970562-MZ970565, *Rickettsia sca*4.

### Multiple sequence alignment and phylogenetic analyses.

Multiple sequence alignment and phylogenetic analyses were conducted in MEGA X.^38^ Each alignment included nucleotide sequences of validated and *Candidatus* species of *Rickettsia* or Anaplasmataceae, and the nearest BLAST hits without standing in taxonomy. The evolutionary history was inferred using the neighbor-joining method. The percentages of replicate trees in which the associated taxa clustered together were determined using 500 replicate bootstrap test and are indicated next to the branches. Each tree was drawn to scale, with branch lengths in the same units as those of the evolutionary distances used to infer the phylogenetic tree. The evolutionary distances were computed using the Kimura 2-parameter method and are in the units of the number of base substitutions per site. All ambiguous positions were removed for each sequence pair (pairwise deletion option).

### Statistical analysis.

Statistical analysis was carried out using the Z-test to compare two population proportions, designating a population as individuals that have the characteristic in question. Statistical significance was set at *P* < 0.05. CIs for prevalence rates in adults and nymphs were calculated using the Wilson score method without continuity correction.^39^

## RESULTS

### Tick collection and host associations.

A total of 847 tick specimens from central Sri Lanka were examined as a part of this study, including 94 males, 99 females, 46 nymphs, and 608 larvae (Table 2). Larvae were mostly collected by flagging (*n* = 603), and were comprised primarily of *Amblyomma* sp. (99.5%, *n* = 600) and a few *Haemaphysalis* and *Rhipicephalus*. Adult *Amblyomma* sp. ticks were removed from two tortoises, one pangolin, and three dogs. *Rhipicephalus* sp. adults and nymphs were mostly from dogs (66%, *n* = 118 *Rhipicephalus* from animals) and cattle (33%). *Haemaphysalis* sp. adults and nymphs were mostly from cattle (77%, *n* = 101 of *Haemaphysalis* from animals), whereas the remaining few samples were from dogs, goats, and pigs.

**Table 2:** Summary of ticks examined, their locations in Sri Lanka, and vertebrate hosts

Location	Tick genus	Host (source)	Ticks, *n*	Male, *n*	Female, *n*	Nymphs, *n*	Larvae, *n*	Positive for SFG	Positive for Ehr/An	Molecular identification
Hemmathagama	*Haemaphysalis*	Flagging	4	0	0	2	2	0	0	–
		Cow (2)	17	8	8	1	0	4	0	–
		Dog (1)	2	2	0	0	0	0	0	–
	*Rhipicephalus*	Flagging	1	0	0	0	1	0	0	–
		Cow (1)	1	1	0	0	0	0	0	–
		Dog (2)	3	3	0	0	0	2	0	*R. massiliae-*like (SL148)
		Goat (1)	2	2	0	0	0	1	1	*R. kellyi* (SL154*)*
Galaha	*Haemaphysalis*	Goat (3)	5	0	1	4	0	0	1	–
	*Rhipicephalus*	Cow (2)	28	10	18	0	0	1	7	*Ehrlichia* sp. (SL193|SL199)
Gambola	*Amblyomma*	Dog (1)	1	0	1	0	0	0	1	–
	*Haemaphysalis*	Pig (1)	6	0	0	1	5	1	0	–
	*Rhipicephalus*	Dog (2)	2	2	0	0	0	0	1	–
Minuwangoda	*Haemaphysalis*	Cow (2)	47	5	39	3	0	0	3	–
		Dog (2)	10	7	2	1	0	0	1	–
	*Rhipicephalus*	Dog (3)	24	7	5	12	0	0	4	–
Peradeniya Farm	*Amblyomma*	Monkey (2)	5	5	0	0	0	0	0	–
		Pangolin (1)	4	4	0	0	0	0	1	*Ehrlichia* sp. (SL91)
	*Haemaphysalis*	Cow (4)	14	0	6	8	0	1	0	–
	*Rhipicephalus*	Cow (1)	1	0	0	1	0	1	0	*R. massiliae-*like (SL261)
		Dog (1)	3	2	0	1	0	1	0	*R. massiliae-*like (SL275)
Wasgamuwa Park	*Amblyomma*	Flagging	600	0	0	0	600	45	2	*R. africae* (8 larva pools)
		Dog (2)	2	0	1	1	0	1	0	–
		Tortoise (2)	8	7	1	0	0	1	1	*Anaplasma* sp. (SL103)
	*Rhipicephalus*	Cow (1)	9	5	4	0	0	0	0	–
		Dog (7)	48	24	13	11	0	7	0	*R. massiliae* (SL141|SL277), *Rickettsia* Pakistani type (SL94)
Total	Ticks from 3 genera	–	847	94	99	46	608	65 (7.7%)	23 (2.7%)	–

Ehr/An = *Ehrlichia* and *Anaplasma*; SFG = spotted fever group rickettsiae.

Analysis of 12S mitochondrial rRNA fragment sequences identified dog-infesting *Rhipicephalus* ticks as *R. haemaphysaloides* (98% of sequence identity to NCBI accession no. MW080207) and the tropical lineage of *Rhipicephalus sanguineus* (99% of sequence identity to AY559842). *Rhipicephalus* ticks removed from cattle were identified as *Rhipicephalus annulatus* (99% sequence identity to EU921773). The 12S mitochondrial rRNA fragment sequences generated for *Haemaphysalis* ticks were similar to each other and were related most closely but were not identical to a homologous sequence of the 12S mitochondrial rRNA fragment from *Haemaphysalis flava* (90% of sequence identity to JF58621) and *Haemaphysalis longicornis* (90.62% of sequence identity to MK450606). The 12S mitochondrial rRNA fragment sequences from adult *Amblyomma* ticks removed from tortoise and pangolin differed from each other and represented two unique genotypes that do not have significant matches to homologous sequences from other *Amblyomma* species available from the NCBI GenBank database. The third 12S mitochondrial rRNA fragment sequence genotype was detected in *Amblyomma* larvae, and this sequence also did not match any existing sequences searchable by BLAST though the NCBI GenBank. Because the existing database of sequences for the COII tick gene and the ITS2 spacer region fragment is even less robust, sequencing these fragments for Sri Lankan ticks did not contribute to their specific molecular identification.

### Testing ticks for SFG rickettsiae.

Sixty-five of 304 tick DNA samples tested positive using OmpA gene SYBR Green assay (21.4%; 95% CI, 17.1–26.) (Table 2). Most of these positive samples were DNA from 62 pools of *Amblyomma* larvae; 45 pools tested positive, with an estimated minimum infection rate of 7.5% (95% CI, 5.6–9.9). Only 2 of 20 adult *Amblyomma* tick DNAs tested positive for the OmpA gene (10%; 95% CI, 2.8–30.1). Thirteen of 118 *Rhipicephalus* tick DNAs tested positive for the OmpA gene (11.02%; 95% CI, 6.6–17.9). SFG *Rickettsia* DNA was detected at a similar rate in *Haemaphysalis* tick DNAs when compared with *Rhipicephalus* tick DNAs (z = –1.4947, *P* < 0.05), as it was present in 6 of 100 DNAs tested (6%; 95% CI, 2.8–12.5). SFG rickettsiae belonging to five different genotypes were identified in these samples using multiple-locus sequencing.

**Figure 1.**
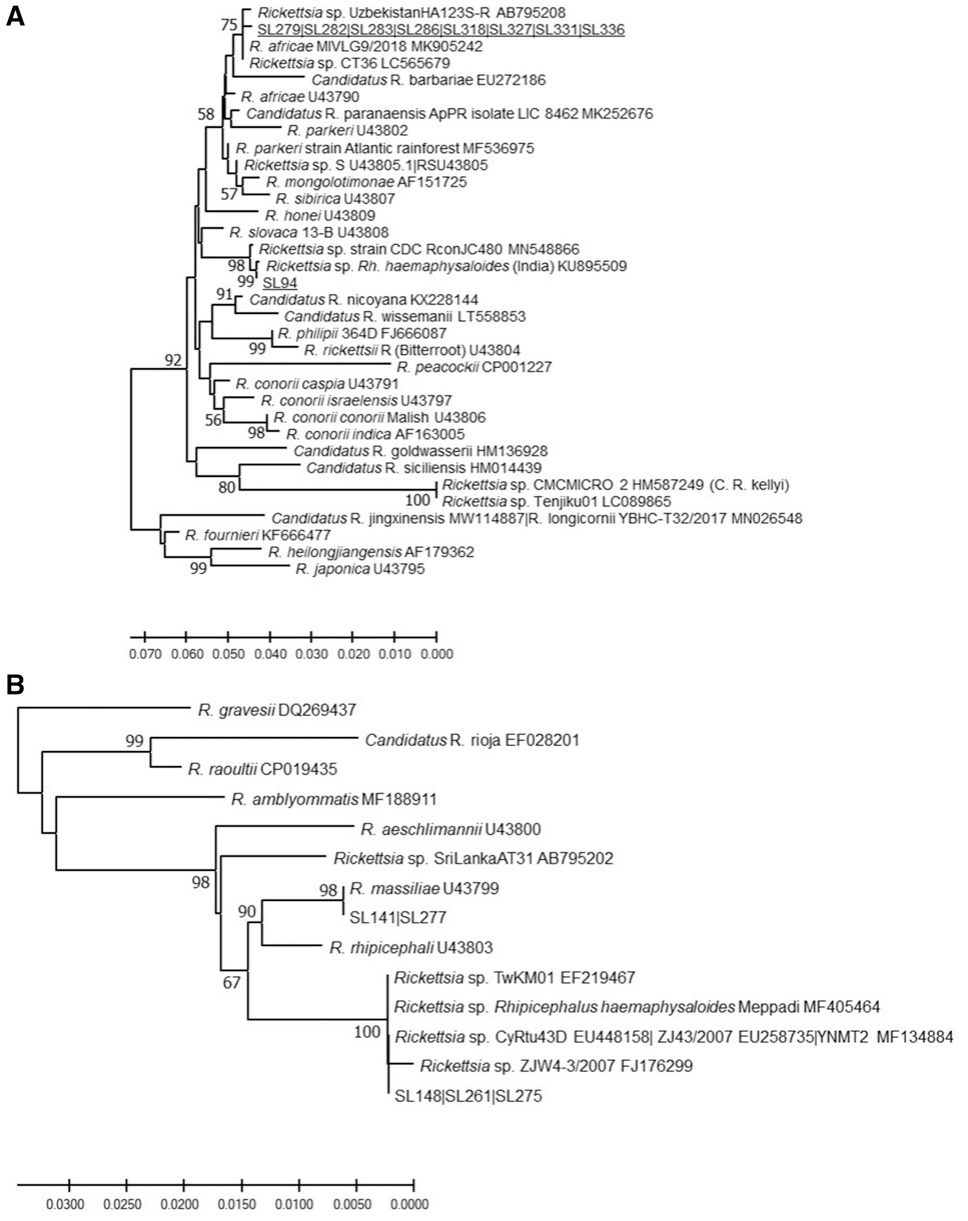
Sub-trees demonstrating (**A**) the genetic relationship of *Rickettsia omp*A genes from *Amblyomm*a larval pools and SL94 Pakistani-like isolate, and (**B**) the genetic relationship of *Rhipicephalus massiliae* and *R. massiliae*-like isolates detected in Sri Lankan ticks. Samples of Sri Lankan ticks are indicated by the letters SL and corresponding tick number, and are underlined. The evolutionary history was inferred using the neighbor-joining method computed using the Kimura 2-parameter method in MEGA X.^38^ This analysis involved 57 nucleotide sequences of validated and *Candidatus* species of *Rickettsia*, and the nearest Basic Local Alignment Tool hits of *Rickettsia* without standing in taxonomy. There were 540 positions in the final data set.

Sequences of amplicons of the 70-602-nt OmpA gene fragment from DNA from eight *Amblyomma* larval pools (SL279, SL282, SL283, SL286, SL318, SL327, SL331, and SL336) were identical to each other and had significant sequence similarity (99%, three single nucleotide polymorphisms [SNPs]) to the homologous fragment from *Rickettsia africae* ESF5-type strain (U43790) from *Amblyomma variegatum* from Ethiopia. There was one A|G SNP at the 23rd nucleotide, which resulted in a Q|R-predicted amino acid mutation in the corresponding sequence of the *R. africae* detected in *Amblyomma* larvae from Sri Lanka. Phylogenetic analysis based on the *omp*A fragment placed these Sri Lankan rickettsiae in a separate lineage from the ESF5 isolate, together with other molecular isolates of *R. africae* (one SNP difference only) detected in other ticks across broad geographic regions including India (*Haemaphysalis* larvae), Uzbekistan (*Hyalomma aegypticum*), Zambia (unspecified tick), and from Egypt (two SNP differences) (*H. Hyalomma dromedarii *and* Hyalomma impeltatum*) (Figure 1A and Supplemental Figure S1).

Amplicons of the 70-602-nt OmpA gene fragment each derived from the DNAs of *R*. *haemaphysaloides* ticks from two different dogs (SL141, SL277) from the Wasgamuwa site were identical to each other and had 100% sequence similarity with a homologous OmpA gene fragment of *Rhipicephalus massiliae* Mtu5. Three other *R*. *haemaphysaloides* from dogs and cattle (SL148, SL261, SL275) from two different locations yielded an OmpA gene fragment with a nucleotide sequence with the greatest similarity to *R. massiliae* among *Rickettsia* with a recognized species status (98%). There were six SNPs and a 3-nt INDEL in *Rickettsia* sequences from Sri Lanka, resulting in six amino acid differences from *R. massiliae* Mtu5. OmpA fragment sequences (SL148, SL261, SL275) identical to those detected in the ticks from Sri Lanka have been detected previously in *Rhipicephalus turanicus* from Cyprus, *R*. *haemaphysaloides* from deer (described as an endosymbiont) in India and Taiwan (TwKM01 EF219467), and an unidentified cattle-biting tick in Yunnan Province, China (Figure 1B and Supplemental Figure S1). This agent was confirmed to be closely related to TwM01 by sequencing *omp*B and *sca*4 fragments from SL148 and SL275 ticks. A similar but not identical agent called *Rickettsia* sp. SriLankaAT31-R has been detected recently in *Amblyomma trimaculatum* found on snakes (*Boiga forsteni*) from one of the breeding facilities in Sri Lanka.^40^

**Figure 2.**
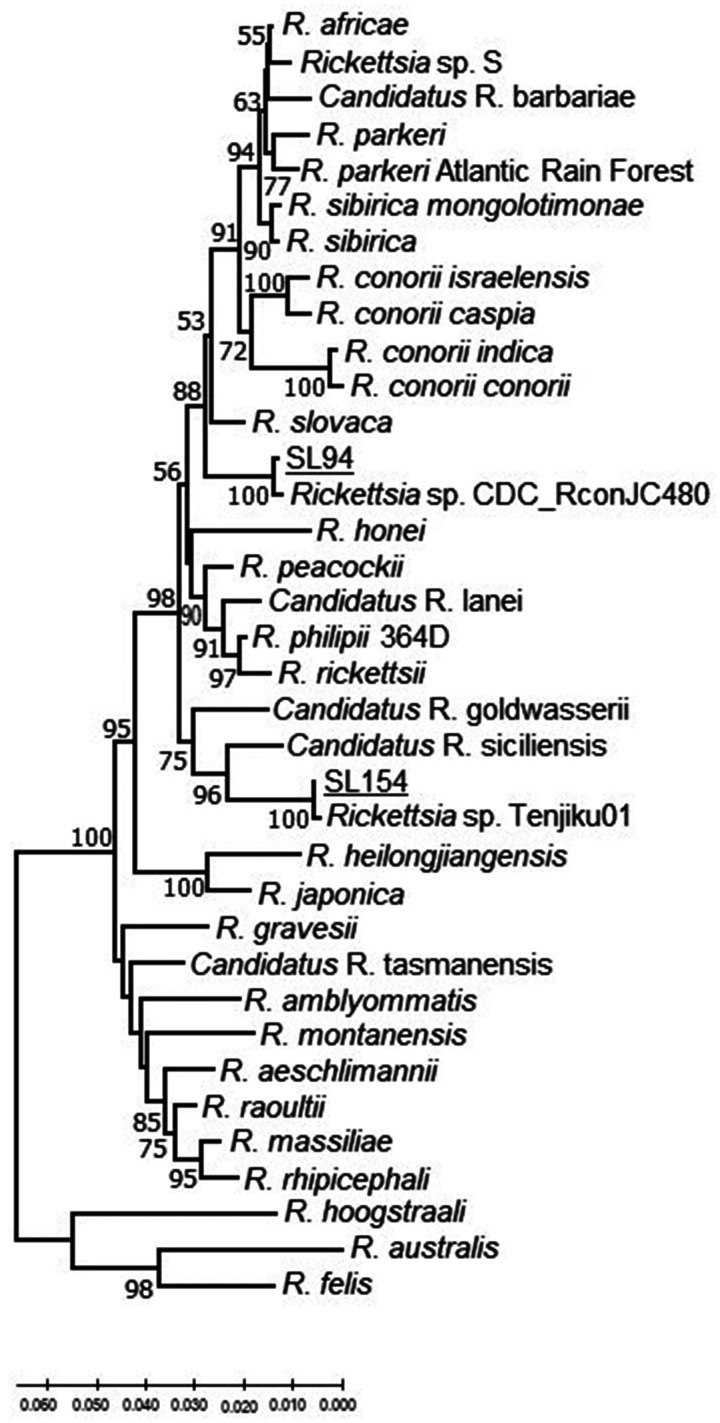
Genetic relationships of *Candidatus R. kellyi* (SL154) and *Rickettsia* SL94 detected in Sri Lanka based on concatenated *glt*A–*omp*B fragments. Samples of Sri Lankan ticks are indicated by the letters SL and corresponding tick number, and are underlined. The evolutionary history was inferred using the neighbor-joining method computed using the Kimura 2-parameter method in MEGA X.^38^ This analysis involved 38 nucleotide sequences of validated and *Candidatus* species of *Rickettsia*, and the nearest Basic Local Alignment Tool hits of *Rickettsia* without standing in taxonomy. There were 1,147 positions in the final data set.

The nucleotide sequence of a 111-nt small OmpA gene fragment amplified from *R*. *haemaphysaloides* (SL154) removed from a goat in Hemmathagama had 100% identity to the sequence from a homologous fragment from uncultured *Candidatus R. kellyi* (DQ08005.1) (Supplemental Figure S2). Unfortunately, further attempts to amplify a larger portion of the OmpA gene failed. However, larger fragments of the OmpB gene and *glt*A were amplified from the same tick DNA and thus presumably from the same SFG *Rickettsia.* Analysis of these two concatenated gene fragments showed that this SFG *Rickettsia* represents a unique lineage within the SFG rickettsiae that is most related to the uncultured *Candidatus R. siciliensis* identified previously in *R. turanicus* from Sicily^41^ (Figure 2 and Supplemental Figure S1). The OmpA gene fragment sequence of *Candidatus R. siciliensis* has a 93.88% sequence identity with *Rickettsia* sp. CMCMICRO2 (HM587249), detected in a febrile patient and four other similar cases (HM587248, HM587250, HM587251, HM587253) in southern India.^42^ These are almost 99% identical to the small *omp*A sequence fragment listed for *Candidatus R. kellyi*^43^ and 100% to *Rickettsia* sp. Tenjiku01 (LC089865) associated with a travel case to India.^44^ Thus, it appears these four agents are closely related (Supplemental Figure S2).

**Figure 3.**
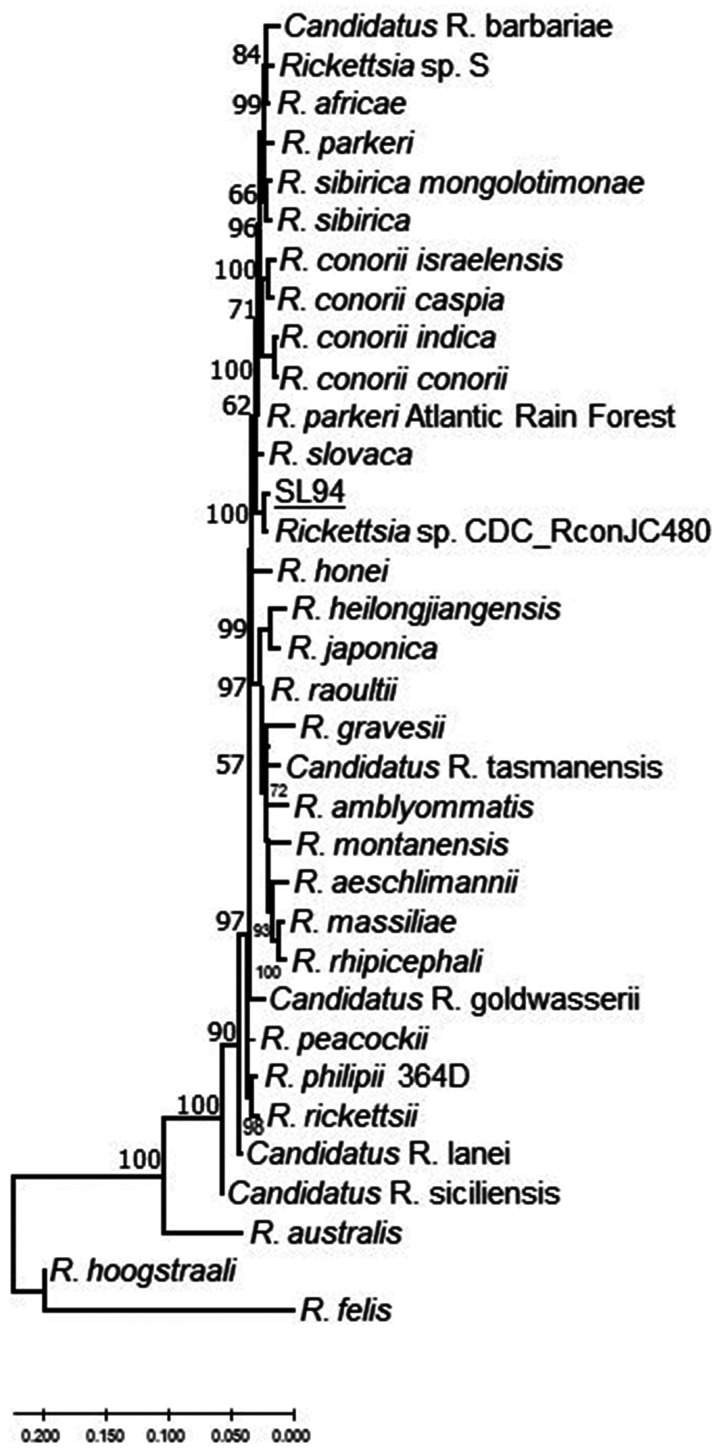
Genetic relationship of *Rickettsia* SL94 detected in Sri Lanka based on concatenated *glt*A–*sca*4–*omp*B fragments. Samples of Sri Lankan ticks are indicated by the letters SL and corresponding tick number, and are underlined. The evolutionary history was inferred using the neighbor-joining method computed using the Kimura 2-parameter method in MEGA X.^38^ This analysis involved 36 nucleotide sequences of validated and *Candidatus* species of *Rickettsia*, and the nearest Basic Local Alignment Tool hits of *Rickettsia* without standing in taxonomy. There were 2,099 positions in the final data set.

The OmpA gene fragment sequence (SL94) from a *R*. *haemaphysaloides* found on a dog in Wasgamuwa Park had the greatest sequence similarity to the homologous *omp*A fragment of *Rickettsia slovaca* (95%). Moreover, the high sequence homology is shared with a SFG *Rickettsia* detected in a *Rhipicephalus* sp. tick from Pakistan (*Rickettsia* sp. CDC_RconJC480 MN548866) and a *R. haemaphysaloides* tick from southern India (KU895509) (Figure 1A and Supplemental Figures S1 and S2). Further analysis of the concatenated fragments of *glt*A and *omp*B (Figure 2), and *glt*A-*sca*4-*omp*B (Figure 3) indicated that this SFG *Rickettsia* represents a new genetic type among currently known *Rickettsia*.

**Figure 4.**
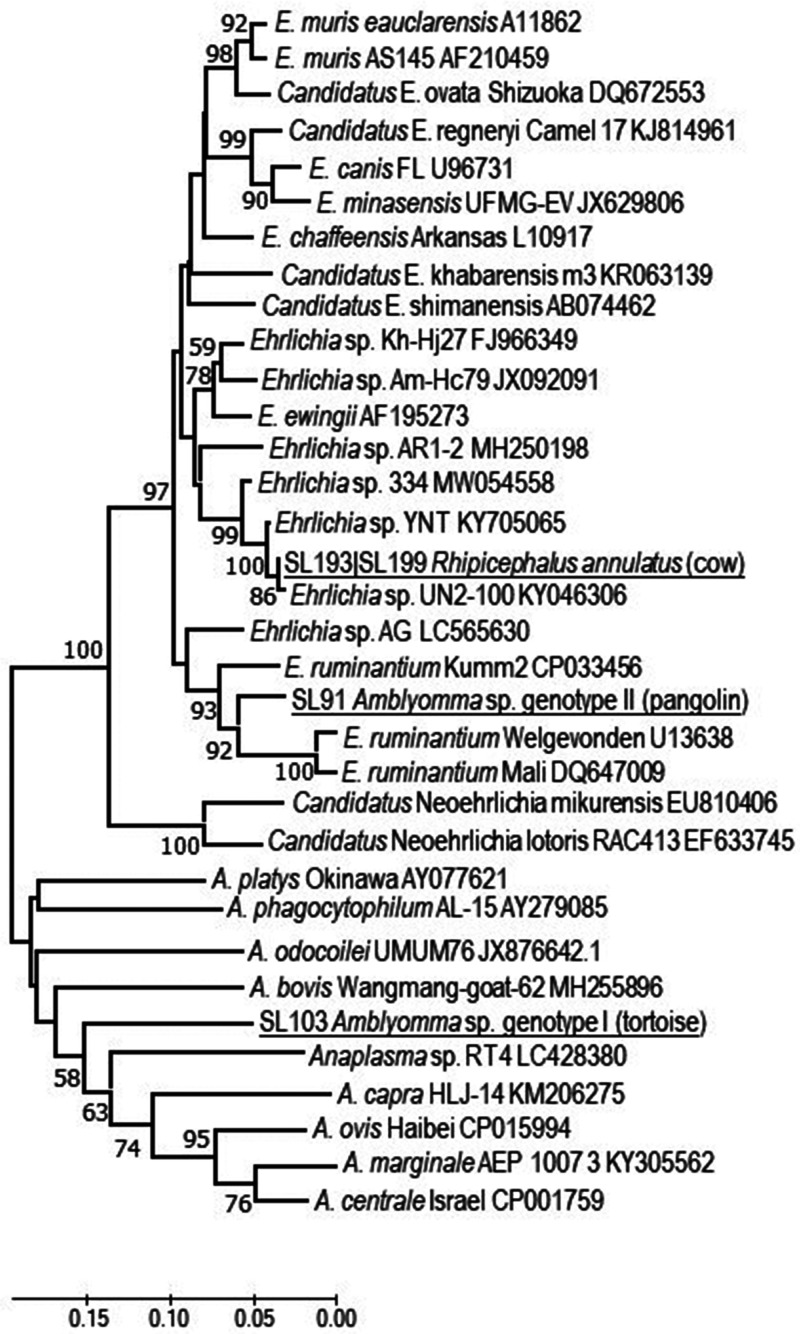
*gro*EL gene fragment genetic relationship of *Ehrlichia* and *Anaplasma* detected in ticks from Sri Lanka. Samples of Sri Lankan ticks are indicated by the letters SL and corresponding tick number, and are underlined. The evolutionary history was inferred using the neighbor-joining method computed using the Kimura 2-parameter method in MEGA X.^38^ This analysis involved 34 nucleotide sequences. There were 322 positions in the final data set.

### Testing ticks for Anaplasmataceae.

Twenty-three DNA samples tested positive for the Anaplasmataceae 16S rRNA gene fragment by SYBR Green PCR. Direct sequencing of the small PCR amplicon confirmed that the DNA detected belongs either to *Ehrlichia* or *Anaplasma*. Sequencing and analysis of the *gro*EL fragments determined that the *Amblyomma* sp. tick (SL103) removed from a tortoise in Wasgamuwa was infected with an *Anaplasma* that clustered together with various *Anaplasma* identified in association with livestock animals, but also with wild animals including reptiles. However, the genetic similarity was low, with the greatest at 80.82% similarity with a homologous RT4 *gro*EL fragment detected in *Amblyomma varanense* removed from a water monitor, *Varanus salvator*, in Indonesia (LC428380). One *Amblyomma* tick (SL91) removed from a pangolin brought to Peradeniya Farm tested positive for a *gro*EL fragment that had 91.3% (U13638) to 93.2% (CP033456) sequence similarity with various isolates of *Ehrlichia ruminantium*. Two *R. annulatus* (SL193, SL199) removed from a cow in Galaha tested positive for a *gro*EL fragment of an *Ehrlichia* sp. That is related to *Ehrlichia ewingii* (92.2%, AF195273); however, it clearly represents a unique lineage within that *Ehrlichia* cluster. Of note, this or closely related *gro*EL genotypes were found previously in *Rhipicephalus microplus* from Malaysia (KY046306) and Guinea (MW054558), and an unknown tick from Yunnan Province, China (KY705065); consequently, this *Ehrlichia* organism may circulate widely.

Analysis of the 16S rRNA gene amplicons derived from the tick DNA using the broad-range PCR Weisburg’s primers did not contribute to deciphering more completely the nature and genotype of the *Ehrlichia* and *Anaplasma* agents identified based on *gro*EL fragment sequences. The amplicons of most ticks were from *Morganella* and *Elizabethae Elizabethkingia meningoseptica*; however, several ticks (SL91, SL157) yielded amplicons matching those of a *Coxiella* endosymbiont detected previously in various species of *Haemaphysalis* and less frequently in *Rhipicephalus* ticks.

## DISCUSSION

Increasing numbers of reports of tick-transmitted rickettsial diseases in Sri Lanka have been published in the past two decades.^7^^–^^10^^,^^16^^,^^18^ These autochthonous cases occurred in diverse populations across the country. Traditionally, *Rickettsia conorii indica* was associated geographically with cases of spotted fever rickettsioses on the Indian subcontinent and Sri Lanka,^45^ and recently its etiological role has been confirmed in northern India.^46^ Consequently, most cases of suspected spotted fever rickettsiosis are diagnosed serologically as *R. conorii* infections using homologous antigens.^11^^–^^14^ However, at least one serological study used a cross-adsorption IFA protocol and concluded that exposure to more than one SFG rickettsiae may occur in Sri Lanka.^16^ This observation is in agreement with descriptions of three cases of travel-acquired rickettsioses in tourists returning from Sri Lanka and India to Australia, France, and Japan, indicating that *R*. *conorii* is probably not the sole etiological agent in Sri Lanka,^19^^,^^44^^,^^47^ We performed testing of ticks obtained in the vicinity of human dwellings and collected from peri-domestic animals to evaluate the presence of rickettsial agents posing potential risks to humans. The samples were mostly from cows and dogs, which can serve as sentinels of human exposure to tick-borne rickettsioses.^48^ Prior canine serological surveillance determined antibodies to SFG in 42% of dogs (*n* = 123) from Rajawatta, Thambavita, and areas of the western slopes and Unawatuna of Sri Lanka.^49^

The ticks we examined included three different types of *Amblyomma* ticks, one species of *Haemaphysalis* ticks, and at least three species of *Rhipicephalus* ticks based on sequencing the 12S mitochondrial rRNA gene fragment. Consequently, our survey must be considered only an initial snapshot of the tick-borne rickettsial agents found in Sri Lanka. Expanded tick collections (geography, temporal, and hosts), improved morphological keys for immature ticks, and a detailed molecular/morphological database are necessary to clarify the range of ticks in Sri Lanka that harbor rickettsial agents. Nonetheless, five different genotypes of SFG rickettsiae were identified in our sample of ticks: 1) a *R. africae*-like agent in *Amblyomma* larvae, 2) *R. massiliae* and a related genotype identified in association with *R. sanguineus* from dogs, 3) *R. haemaphysaloides* from dogs and cattle, 4) *Candidatus R. kellyi*, and 5) another novel genotype in *R. haemaphysaloides. Rickettsia massiliae* is known to be an infrequent human pathogen that may cause a severe form of rickettsiosis manifesting with typical eschar and purpuric or maculopapular rash, and bilateral chorioretinitis.^50^^,^^51^ Pathogenicity of the other closely related *R. massiliae*-like genotype is currently unknown; however, similar to *R. massiliae*, it appears to be broadly distributed worldwide based on an existing publication^52^ and many NCBI GenBank submissions of unpublished findings.

*Rickettsia africae*, the etiologic agent of African bite fever, is commonly vectored by *Amblyomma* sp. ticks in sub-Saharan Africa and the West Indies.^45^ Use of molecular tools expanded our current knowledge and findings of *R. africae* to northern Africa and middle eastern to western Asia, as well as its presence as a divergent clade in various species of *Hyalomma*,* Haemaphysalis*, and *Rhipicephalus* ticks.^45^ Interestingly, *R. africae* strains circulating in different geographic locations are not homogeneous because they exhibit genetic heterogeneity of the OmpA, OmpB, and Sca4 gene fragments used for genotyping.^53^ This suggests ongoing evolution and diversification of this widespread lineage of *Rickettsia*. We demonstrate that the divergent clade can also occur in different *Amblyomma* species in Sri Lanka. The impact of this process on the biological properties of *R. africae* and its role in the prevalence of human and animal exposures to rickettsiae and contribution to *Rickettsia*-caused diseases remain unknown. Because we found it only in ticks at one location and eschars, a common feature of classic African tick-bite fever reported rarely in this country, this *Rickettsia* may be introduced only recently to the island either with migratory birds or livestock.^54^^,^^55^ Nevertheless, local physicians should consider the diagnosis of African tick-bite fever in residents of Sri Lanka without a travel history to endemic areas for this agent.

*Candidatus R. kellyi* was first identified as an etiological agent in a pediatric patient from Thiruppathur, Tamil Nadu, India, who experienced a febrile illness with a maculopapular rash^43^; additional patients were identified subsequently in the same part of India.^42^ Furthermore, a new SFG rickettsiosis provisionally called *Candidatus R. indica* Tenjiku01 was diagnosed in a Japanese traveler returning from India,^44^ and this *Rickettsia* appears to be the same as *Candidatus R. kellyi*. Detection of *Candidatus R. kellyi* sequences (SL154) in our investigation indicates its presence in Sri Lanka, and it also provides the first record of the likely tick vector (*R. haemaphysaloides*) and an associated animal host (goat) involved in natural maintenance of this pathogen. It should be noted that *R. haemaphysaloides* is collected frequently from dogs and goats in Sri Lanka, thus suggesting that diverse peri-domestic animals may be a part of this tick’s natural cycle; this tick can also attach to people readily and then transmit rickettsiae during feeding.^24^

Another genotype of SFG rickettsiae (SL94) was also detected in *R. haemaphysaloides* collected from a dog. Genetic sequences of SL94 exhibited the greatest similarity to the yet-unnamed *Rickettsia* CDC_RconJC480 that was first isolated from a pool of *Rhipicephalus* sp. nymphs removed from *Nesokia indica* (short-tailed bandicoot rat) in West Pakistan.^56^ The original isolation was done in guinea pigs, and seroconversion and microscopic detection of *Rickettsia*-like organisms were used as a diagnostic tool to suggest it was related to *R. conorii*; however, seroconversions in guinea pigs were not commonly associated with any recognizable signs of infection. Subsequent typing using a so-called rickettsial toxin neutralization test in mice also identified RconJC480 as a strain of *R. conorii*.^57^ These findings preceded the recognition of *R. slovaca* as a human disease agent.^58^ More recent multilocus typing analysis using conventional rickettsial genes (*omp*A, *sca*4, *glt*A, the 17-kDa antigen gene, and *omp*B), additional variable *Rickettsia* genes (*atp*A, *vir*B4, *dna*A, *dna*K, and *rec*A), and four informative intergenic spacer region sites (*rrl*-*rrf*, *dks*A-*xer*C, *mpp*A-*pur*C, and *rpm*E-tRNA-fMet) indicated that RconJC480 represents a unique lineage within the SFG *Rickettsia* (G.A. Dasch, personal communication and corresponding sequences submitted to GenBank). Our analysis corroborates these observations and conclusions. A similar SFG *Rickettsia* has been detected in *R*. *haemaphysaloides* in Kerala state, India, thus further suggesting the role of *R*. *haemaphysaloides* in circulation of this *Rickettsia*. It is currently unknown whether RconJC480 is a human pathogen.

To the best of our knowledge, this study is the first effort to conduct direct molecular detection and identification of Anaplasmataceae in ticks from Sri Lanka. Three different types of *Anaplasma* and *Ehrlichia* were found in the ticks tested; however, it should be emphasized that only preliminary identification was completed for these organisms, and it was based on a relatively short fragment of the conserved *gro*EL gene. Therefore, these results should be interpreted with caution regarding the specific agents involved until additional data are available. *Anaplasma bovis* and related pathogens are widely distributed worldwide and can be found in association with different species of ticks including *Amblyomma*, and can be detected in different species of wild animals.^59^^–^^61^ In our research, we identified an *A. bovis*-like isolate in an *Amblyomma* tick collected from a tortoise. One possible explanation is that free-ranging tortoises scavenge for food around homesteads and interact with humans and livestock, and therefore may acquire ticks that would typically infest peri-domestic animals.^62^ Furthermore, the water monitor is a common reptile often encountered in city areas in Asian countries, and *kabaragoya* (the Sri Lankan water monitor) is the most common scavenger animal in the country and may share habitats with tortoises. The *Ehrlichia* sp. sequence clustering with *E. ruminantium* was detected in an *Amblyomma* tick from a pangolin. *Ehrlichia ruminantium* is the etiological agent of heartwater, a devastating illness affecting livestock in Africa and West Indies. *Ehrlichia* Panola Mountain agent found in *Amblyomma* ticks across the United States is closely related to *E. ruminantium*; however, it causes only mild clinical pathologies and pyrexia in experimentally infected goats.^63^ A new genotype of *Ehrlichia* related but distant from *E. ruminantium* was detected in jaguars and *Amblyomma* ticks from the Pantanal wetland of Brazil and from crab-eating foxes in southeastern Brazil.^64^^,^^65^ However, their identification was based on limited analysis of the 401-nt *dsb* gene fragment, which exhibits only 80.7% to 82.5% sequence similarity with the nearest known species, *E. ruminantium*.^64^^,^^65^

Last, the *Ehrlichia* sp. genotype detected in *R. annulatus* represents only a distant sister lineage to *E. ewingii*. *Ehrlichia ewingii* is a known human and canine pathogen transmitted by *Amblyomma americanum* in the United States; however, there is one report describing *dsb*-gene PCR detection and sequencing of *E. ewingii* in dogs from Cameroon,^66^ thus suggesting a broader distribution of this or related genotypes of *Ehrlichia*. Several other closely related *Ehrlichia* have been identified in other studies reported from Africa and Asia, so this *Ehrlichia* lineage may be distributed very broadly. *Ehrlichia canis* and *Anaplasma platys* have been reported in different parts of India and other southeastern Asian countries,^67^^–^^70^ but their presence in Sri Lanka is unknown. Further work is required to perform more in-depth analyses of these yet-uncultured *Ehrlichia* genotypes, and, most importantly, to determine their potential to cause human and veterinary diseases.

In conclusion, although this study demonstrated the presence of several SFG *Rickettsia* and Anaplasmataceae in ticks from Sri Lanka, some of which may also infect humans and peri-domestic animals, it is very likely that other agents of unknown pathogenicity are also present. National surveillance efforts must take into consideration this diversity of rickettsial agents, because they may exhibit different susceptibilities to therapeutic antibiotics such as rifampin resistance in *R. massiliae*.^33^^,^^71^ Diagnosis based solely on cross-reactivity with *R*. *conorii* antigen is inadequate to resolve the specific rickettsial etiology of human disease because of its poor specificity.^15^ Efforts to establish a well-characterized collection of regional isolates of *Rickettsia* and Anaplasmataceae are also important to improve the clinical diagnosis and surveillance of these diseases in Sri Lanka and southern India.

## Supplemental Material


Supplemental materials
