# Supplementary material for: Molecular Characterization of Rickettsial Agents in Ticks (Acari: Ixodidae) from Sri Lanka
Source: Am J Trop Med Hyg. 2022 Apr 11;106(6):1613–23. doi: 10.4269/ajtmh.21-0995 (PMC9209928; doi:10.4269/ajtmh.21-0995)
Supplement: Supplementary file 1 [file tpmd210995.SD1.pdf]

**Supplemental Figure 1: OmpA tree with all SFG *Rickettsia***

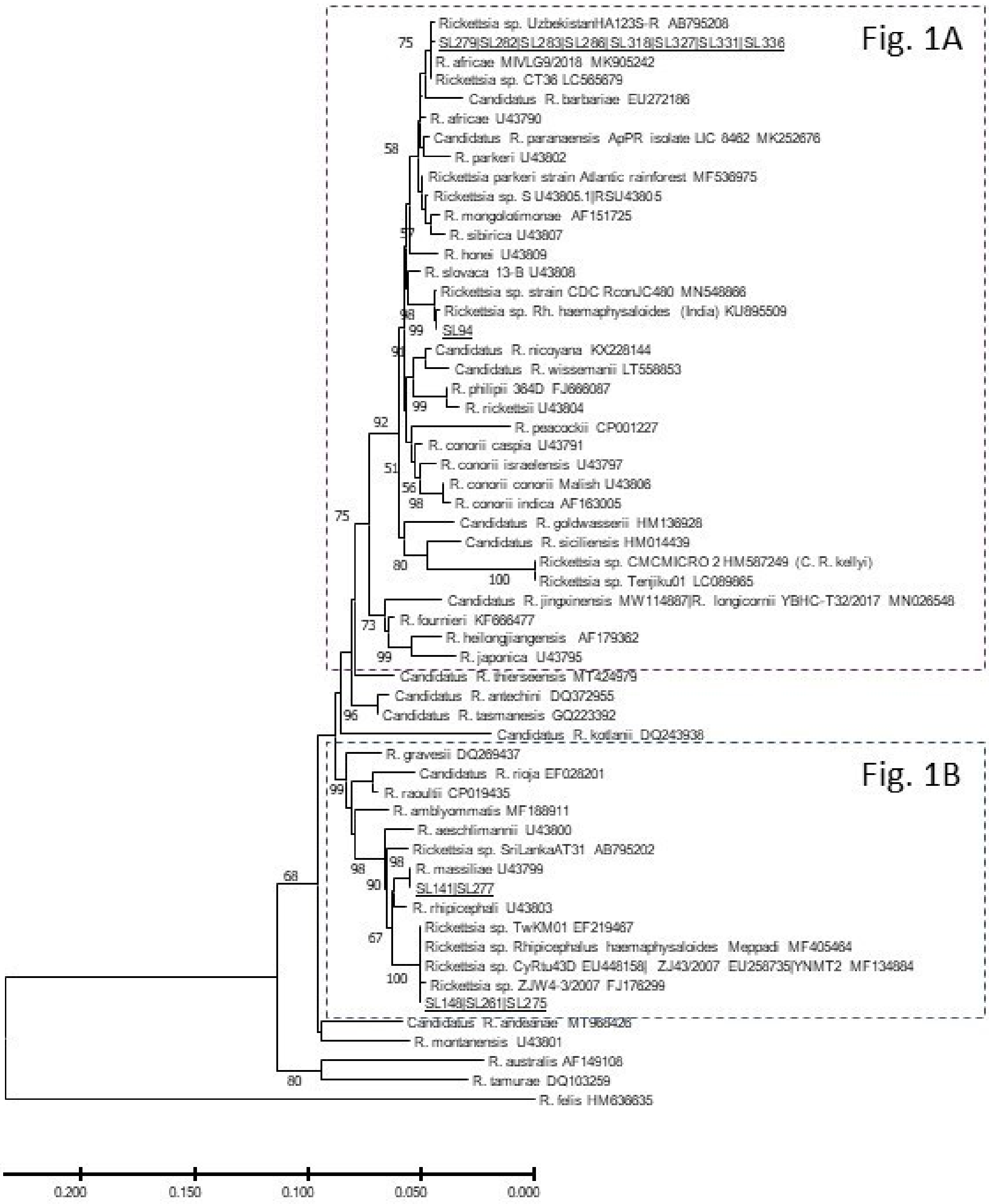


**Supplemental Figure 1:** OmpA gene fragment genetic relationship of SFG *Rickettsia* detected in ticks from Sri Lanka. Frames indicated by broken lines correspond to Figure 1A and Figure 1B shown in the main part of the manuscript. Samples of Sri Lankan ticks are indicated by letters SL and corresponding tick number and are underlined.

The evolutionary history was inferred using the Neighbor-Joining method computed using the Kimura 2-parameter method in MEGA X.[38] This analysis involved 56 nucleotide sequences. There were a total of 540 positions in the final dataset.

**Supplemental Figure 2. Comparisons of SL154 and SL94 with their closest gene sequences**

## Part A. SL154 sequence comparisons to Candidatus Rickettsia kellyi-related sequences

### *omp*A alignment

ompA_Tenjiku01_LC089865.1 sequence that matches ompA_Tenjiku01_NZ_BCMR01000011.1_21-1028 (100%) is underlined

```
ompA_Rkellyi_DQ080005.1             --------------------------------------------------
ompA_Tenjiku01_NZ_BCMR01000011      ATGGCGAATATTTCTCCAAAATTATTTCAAAAAGCAATACAACAAGGTCT
ompA_CMCMICRO2_HM587249.1           -TGGCGAATATTTCTCCAAAATTATTTCAAAAAGCAATACAACAAGGTCT
ompA_Rsiciliensis_HM014439.1        ---------------------------------GCAATACAACAAGGTCT
ompA_SL154                          --------------------------------------------------
                                     *************************************************

ompA_Rkellyi_DQ080005.1             ---------------------------TGCAGCCGAATATGCTGAGTAGT
ompA_Tenjiku01_NZ_BCMR01000011      TAAAGCCGCTTTATTCACCACCTCAACCGCAGC-GATAATGCTGAGTAGT
ompA_CMCMICRO2_HM587249.1           TAAAGCCGCTTTATTCACCACCTCAACCGCAGC-GATAATGCTGAGTAGT
ompA_Rsiciliensis_HM014439.1        TAAAGCCGCTTTATTCACCACTTCAACCGCAGC-GATAATGTTGAGTAGT
ompA_SL154                          --------------------------------------------------
                                    *************************** ***** *** *** ********

ompA_Rkellyi_DQ080005.1             AGCGGGGCACTCGGTGTTGCTGCAGGTTGTTATTGCTACTAATAATAATG
ompA_Tenjiku01_NZ_BCMR01000011      AGCGGGGCACTCGGTGTTGCTGCAGGT-GTTATTGCTACTAATAAT---G
ompA_CMCMICRO2_HM587249.1           AGCGGGGCACTCGGTGTTGCTGCAGGT-GTTATTGCTACTAATAAT---G
ompA_Rsiciliensis_HM014439.1        GGCGGGGCACTCGGTGTTGCTGCAGGT-GTTATTGCTACTACTAATAATG
ompA_SL154                          --------------------------------------------------
                                     ************************** ************* ****   *

ompA_Rkellyi_DQ080005.1             CAGCATTTAGGGATAATGTTAACAATAATAATTGGAATGAGATAACGGCT
ompA_Tenjiku01_NZ_BCMR01000011      CAGCATTTAGGGATAATGTTAACAATAATAATTGGAATGAGATAACGGCT
ompA_CMCMICRO2_HM587249.1           CAGCATTTAGGGATAATGTTAACAATAATAATTGGAATGAGATAACGGCT
ompA_Rsiciliensis_HM014439.1        CAGCATTTAGTGATAATGTTGGCAATAATAATTGGAATGAGATAACGGCT
ompA_SL154                          --------------------------------------------------
                                    ********** *********  ****************************

ompA_Rkellyi_DQ080005.1             GGAGGGGTAGCTAATGGTACTCCTGCTGGCGGTCCTCAAGACAATTGGGC
ompA_Tenjiku01_NZ_BCMR01000011      GGAGGGGTAGCTAATGGTACTCCTGCTGGCGGTCCTCAAGACAATTGGGC
ompA_CMCMICRO2_HM587249.1           GGAGGGGTAGCTAATGGTACTCCTGCTGGCGGTCCTCAAGACAATTGGGC
ompA_Rsiciliensis_HM014439.1        GAAGGGGTAGCTAATGGTACTCCTGCTGGCAGTCCTCAAAACAATTGGGC
ompA_SL154                          --------------------------------------------------
                                    * **************************** ******** **********

ompA_Rkellyi_DQ080005.1             ATTTACTTACGGTGGTAATTATGCTATCGATGCAGATGTAATCGGTCGTA
ompA_Tenjiku01_NZ_BCMR01000011      ATTTACTTACGGTGGTAATTATGCTATCGATGCAGATGTAATCGGTCGTA
ompA_CMCMICRO2_HM587249.1           ATTTACTTACGGTGGTAATTATGCTATCGATGCAGATGTAATCGGTCGTA
ompA_Rsiciliensis_HM014439.1        ATTTACTTACGGTGGTGATTATACTATCACTGCAGATGTAGCCGATCGTA
ompA_SL154                          --------------------------------------------------
                                    **************** ***** *****  **********  ** *****

ompA_Rkellyi_DQ080005.1             TTATTATGGCTATAAATGTTGCGGATACTAATCCTGTAAGTCTAAATATT
ompA_Tenjiku01_NZ_BCMR01000011      TTATTATGGCTATAAATGTTGCGGATACTAATCCTGTAAGTCTAAATATT
ompA_CMCMICRO2_HM587249.1           TTATTATGGCTATAAATGTTGCGGATACTAATCCTGTAAGTCTAAATATT
ompA_Rsiciliensis_HM014439.1        TTATTAAGGCTATAAATGTTGCGGGTACTAATCCCGTAGGTCTAAATATT
ompA_SL154                          --------------------------------------------------
                                    ****** ***************** ********* **  ***********

ompA_Rkellyi_DQ080005.1             ATGCAAAATACCACCGTTGGTTCGATTATAACGAGAGGTAACTTGTTGCC
ompA_Tenjiku01_NZ_BCMR01000011      ATGCAAAATACCACCGTTGGTTCGATTATAACGAGAGGTAACTTGTTGCC
ompA_CMCMICRO2_HM587249.1           ATGCAAAATACCACCGTTGGTTCGATTATAACGAGAGGTAACTTGTTGCC
ompA_Rsiciliensis_HM014439.1        ACTCAAAATACCACCGTTGGTTCGATTATAACGAGAGGTAACTTGTTGCC
ompA_SL154                          --------------------------------------------------
```

```
                                    *  ***********************************************

ompA_Rkellyi_DQ080005.1             TGTTACTATTACTGCCGGCCAAAACTTAACTTTAAACGGTACTAATGCTA
ompA_Tenjiku01_NZ_BCMR01000011      TGTTACTATTACTGCCGGCCAAAACTTAACTTTAAACGGTACTAATGCTA
ompA_CMCMICRO2_HM587249.1           TGTTACTATTACTGCCGGCCAAAACTTAACTTTAAACGGTACTAATGCTA
ompA_Rsiciliensis_HM014439.1        TGTTACTATTACTGCCGGCAAAAACTTAACTTTAAACGGTACTAATGCTG
ompA_SL154                          --------------------------------------------------
                                    ******************* *****************************

ompA_Rkellyi_DQ080005.1             TTGCTGCAAATCATGGTTTTGATGCTCCTGCCAATAATTATACAGGTTTA
ompA_Tenjiku01_NZ_BCMR01000011      TTGCTGCAAATCATGGTTTTGATGCTCCTGCCAATAATTATACAGGTTTA
ompA_CMCMICRO2_HM587249.1           TTGCTGCAAATCATGGTTTTGATGCTCCTGCCAATAATTATACAGGTTTA
ompA_Rsiciliensis_HM014439.1        TTGCTGCAAATCATGGTTTTAATGCTCCTGCCGATAATTATACAGGTTTA
ompA_SL154                          --------------------------------------------------
                                    ******************** *********** *****************

ompA_Rkellyi_DQ080005.1             GGAAATATATTTTTAGAGGGAGTGAATGCTGGAATAATTATAGACTCTAA
ompA_Tenjiku01_NZ_BCMR01000011      GGAAATATATTTTTAGAGGGAGTGAATGCTGGAATAATTATAGACTCTAA
ompA_CMCMICRO2_HM587249.1           GGAAATATATTTTTAGGGGGAGCGAATGCTGCACTC--------------
ompA_Rsiciliensis_HM014439.1        GGAAATATAGCTTT------------------------------------
ompA_SL154                          GGAAATATATTTTTAGAGGGAGTGAATGCTGGAATAATTATAGACTCTAA
                                    *********  ***** ***** ******** * * **************

ompA_Rkellyi_DQ080005.1             AGCCCCGGCAAAGATAACGCTTGCAGGAAATATAGATGGAGGAGGTATAA
ompA_Tenjiku01_NZ_BCMR01000011      AGCCCCGGCAAAGATAACGCTTGCAGGTAATACAGATGGAGGAGGTATAG
ompA_CMCMICRO2_HM587249.1           --------------------------------------------------
ompA_Rsiciliensis_HM014439.1        --------------------------------------------------
ompA_SL154                          AGCCCCGGCAAAGATAACGCTTGCAGGAAATATAGATGGAGGAGGTATAA
                                    *************************** **** ****************

ompA_Rkellyi_DQ080005.1             TAAC---TGTCAATACAGATGCTGCCA-----------------------
ompA_Tenjiku01_NZ_BCMR01000011      TAACGTTTACCGATAATAGTACAGTAACTGGCGATATAGGTAATACAAAT
ompA_CMCMICRO2_HM587249.1           --------------------------------------------------
ompA_Rsiciliensis_HM014439.1        --------------------------------------------------
ompA_SL154                          TAAC---TGTCAATAC----------------------------------
                                    ****   *  * ***    * * *  * *  **  *

ompA_Rkellyi_DQ080005.1             --------------------------------------------------
ompA_Tenjiku01_NZ_BCMR01000011      GCATTAGCAAAAGTGAATGTAGGAGCAGGTTTGCTACAAGTACAAGGCGG
ompA_CMCMICRO2_HM587249.1           --------------------------------------------------
ompA_Rsiciliensis_HM014439.1        --------------------------------------------------
ompA_SL154                          --------------------------------------------------


ompA_Rkellyi_DQ080005.1             --------------------------------------------------
ompA_Tenjiku01_NZ_BCMR01000011      AGTGGTAAAAGCGAATAGAATAAACTTAACGGATAATGCGTCAGCAGTGA
ompA_CMCMICRO2_HM587249.1           --------------------------------------------------
ompA_Rsiciliensis_HM014439.1        --------------------------------------------------
ompA_SL154                          --------------------------------------------------


ompA_Rkellyi_DQ080005.1             --------------------------------------------------
ompA_Tenjiku01_NZ_BCMR01000011      CATTTACGAATCCTGTAGTGGTGACCGGAGCGATAGATAATACCGGTAAT
ompA_CMCMICRO2_HM587249.1           --------------------------------------------------
ompA_Rsiciliensis_HM014439.1        --------------------------------------------------
ompA_SL154                          --------------------------------------------------


ompA_Rkellyi_DQ080005.1             --------------------------------------------------
ompA_Tenjiku01_NZ_BCMR01000011      GCAAATAATGGTATAGTAACGTTTACCGATAATAGTACAGTAACTGGCGA
ompA_CMCMICRO2_HM587249.1           --------------------------------------------------
ompA_Rsiciliensis_HM014439.1        --------------------------------------------------
ompA_SL154                          --------------------------------------------------
```

```
ompA_Rkellyi_DQ080005.1             --------------------------------------------------
ompA_Tenjiku01_NZ_BCMR01000011      TATAGGTAATACAAATGCATTAGCAAAAGTGAATGTAGGAGCAGGTTTGC
ompA_CMCMICRO2_HM587249.1           --------------------------------------------------
ompA_Rsiciliensis_HM014439.1        --------------------------------------------------
ompA_SL154                          --------------------------------------------------


ompA_Rkellyi_DQ080005.1             --------------------------------------------------
ompA_Tenjiku01_NZ_BCMR01000011      TACAAGTACAAGGCGGAGTGGTAAAAGCGAATAAAATAAACTTAACGGAT
ompA_CMCMICRO2_HM587249.1           --------------------------------------------------
ompA_Rsiciliensis_HM014439.1        --------------------------------------------------
ompA_SL154                          --------------------------------------------------


ompA_Rkellyi_DQ080005.1             --------------------------------------------------
ompA_Tenjiku01_NZ_BCMR01000011      AATGCGTCAGCAGTGACATTTACGAATCCTGTAGTGGTGACCGGGGAGAT
ompA_CMCMICRO2_HM587249.1           --------------------------------------------------
ompA_Rsiciliensis_HM014439.1        --------------------------------------------------
ompA_SL154                          --------------------------------------------------


ompA_Rkellyi_DQ080005.1             -------------
ompA_Tenjiku01_NZ_BCMR01000011      AAATAATACCGGT
ompA_CMCMICRO2_HM587249.1           -------------
ompA_Rsiciliensis_HM014439.1        -------------
ompA_SL154                          -------------
```

**_omp_B alignment**

ompB_Tenjiku-01 NZ_BCMR01000001.1:27401-32368
ompB_RickettsiaCMCMicro2_JN209833.1

```
ompB_SL154_MZ970569                 --------------------------------------------------
ompB_Rsiciliensis_HM014441.1        --------------------------------------------------
ompB_Tenjiku-01_NZ_BCMR0100000      ATGGCTCAAAAACCAAATTTTCTAAAAAAATTAATTTCCGCAGGGTTGGT
ompB_RickettsiaCMCMicro2_JN209      --------------------------------------------------


ompB_SL154_MZ970569                 --------------------------------------------------
ompB_Rsiciliensis_HM014441.1        ------CTCTACAGCTACCATAGTAGCCAGTTTTGCAGGTTCAGCTATGG
ompB_Tenjiku-01_NZ_BCMR0100000      AACTGCTTCTACAGCTACCATAGTAGCCAGCTTTGCAGGTTCAGCTATGG
ompB_RickettsiaCMCMicro2_JN209      --------------------------------------------------
                                           *******************************************

ompB_SL154_MZ970569                 --------------------------------------------------
ompB_Rsiciliensis_HM014441.1        GTGCGGCTATACAGCAGAATAGAACAACAAACGGAGTTGCTACAACTGTT
ompB_Tenjiku-01_NZ_BCMR0100000      GTGCTGCTATGCAGCAGAATAGAACAACAAACGGAGTTGCTACAACTGTT
ompB_RickettsiaCMCMicro2_JN209      --------------------------------------------------
                                    **** ***** ***************************************

ompB_SL154_MZ970569                 -------------------------------------------------C
ompB_Rsiciliensis_HM014441.1        GATGGTGCGGGATTTGACCCAACTGCCGGTCCTGCAAATGTTGCAGTTGC
ompB_Tenjiku-01_NZ_BCMR0100000      GATGGTGCGGGATTTGACCAAGCTGTCGGTCCTGCAAATGCTGCAGTTGC
ompB_RickettsiaCMCMicro2_JN209      --------------------------------------------------
                                    ******************* * *** ************** *********

ompB_SL154_MZ970569                 TCTAAATGCAGTTATTACTGCTAATGCTAATAATGGTATTGAGTTCAATA
ompB_Rsiciliensis_HM014441.1        TCTAAATTCAGTTATTACTGCTACTGCTAATAATGCTATTGATTTCAATA
ompB_Tenjiku-01_NZ_BCMR0100000      TCTAAATGCAGTTATTACTGCTAATGCTAATAATGGTATTGAGTTCAATA
```

```
ompB_RickettsiaCMCMicro2_JN209      --------------------------------------------------
                                    ******* *************** *********** ****** *******

ompB_SL154_MZ970569                 CTCCAGCCGGTAGTTTTAACGGTTTGTTTTTAGATACTGCAAACGCTTTA
ompB_Rsiciliensis_HM014441.1        CTCCAGCCGGTAGTTTTAACGGTTTGTTTTTAAATACTGCAAACCATTTA
ompB_Tenjiku-01_NZ_BCMR0100000      CTCCAGCCGGTAGTTTTAACGGTTTGTTTTTAGATACTGCAAACGCTTTA
ompB_RickettsiaCMCMicro2_JN209      --------------------------------------------------
                                    ******************************** ***********  ****

ompB_SL154_MZ970569                 GCAGTGACAGTGAGTGAAGATACTACCTTAGGGTTCATCACTAATACTGC
ompB_Rsiciliensis_HM014441.1        GCAGTGACAGTGAGTGAAGATACTACCTTAGGGTTCATCACTAATACTGC
ompB_Tenjiku-01_NZ_BCMR0100000      GCAGTGACAGTGAGTGAAGATACTACCTTAGGGTTCATCACTAATACTGC
ompB_RickettsiaCMCMicro2_JN209      --------------------------------------------------
                                    **************************************************

ompB_SL154_MZ970569                 TAATAATGGTCAGTTCTTTAACCTTACGCTTAATGCCGGTAAAACTCTTA
ompB_Rsiciliensis_HM014441.1        TAATAACGCTAACCTCTTTAACCTTACGCTTAATGCCGGTAAAACTCTTA
ompB_Tenjiku-01_NZ_BCMR0100000      TAATAATGGTCAGTTCTTTAACCTTACGCTTAATGCCGGTAAAACTCTTA
ompB_RickettsiaCMCMicro2_JN209      --------------------------------------------------
                                    ****** * * *  ************************************

ompB_SL154_MZ970569                 CTATAACAGGTCAAGGTATTACTAATGCACAAGCTGCTGCTACACAAAAT
ompB_Rsiciliensis_HM014441.1        CTATAACAGGTCAAGGTATTACTAATGCACAAGCTGCTGCTACACAAAAT
ompB_Tenjiku-01_NZ_BCMR0100000      CTATAACAGGTCAAGGTATTACTAATGCACAAGCTGCTGCTACACAAAAT
ompB_RickettsiaCMCMicro2_JN209      --------------------------------------------------
                                    **************************************************

ompB_SL154_MZ970569                 GCTAAAAATATTGTTACAGTAGTTAATAATGGTGCTGCTATTAA
ompB_Rsiciliensis_HM014441.1        GCTAAAAATGTTGTTGTAGCATTTAATAATGGTGCTGCTATTGA
ompB_Tenjiku-01_NZ_BCMR0100000      GCTAAAAATATTGTTACAGTAGTTAATAATGGTGCTGCTATTAA
ompB_RickettsiaCMCMicro2_JN209      --------------------------------------------
                                    ********* *****  ** * ******************** *

ompB_SL154_MZ970569                 CAATAATGATCTTAAAGGTGTAGGAAGAATAGACTTCGGTGCTGCGGCTT
ompB_Rsiciliensis_HM014441.1        CAATAATGATCTTAAAGGTGTAGGAAGAATAGACTTCGGTGCTGCGGCTT
ompB_Tenjiku-01_NZ_BCMR0100000      CAATAATGATCTTAAAGGTGTAGGAAGAATAGACTTCGGTGCTGCGGCTT
ompB_RickettsiaCMCMicro2_JN209      --------------------------------------------------
                                    **************************************************

ompB_SL154_MZ970569                 CTGTAGAATTTAATTTAGCACATCCTACAACTCAAGAAGCTCCTCTTATA
ompB_Rsiciliensis_HM014441.1        CTATAGTATTTAATTTAGCAAATCCTACAACTCAAAAAGCTCCTCTTATA
ompB_Tenjiku-01_NZ_BCMR0100000      CTGTAGAATTTAATTTAGCACATCCTACAACTCAAGAAGCTCCTCTTATA
ompB_RickettsiaCMCMicro2_JN209      ------ -------------------------------------------
                                    ** ***************** ************** **************

ompB_SL154_MZ970569                 CTTGGAGCTAATGCTGTAATAGTTAATGGTGTTAACGGTACATTAAATGT
ompB_Rsiciliensis_HM014441.1        CTTGGAGGTAATGCTGTAATAGTTAATGGTGTTAACGGTACATTAAATGT
ompB_Tenjiku-01_NZ_BCMR0100000      CTTGGAGCTAATGCTGTAATAGTTAATGGTGTTAACGGTACATTAAATGT
ompB_RickettsiaCMCMicro2_JN209      --------------------------------------------------
                                    ******* ******************************************

ompB_SL154_MZ970569                 TACTAATGGATTTATTCAAGTTTCAGATAACAGTTTTGCTACTGTTAAGG
ompB_Rsiciliensis_HM014441.1        TACTAATGGATTTATTCAAGTTTCAGATAAAAGTTTTGCTACTGTTAAGA
ompB_Tenjiku-01_NZ_BCMR0100000      TACTAATGGATTTATTCAAGTTTCAGATAACAGTTTTGCTACTGTTAAGG
ompB_RickettsiaCMCMicro2_JN209      --------------------------------------------------
                                    ****************************** ****************** 

ompB_SL154_MZ970569                 CAATTAATATCGGTGACGGTCAAGGTATCATATTCAATACTAATGCTAAT
ompB_Rsiciliensis_HM014441.1        CAATTAATATCGGTGACGGTCAAGGTATCATATTCAATACTAATGCTAAT
ompB_Tenjiku-01_NZ_BCMR0100000      CAATTAATATCGGTGACGGTCAAGGTATCATATTCAATACTAATGCTAAT
ompB_RickettsiaCMCMicro2_JN209      --------------------------------------------------
                                    **************************************************
```

```
ompB_SL154_MZ970569                 AATGCTAATGCTTTAAATTTACAAGCAGGTGGTACTACTATTAATTTTAA
ompB_Rsiciliensis_HM014441.1        AATGCTAATGCTTTAAATTTACAAGCAGGT---ACTACTATTAATTTTAA
ompB_Tenjiku-01_NZ_BCMR0100000      AATGCTAATGCTTTAAATTTACAAGCAGGTGGTACTACTATTAATTTTAA
ompB_RickettsiaCMCMicro2_JN209      --------------------------------------------------
                                    ******************************   *****************

ompB_SL154_MZ970569                 TGGAACAGACGG--------------------------------------
ompB_Rsiciliensis_HM014441.1        TGGAACAGACGGTACGGGTAGGTTAGTATTATTAAGTAAGAATG------
ompB_Tenjiku-01_NZ_BCMR0100000      TGGAACAGACGGTACGGGTAGGTTAGTATTATTAAGTAAGAATGCTGCTG
ompB_RickettsiaCMCMicro2_JN209      --------------------------------------------------
                                    ********************************************

ompB_SL154_MZ970569                 --------------------------------------------------
ompB_Rsiciliensis_HM014441.1        --------------------------------------------------
ompB_Tenjiku-01_NZ_BCMR0100000      CTACCGACTTTAACGTTACAGGAAGTTTAGGCGGTAATCTAAAAGGTATT
ompB_RickettsiaCMCMicro2_JN209      --------------------------------------------------

ompB_SL154_MZ970569                 --------------------------------------------------
ompB_Rsiciliensis_HM014441.1        --------------------------------------------------
ompB_Tenjiku-01_NZ_BCMR0100000      ATCGAATTTAACACTGTTACAGTCAACGGTCAACTTATAGCTAATGCAGG
ompB_RickettsiaCMCMicro2_JN209      --------------------------------------------------

ompB_SL154_MZ970569                 --------------------------------------------------
ompB_Rsiciliensis_HM014441.1        --------------------------------------------------
ompB_Tenjiku-01_NZ_BCMR0100000      TGGTCCTGCTAATGCAGTAATAGGTACTAATAATGGCGCAGGTAGAGCTG
ompB_RickettsiaCMCMicro2_JN209      --------------------------------------------------

ompB_SL154_MZ970569                 --------------------------------------------------
ompB_Rsiciliensis_HM014441.1        --------------------------------------------------
ompB_Tenjiku-01_NZ_BCMR0100000      CAGGATTTGTTGTTAGCGTAGATAATGGTAAGGTAGCAACAATCGATGGA
ompB_RickettsiaCMCMicro2_JN209      --------------------------------------------------

ompB_SL154_MZ970569                 --------------------------------------------------
ompB_Rsiciliensis_HM014441.1        --------------------------------------------------
ompB_Tenjiku-01_NZ_BCMR0100000      CAAGTTTATGCTAAAGACATGGTTATACAAAGTGCTAATGCAATAGGACA
ompB_RickettsiaCMCMicro2_JN209      --------------------------------------------------

ompB_SL154_MZ970569                 --------------------------------------------------
ompB_Rsiciliensis_HM014441.1        --------------------------------------------------
ompB_Tenjiku-01_NZ_BCMR0100000      GGTAAATTTTAGACAGATAGTTGACGTTGGTGCAGACGGTACTACTGCCT
ompB_RickettsiaCMCMicro2_JN209      --------------------------------------------------

ompB_SL154_MZ970569                 --------------------------------------------------
ompB_Rsiciliensis_HM014441.1        --------------------------------------------------
ompB_Tenjiku-01_NZ_BCMR0100000      TTAAAACAGCAGCTTCTAAAGTTGCAATAACCCAAAACTCAAACTTTGGT
ompB_RickettsiaCMCMicro2_JN209      --------------------------------------------------

ompB_SL154_MZ970569                 --------------------------------------------------
ompB_Rsiciliensis_HM014441.1        --------------------------------------------------
ompB_Tenjiku-01_NZ_BCMR0100000      ACTACTGATTTCGGTAATGTTGCAGCACAGATTACAGTGCCTGATACTAT
ompB_RickettsiaCMCMicro2_JN209      --------------------------------------------------

ompB_SL154_MZ970569                 --------------------------------------------------
ompB_Rsiciliensis_HM014441.1        --------------------------------------------------
ompB_Tenjiku-01_NZ_BCMR0100000      AACCCTTACCGGTAATTTTACAGGTGATGCTCGCAATCCCGGTAACACTG
```

```
ompB_RickettsiaCMCMicro2_JN209      --------------------------------------------------

ompB_SL154_MZ970569                 --------------------------------------------------
ompB_Rsiciliensis_HM014441.1        --------------------------------------------------
ompB_Tenjiku-01_NZ_BCMR0100000      CAGGTGTGATAACTTTTGATGCTAACGGTGCTTTAGCAAGTGCTAGTGCA
ompB_RickettsiaCMCMicro2_JN209      --------------------------------------------------

ompB_SL154_MZ970569                 --------------------------------------------------
ompB_Rsiciliensis_HM014441.1        --------------------------------------------------
ompB_Tenjiku-01_NZ_BCMR0100000      GATGCAAATGTTGCGGTGACAAATAATATTACAGCAATTGAAGCATCAGG
ompB_RickettsiaCMCMicro2_JN209      --------------------------------------------------

ompB_SL154_MZ970569                 --------------------------------------------------
ompB_Rsiciliensis_HM014441.1        --------------------------------------------------
ompB_Tenjiku-01_NZ_BCMR0100000      TGCTGGAGTTGTCCAATTATCAGGAACACATGCTGCCGAGTTACGTTTAG
ompB_RickettsiaCMCMicro2_JN209      --------------------------------------------------

ompB_SL154_MZ970569                 --------------------------------------------------
ompB_Rsiciliensis_HM014441.1        --------------------------------------------------
ompB_Tenjiku-01_NZ_BCMR0100000      GAAATGCCGGTTCTATCTTTAAACTTGCTGACGGTACAGTTATAAACGGT
ompB_RickettsiaCMCMicro2_JN209      --------------------------------------------------

ompB_SL154_MZ970569                 --------------------------------------------------
ompB_Rsiciliensis_HM014441.1        --------------------------------------------------
ompB_Tenjiku-01_NZ_BCMR0100000      AAAGTTAACCAAACTGCTCTTGTCGGCGGTGCTCTTGCAGGCGGTACTAT
ompB_RickettsiaCMCMicro2_JN209      --------------------------------------------------

ompB_SL154_MZ970569                 --------------------------------------------------
ompB_Rsiciliensis_HM014441.1        --------------------------------------------------
ompB_Tenjiku-01_NZ_BCMR0100000      TACCTTAGACGGTAGTGCTACAATTACCGGTGATATAGGGAACGCTGGCG
ompB_RickettsiaCMCMicro2_JN209      --------------------------------------------------

ompB_SL154_MZ970569                 --------------------------------------------------
ompB_Rsiciliensis_HM014441.1        --------------------------------------------------
ompB_Tenjiku-01_NZ_BCMR0100000      GTGCTGCTGCGTTACAAGCAATTACTTTAGCTAACGATGCTACAAAAACA
ompB_RickettsiaCMCMicro2_JN209      --------------------------------------------------

ompB_SL154_MZ970569                 --------------------------------------------------
ompB_Rsiciliensis_HM014441.1        --------------------------------------------------
ompB_Tenjiku-01_NZ_BCMR0100000      TTAACACTTGGTGGAGCAAATATTATCGGTGCTAATGGTGGAACGATTGA
ompB_RickettsiaCMCMicro2_JN209      --------------------------------------------------

ompB_SL154_MZ970569                 --------------------------------------------------
ompB_Rsiciliensis_HM014441.1        --------------------------------------------------
ompB_Tenjiku-01_NZ_BCMR0100000      TTTCCAAGCTAACGGTGGTACTATTAAATTAACAAGCACTCAAAATAATA
ompB_RickettsiaCMCMicro2_JN209      --------------------------------------------------

ompB_SL154_MZ970569                 --------------------------------------------------
ompB_Rsiciliensis_HM014441.1        --------------------------------------------------
ompB_Tenjiku-01_NZ_BCMR0100000      TTGTAGTTGATTTTGATTTAGCTATAACTACTGATCAAACAGGCGTTGTT
ompB_RickettsiaCMCMicro2_JN209      --------------------------------------------------
```

```
ompB_SL154_MZ970569                 --------------------------------------------------
ompB_Rsiciliensis_HM014441.1        --------------------------------------------------
ompB_Tenjiku-01_NZ_BCMR0100000      GATGCGAGTAGCCTAACAAATGCTCAAGCTTTAACTATTAAAGGTAAAAT
ompB_RickettsiaCMCMicro2_JN209      --------------------------------------------------

ompB_SL154_MZ970569                 --------------------------------------------------
ompB_Rsiciliensis_HM014441.1        --------------------------------------------------
ompB_Tenjiku-01_NZ_BCMR0100000      CGGTACTATTGGAGTTAATAATAAAACTCTTGGACAATTTAATATTGGCT
ompB_RickettsiaCMCMicro2_JN209      --------------------------------------------------

ompB_SL154_MZ970569                 --------------------------------------------------
ompB_Rsiciliensis_HM014441.1        --------------------------------------------------
ompB_Tenjiku-01_NZ_BCMR0100000      CAAGTAAAACAGCTTTAAGTAACGGTGATGTTGCTATTAACGAGTTAGTT
ompB_RickettsiaCMCMicro2_JN209      --------------------------------------------------

ompB_SL154_MZ970569                 --------------------------------------------------
ompB_Rsiciliensis_HM014441.1        --------------------------------------------------
ompB_Tenjiku-01_NZ_BCMR0100000      ATTGGAAATAACGGTGCAGTACAATTCGCGCATAATACTTATTTAATAAC
ompB_RickettsiaCMCMicro2_JN209      --------------------------------------------------

ompB_SL154_MZ970569                 --------------------------------------------------
ompB_Rsiciliensis_HM014441.1        --------------------------------------------------
ompB_Tenjiku-01_NZ_BCMR0100000      AAGCACTACAAATGCTGCCGGTCAAGGTAAAATAATATTTAATCCTGTTG
ompB_RickettsiaCMCMicro2_JN209      --------------------------------------------------

ompB_SL154_MZ970569                 --------------------------------------------------
ompB_Rsiciliensis_HM014441.1        --------------------------------------------------
ompB_Tenjiku-01_NZ_BCMR0100000      TAAATAATAATACAACTCTTGCAGCCGGTACAAATTTAGGTAGTGCTACA
ompB_RickettsiaCMCMicro2_JN209      --------------------------------------------------

ompB_SL154_MZ970569                 --------------------------------------------------
ompB_Rsiciliensis_HM014441.1        --------------------------------------------------
ompB_Tenjiku-01_NZ_BCMR0100000      AATCCACTTGCAGAAATTAATTTTGGATCTAAAGGGCTTAATGTTGATAC
ompB_RickettsiaCMCMicro2_JN209      --------------------------------------------------

ompB_SL154_MZ970569                 --------------------------------------------------
ompB_Rsiciliensis_HM014441.1        --------------------------------------------------
ompB_Tenjiku-01_NZ_BCMR0100000      TGTATTAAATGTTGGTGCAGGTGTAAATTTATATGCTACTAATATTACTA
ompB_RickettsiaCMCMicro2_JN209      --------------------------------------------------

ompB_SL154_MZ970569                 --------------------------------------------------
ompB_Rsiciliensis_HM014441.1        --------------------------------------------------
ompB_Tenjiku-01_NZ_BCMR0100000      CTACCGATGCTAACGTAGGTTCTTTCGTCTTTAATGCCGGTGGAACAAAT
ompB_RickettsiaCMCMicro2_JN209      --------------------------------------------------

ompB_SL154_MZ970569                 --------------------------------------------------
ompB_Rsiciliensis_HM014441.1        --------------------------------------------------
ompB_Tenjiku-01_NZ_BCMR0100000      ATAGTAAGCGGTACAGTCGGTGGACAGCAAGGTAATAAGTTTAATACTGT
ompB_RickettsiaCMCMicro2_JN209      --------------------------------------------------

ompB_SL154_MZ970569                 --------------------------------------------------
ompB_Rsiciliensis_HM014441.1        --------------------------------------------------
ompB_Tenjiku-01_NZ_BCMR0100000      AGCATTAGGTAACGGTACTACTGTTAAGTTCTTAGGTAATGCAACATTTA
```

```
ompB_RickettsiaCMCMicro2_JN209      --------------------------------------------------

ompB_SL154_MZ970569                 --------------------------------------------------
ompB_Rsiciliensis_HM014441.1        --------------------------------------------------
ompB_Tenjiku-01_NZ_BCMR0100000      ACGGTAACACTACAATTGTAGCTAATTCTACCTTACAAGTCAGTGGTAAC
ompB_RickettsiaCMCMicro2_JN209      --------------------------------------------------

ompB_SL154_MZ970569                 --------------------------------------------------
ompB_Rsiciliensis_HM014441.1        --------------------------------------------------
ompB_Tenjiku-01_NZ_BCMR0100000      TATACTGCAGACTTTGTTGCATCTGCCGATGGTACCGGTATAGTAGAATT
ompB_RickettsiaCMCMicro2_JN209      --------------------------------------------------

ompB_SL154_MZ970569                 --------------------------------------------------
ompB_Rsiciliensis_HM014441.1        --------------------------------------------------
ompB_Tenjiku-01_NZ_BCMR0100000      CGTTAACAGCGATCCTATTACCGTAACATTAAACAAACAAGTTGCACCTG
ompB_RickettsiaCMCMicro2_JN209      --------------------------------------------------

ompB_SL154_MZ970569                 --------------------------------------------------
ompB_Rsiciliensis_HM014441.1        --------------------------------------------------
ompB_Tenjiku-01_NZ_BCMR0100000      TTGTTAATGCTTTAAAACAAATAACGGTTTCTGGTCCCGGTAACGTAGTG
ompB_RickettsiaCMCMicro2_JN209      --------------------------------------------------

ompB_SL154_MZ970569                 --------------------------------------------------
ompB_Rsiciliensis_HM014441.1        --------------------------------------------------
ompB_Tenjiku-01_NZ_BCMR0100000      ATTAATGAGATAGGTAATGCAGGTAATTATCATGGTGCCGTGACTGATAC
ompB_RickettsiaCMCMicro2_JN209      --------------------------------------------------

ompB_SL154_MZ970569                 --------------------------------------------------
ompB_Rsiciliensis_HM014441.1        --------------------------------------------------
ompB_Tenjiku-01_NZ_BCMR0100000      GATTGCTTTTGAAAATTCAAGTTTAGGTGCAGCTTTATTCTTACCTAGAG
ompB_RickettsiaCMCMicro2_JN209      --------------------------------------------------

ompB_SL154_MZ970569                 --------------------------------------------------
ompB_Rsiciliensis_HM014441.1        --------------------------------------------------
ompB_Tenjiku-01_NZ_BCMR0100000      GTATTCCATTCAATGATGCAGGCAACAGAATTCCTTTAACAATTAAAAGT
ompB_RickettsiaCMCMicro2_JN209      --------------------------------------------------

ompB_SL154_MZ970569                 --------------------------------------------------
ompB_Rsiciliensis_HM014441.1        --------------------------------------------------
ompB_Tenjiku-01_NZ_BCMR0100000      ACCGTAGGTAATAAAACAGCTACAGGTTTTAATGTTCCTAGCGTGATTGT
ompB_RickettsiaCMCMicro2_JN209      --------------------------------------------------

ompB_SL154_MZ970569                 --------------------------------------------------
ompB_Rsiciliensis_HM014441.1        --------------------------------------------------
ompB_Tenjiku-01_NZ_BCMR0100000      TTTAGGTGTTGATAGTGTCATCGCTGACGGTCAAGTAATCGGTGATCAAA
ompB_RickettsiaCMCMicro2_JN209      --------------------------------------------------

ompB_SL154_MZ970569                 --------------------------------------------------
ompB_Rsiciliensis_HM014441.1        --------------------------------------------------
ompB_Tenjiku-01_NZ_BCMR0100000      ATAATATCGTAGGTCTAGGTCTTGGAAGCGATAACGGCATAATCGTTAAT
ompB_RickettsiaCMCMicro2_JN209      --------------------------------------------------
```

```
ompB_SL154_MZ970569                 --------------------------------------------------
ompB_Rsiciliensis_HM014441.1        --------------------------------------------------
ompB_Tenjiku-01_NZ_BCMR0100000      GCTACTACATTATATGCAGGTATCGGTACTGTAAACAATAATCAAGGTAC
ompB_RickettsiaCMCMicro2_JN209      --------------------------------------------------

ompB_SL154_MZ970569                 --------------------------------------------------
ompB_Rsiciliensis_HM014441.1        --------------------------------------------------
ompB_Tenjiku-01_NZ_BCMR0100000      TGTCACACTTAGCGGTGGTATTCCTAATATCCCTGGTACAGTTTATGGCT
ompB_RickettsiaCMCMicro2_JN209      --------------------------------------------------

ompB_SL154_MZ970569                 --------------------------------------------------
ompB_Rsiciliensis_HM014441.1        --------------------------------------------------
ompB_Tenjiku-01_NZ_BCMR0100000      TAGGCATAGGTATTGGCGCTCCACAGTTCAAGCAAGTAACGTTTACTACA
ompB_RickettsiaCMCMicro2_JN209      --------------------------------------------------

ompB_SL154_MZ970569                 --------------------------------------------------
ompB_Rsiciliensis_HM014441.1        --------------------------------------------------
ompB_Tenjiku-01_NZ_BCMR0100000      GACTATAACAACTTAGGTAATATTATTGCAACTAACGTAACAATTAATGA
ompB_RickettsiaCMCMicro2_JN209      --------------------------------------------------

ompB_SL154_MZ970569                 --------------------------------------------------
ompB_Rsiciliensis_HM014441.1        --------------------------------------------------
ompB_Tenjiku-01_NZ_BCMR0100000      CGGTGTAACTGTTACTACAGGCGGTATAGCAGGAACAGATTTTGACGGTA
ompB_RickettsiaCMCMicro2_JN209      --------------------------------------------------

ompB_SL154_MZ970569                 --------------------------------------------------
ompB_Rsiciliensis_HM014441.1        --------------------------------------------------
ompB_Tenjiku-01_NZ_BCMR0100000      AAATTACTCTTGGAAGTGTTAACGGTAACGGTAATGTAAGATTTGCTGAC
ompB_RickettsiaCMCMicro2_JN209      --------------------------------------------------

ompB_SL154_MZ970569                 --------------------------------------------------
ompB_Rsiciliensis_HM014441.1        --------------------------------------------------
ompB_Tenjiku-01_NZ_BCMR0100000      GGTATATTGTCTAATTCTACAAGTATGATTGGTACTACTAAAGCTAATAA
ompB_RickettsiaCMCMicro2_JN209      --------------------------------------------------

ompB_SL154_MZ970569                 --------------------------------------------------
ompB_Rsiciliensis_HM014441.1        --------------------------------------------------
ompB_Tenjiku-01_NZ_BCMR0100000      TGGTACTGTAACTTATTTAGGTAATGCATTCGTCGGTAATATAGGTGATT
ompB_RickettsiaCMCMicro2_JN209      --------------------------------------------------

ompB_SL154_MZ970569                 --------------------------------------------------
ompB_Rsiciliensis_HM014441.1        --------------------------------------------------
ompB_Tenjiku-01_NZ_BCMR0100000      CAGATACTCCTGTAGCTTCTGTTAGATTTACAGGTAGTGATAGTGGTGCA
ompB_RickettsiaCMCMicro2_JN209      --------------------------------------------------

ompB_SL154_MZ970569                 --------------------------------------------------
ompB_Rsiciliensis_HM014441.1        --------------------------------------------------
ompB_Tenjiku-01_NZ_BCMR0100000      GTATTACAAGGAAATATTTATTCACAAGTCATAGACTTTGGTACTTATCA
ompB_RickettsiaCMCMicro2_JN209      --------------------------------------------------

ompB_SL154_MZ970569                 --------------------------------------------------
ompB_Rsiciliensis_HM014441.1        --------------------------------------------------
ompB_Tenjiku-01_NZ_BCMR0100000      CTTAGGTATTTTAAATTCTAATGTAATTTTAGGCGGCGGTACTACTGCTA
```

```
ompB_RickettsiaCMCMicro2_JN209      --------------------------------------------------


ompB_SL154_MZ970569                 --------------------------------------------------
ompB_Rsiciliensis_HM014441.1        --------------------------------------------------
ompB_Tenjiku-01_NZ_BCMR0100000      TTAACGGTAAAATCGATCTTCTTACAAATACTTTAACATTTGCAAGTGGT
ompB_RickettsiaCMCMicro2_JN209      --------------------------------------------------


ompB_SL154_MZ970569                 --------------------------------------------------
ompB_Rsiciliensis_HM014441.1        --------------------------------------------------
ompB_Tenjiku-01_NZ_BCMR0100000      ACTTCAACATGGGGAAACAATACTTCTATTGAAACTACTTTAACATTAGC
ompB_RickettsiaCMCMicro2_JN209      --------------------------------------------------


ompB_SL154_MZ970569                 --------------------------------------------------
ompB_Rsiciliensis_HM014441.1        --------------------------------------------------
ompB_Tenjiku-01_NZ_BCMR0100000      AAACGGTAATATAGGTCACATCGTTATCGTGGAAGGTGCGCAAGTTAATG
ompB_RickettsiaCMCMicro2_JN209      --------------------------------------------------


ompB_SL154_MZ970569                 --------------------------------------------------
ompB_Rsiciliensis_HM014441.1        --------------------------------------------------
ompB_Tenjiku-01_NZ_BCMR0100000      CAACAACCACAGGAACTACAACCATTAACGTACAAGATAATGCCAATGCA
ompB_RickettsiaCMCMicro2_JN209      --------------------------------------------------


ompB_SL154_MZ970569                 --------------------------------------------------
ompB_Rsiciliensis_HM014441.1        --------------------------------------------------
ompB_Tenjiku-01_NZ_BCMR0100000      AATTTCAGTGGTACACAAACTTATACTTTAATCCAAGGTGGTGCTAGATT
ompB_RickettsiaCMCMicro2_JN209      --------------------------------------------------


ompB_SL154_MZ970569                 --------------------------------------------------
ompB_Rsiciliensis_HM014441.1        --------------------------------------------------
ompB_Tenjiku-01_NZ_BCMR0100000      TAATGGTACTTTAGGAGGTCCCAACTTTGCTGTAACCGGAAGTAATCGTT
ompB_RickettsiaCMCMicro2_JN209      --------------------------------------------------


ompB_SL154_MZ970569                 --------------------------------------------------
ompB_Rsiciliensis_HM014441.1        --------------------------------------------------
ompB_Tenjiku-01_NZ_BCMR0100000      TCGTAAATTATAGTTTAATACGTGCTGCTAACCAAGATTATGTAATAACA
ompB_RickettsiaCMCMicro2_JN209      --------------------------------------------------


ompB_SL154_MZ970569                 --------------------------------------------------
ompB_Rsiciliensis_HM014441.1        --------------------------------------------------
ompB_Tenjiku-01_NZ_BCMR0100000      CGTACTAACAATGCAGAAAACGTAGTTACTAATGATATCGCAAATAGTCC
ompB_RickettsiaCMCMicro2_JN209      --------------------------------------------------


ompB_SL154_MZ970569                 --------------------------------------------------
ompB_Rsiciliensis_HM014441.1        --------------------------------------------------
ompB_Tenjiku-01_NZ_BCMR0100000      GTTTGGAGGTGCACCGGGTGTAGGTCAGAACGTTACAACATTTGTAAATG
ompB_RickettsiaCMCMicro2_JN209      --------------------------------------------------


ompB_SL154_MZ970569                 --------------------------------------------------
ompB_Rsiciliensis_HM014441.1        --------------------------------------------------
ompB_Tenjiku-01_NZ_BCMR0100000      CAACTAATACTGCAGCATATAATAATCTTCTTTTAGCTAAAAATAGTGCT
ompB_RickettsiaCMCMicro2_JN209      --------------------------------------------------
```

```
ompB_SL154_MZ970569                 --------------------------------------------------
ompB_Rsiciliensis_HM014441.1        --------------------------------------------------
ompB_Tenjiku-01_NZ_BCMR0100000      GATGCTGCTAATTTTGTTGGAGCTATCGTTACCGATACAAGTGCGGCCAT
ompB_RickettsiaCMCMicro2_JN209      --------------------------------------------------

ompB_SL154_MZ970569                 --------------------------------------------------
ompB_Rsiciliensis_HM014441.1        --------------------------------------------------
ompB_Tenjiku-01_NZ_BCMR0100000      AACTAATGCACAATTAGATGTAGCTAAAGATATCCAAGCTCAACTTGGTA
ompB_RickettsiaCMCMicro2_JN209      --------------------------------------------------

ompB_SL154_MZ970569                 --------------------------------------------------
ompB_Rsiciliensis_HM014441.1        --------------------------------------------------
ompB_Tenjiku-01_NZ_BCMR0100000      ACAGATTAGGTGCTCTTAGATATTTAGGTACTCCTGAAACTGCTGTAATG
ompB_RickettsiaCMCMicro2_JN209      --------------------------------------------------

ompB_SL154_MZ970569                 --------------------------------------------------
ompB_Rsiciliensis_HM014441.1        --------------------------------------------------
ompB_Tenjiku-01_NZ_BCMR0100000      GCTGGACCTGAAGCTGAAGCAATACCGGCTGCGGTTGCTGCAGGTGACGA
ompB_RickettsiaCMCMicro2_JN209      --------------------------------------------------

ompB_SL154_MZ970569                 --------------------------------------------------
ompB_Rsiciliensis_HM014441.1        --------------------------------------------------
ompB_Tenjiku-01_NZ_BCMR0100000      GGCTGTTGATAATGTAGCTTACGGTATATGGGCAAAACCTTTCTATACTG
ompB_RickettsiaCMCMicro2_JN209      --------------------------------------------------

ompB_SL154_MZ970569                 --------------------------------------------------
ompB_Rsiciliensis_HM014441.1        --------------------------------------------------
ompB_Tenjiku-01_NZ_BCMR0100000      ATGCACACCAAAGTAAGCAAGGTGGTTTAGCTGGTTATAAAGCTAAAACC
ompB_RickettsiaCMCMicro2_JN209      --------------------------------------------------

ompB_SL154_MZ970569                 --------------------------------------------------
ompB_Rsiciliensis_HM014441.1        --------------------------------------------------
ompB_Tenjiku-01_NZ_BCMR0100000      ACCGGTGTCGTAATCGGTTTAGATACGCTAGCTAACGATAACTTAATGAT
ompB_RickettsiaCMCMicro2_JN209      --------------------------------------------------

ompB_SL154_MZ970569                 --------------------------------------------------
ompB_Rsiciliensis_HM014441.1        --------------------------------------------------
ompB_Tenjiku-01_NZ_BCMR0100000      TGGTGCTGCTATAGGTATCACTAAAACTGATATAAAACACCAAGATTATA
ompB_RickettsiaCMCMicro2_JN209      --------------------------------------------------

ompB_SL154_MZ970569                 --------------------------------------------------
ompB_Rsiciliensis_HM014441.1        --------------------------------------------------
ompB_Tenjiku-01_NZ_BCMR0100000      AGAAAGGTGATAAAACCGACGTTAACGGTTTCTCATTCTCTCTATATGGT
ompB_RickettsiaCMCMicro2_JN209      --------------------------------------------------

ompB_SL154_MZ970569                 --------------------------------------------------
ompB_Rsiciliensis_HM014441.1        --------------------------------------------------
ompB_Tenjiku-01_NZ_BCMR0100000      GCCCAGCAGCTTGTTAAGAACTTCTTTGCTCAAGGTAGTGCAATATTTAG
ompB_RickettsiaCMCMicro2_JN209      --------------------------------------------------

ompB_SL154_MZ970569                 --------------------------------------------------
ompB_Rsiciliensis_HM014441.1        --------------------------------------------------
ompB_Tenjiku-01_NZ_BCMR0100000      CTTAAACCAAGTGAAGAACAAAAGTCAGCGTTACTTCTTCGATGCTAACG
```

```
ompB_RickettsiaCMCMicro2_JN209      --------------------------------------------------

ompB_SL154_MZ970569                 --------------------------------------------------
ompB_Rsiciliensis_HM014441.1        --------------------------------------------------
ompB_Tenjiku-01_NZ_BCMR0100000      GTAATATGAGCAAGCAAATTGCTGCCGGTCATTACGATAACATGACATTC
ompB_RickettsiaCMCMicro2_JN209      --------------------------------------------------

ompB_SL154_MZ970569                 --------------------------------------------------
ompB_Rsiciliensis_HM014441.1        --------------------------------------------------
ompB_Tenjiku-01_NZ_BCMR0100000      GGTGGTAACTTAACAGTCGGTTATGATTACAATGCAATGCAAGGTGTGTT
ompB_RickettsiaCMCMicro2_JN209      --------------------------------------------------

ompB_SL154_MZ970569                 --------------------------------------------------
ompB_Rsiciliensis_HM014441.1        --------------------------------------------------
ompB_Tenjiku-01_NZ_BCMR0100000      AGTAACTCCAATGGTAGGACTTAGCTACTTAAAGTCTTCTGACGAAAACT
ompB_RickettsiaCMCMicro2_JN209      ----------------GGACTTAGCTACTTAAAGTCTTCTGACGAAAACT
                                                    **********************************

ompB_SL154_MZ970569                 --------------------------------------------------
ompB_Rsiciliensis_HM014441.1        --------------------------------------------------
ompB_Tenjiku-01_NZ_BCMR0100000      ACAAAGAAACCGGTGCAACAGTTGCAAACAAGCAAGTTAACAGCAAGTTT
ompB_RickettsiaCMCMicro2_JN209      ACAAAGAAACCGGTGCAACAGTTGCAAACAAGCAAGTTAACAGCAAGTTT
                                    **************************************************

ompB_SL154_MZ970569                 --------------------------------------------------
ompB_Rsiciliensis_HM014441.1        --------------------------------------------------
ompB_Tenjiku-01_NZ_BCMR0100000      AGCGATAGAACCGATTTAATAGTAGGTGCTAAAGTAGCCGGCAGTACTAT
ompB_RickettsiaCMCMicro2_JN209      AGCGATAGAACCGATTTAATAGTAGGTGCTAAAGTAGCCGGCAGTACTAT
                                    **************************************************

ompB_SL154_MZ970569                 --------------------------------------------------
ompB_Rsiciliensis_HM014441.1        --------------------------------------------------
ompB_Tenjiku-01_NZ_BCMR0100000      GAACATAACTGATCTTGCGGTATATCCGGAAGTTCACGCTTTTGTGGTTC
ompB_RickettsiaCMCMicro2_JN209      GAACATAACTGATCTTGCGGTATATCCGGAAGTTCCCGCTTTTGTGGTTC
                                    *********************************** **************

ompB_SL154_MZ970569                 --------------------------------------------------
ompB_Rsiciliensis_HM014441.1        --------------------------------------------------
ompB_Tenjiku-01_NZ_BCMR0100000      ACAAAGTAATCGGTAGATTATCTAAAACTCAGTCTGTATTAGACGGACAA
ompB_RickettsiaCMCMicro2_JN209      ACAAAGTAATCGGTAGATTATCTAAAACTCAGTCTGTATTAGACGGACAA
                                    **************************************************

ompB_SL154_MZ970569                 --------------------------------------------------
ompB_Rsiciliensis_HM014441.1        --------------------------------------------------
ompB_Tenjiku-01_NZ_BCMR0100000      GTTACTCCGTGTATCAGCCAGCCTGACAGAACCGCTAAAACATCTTATAA
ompB_RickettsiaCMCMicro2_JN209      GTTACTCCGTGTA-------------------------------------
                                    *************

ompB_SL154_MZ970569                 --------------------------------------------------
ompB_Rsiciliensis_HM014441.1        --------------------------------------------------
ompB_Tenjiku-01_NZ_BCMR0100000      TTTAGGTTTAAGTGCAAGCATAAGATCTGATGCTAAGATGGAGTACGGAA
ompB_RickettsiaCMCMicro2_JN209      --------------------------------------------------

ompB_SL154_MZ970569                 --------------------------------------------------
ompB_Rsiciliensis_HM014441.1        --------------------------------------------------
```

```
ompB_Tenjiku-01_NZ_BCMR0100000      TCGGTTACGATGCTCAGATTTCAAGTAAATATATTGCACATCAAGGTACT
ompB_RickettsiaCMCMicro2_JN209      --------------------------------------------------


ompB_SL154_MZ970569                 ------------------------
ompB_Rsiciliensis_HM014441.1        ------------------------
ompB_Tenjiku-01_NZ_BCMR0100000      CTAAAAGTCCGTGTAAACTTCTAA
ompB_RickettsiaCMCMicro2_JN209      ------------------------
```

## ***glt*A alignment**

gltA_Tenjiku01_LC089864.1 sequence that matches
gltA_Tenjiku01_NZ_BCMR01000005.1:5548-6855 (100%) is underlined

```
gltA_NZ_BCMR01000005.1_5548-68      ATGACCAATGAAAATAATAATGATTCAGAATTTGCTGAATTAAAAATCAG
gltA_cmc08_GQ260637.1               --------------------------------------------------
gltA_SL154_MZ970598                 --------------------------------------------------
gltA_Rsiciliensis_HM014438.1        --------------------------------------------------


gltA_NZ_BCMR01000005.1_5548-68      AGGAAAAATATTTAAATTACCTATACTTAAAGCAAGTATCGGTGAGGATG
gltA_cmc08_GQ260637.1               --------------------------------------------------
gltA_SL154_MZ970598                 --------------------------------------------------
gltA_Rsiciliensis_HM014438.1        --------------------------------------------------


gltA_NZ_BCMR01000005.1_5548-68      TAATCGATATAAGTAGGGTATCTGCGGAAGCCGATTGTTTTACTTACGAC
gltA_cmc08_GQ260637.1               --------------------------------------------------
gltA_SL154_MZ970598                 --------------------------------------------------
gltA_Rsiciliensis_HM014438.1        --------------------------------------------------


gltA_NZ_BCMR01000005.1_5548-68      CCGGGTTTTATGTCTACTGCTTCTTGTCAGTCTACTATCACCTATATAGA
gltA_cmc08_GQ260637.1               --------------------------------------------------
gltA_SL154_MZ970598                 --------------------------------------------------
gltA_Rsiciliensis_HM014438.1        --------------------------------------------------


gltA_NZ_BCMR01000005.1_5548-68      CGGTGATAAAGGAATCTTGCGGCATCGAGGATATGATATTAAAGACTTAG
gltA_cmc08_GQ260637.1               --------------------------------------------------
gltA_SL154_MZ970598                 --------------------------------------------------
gltA_Rsiciliensis_HM014438.1        --------------------------------------------------


gltA_NZ_BCMR01000005.1_5548-68      CTGAGAAAAGTGATTTTTTAGAAGTGGCATATTTACTGATTTATGGGGAA
gltA_cmc08_GQ260637.1               --------------------------------------------------
gltA_SL154_MZ970598                 --------------------------------------------------
gltA_Rsiciliensis_HM014438.1        --------------------------------------------------


gltA_NZ_BCMR01000005.1_5548-68      CTACCAAGTGGCGAGCAGTATAATAATTTCACTAAACAGGTTGCTCATCA
gltA_cmc08_GQ260637.1               --------------------------------------------------
gltA_SL154_MZ970598                 --------------------------------------------------
gltA_Rsiciliensis_HM014438.1        --------------------------------------------------


gltA_NZ_BCMR01000005.1_5548-68      TTCATTAGTGAATGAAAGATTACACTATTTATTTCAAACCTTTTGTAGCT
gltA_cmc08_GQ260637.1               --------------------------------------------------
gltA_SL154_MZ970598                 --------------------------------------------------
gltA_Rsiciliensis_HM014438.1        --------------------------------------------------
```

```
gltA_NZ_BCMR01000005.1_5548-68      CTTCTCATCCTATGGCTATTATGCTTGCGGCTGTCGGTTCTCTTTCGGCA
gltA_cmc08_GQ260637.1               --------------------------------------------------
gltA_SL154_MZ970598                 --------------------------------------------------
gltA_Rsiciliensis_HM014438.1        --------------------------------------------------

gltA_NZ_BCMR01000005.1_5548-68      TTTTATCCTGATTTATTGAATTTTAAGGAAGCAGATTACGAACTTACCGC
gltA_cmc08_GQ260637.1               --------------------------------------------------
gltA_SL154_MZ970598                 --------------------------------------------------
gltA_Rsiciliensis_HM014438.1        --------------------------------------------------

gltA_NZ_BCMR01000005.1_5548-68      TATTAGAATGATTGCTAAGATACCTACCATCGCTGCAATGTCTTATAAAT
gltA_cmc08_GQ260637.1               --------------------------------------------------
gltA_SL154_MZ970598                 --------------------------------------------------
gltA_Rsiciliensis_HM014438.1        --------------------------------------------------

gltA_NZ_BCMR01000005.1_5548-68      ATTCTATAGGACAACCGTTTATTTATCCTGATAATTCGTTAGATTTTACC
gltA_cmc08_GQ260637.1               --------------------------------------------------
gltA_SL154_MZ970598                 --------------------------------------------------
gltA_Rsiciliensis_HM014438.1        --------------------------------------------------

gltA_NZ_BCMR01000005.1_5548-68      GAAAATTTTCTGCATATGATGTTTGCAACGCCTTGTATGAAATATACAGT
gltA_cmc08_GQ260637.1               --------------------------------------------------
gltA_SL154_MZ970598                 --------------------------------------------------
gltA_Rsiciliensis_HM014438.1        --------------------------------------------------

gltA_NZ_BCMR01000005.1_5548-68      AAATCCAATAATAAAAAATGCTCTTAATAAGATATTTATCCTACATGCCG
gltA_cmc08_GQ260637.1               --------------------------------------------------
gltA_SL154_MZ970598                 --------------------------------------------------
gltA_Rsiciliensis_HM014438.1        --------------------------------------------------

gltA_NZ_BCMR01000005.1_5548-68      ATCATGAGCAGAATGCTTCTACTTCAACAGTCCGAATTGCCGGCTCATCC
gltA_cmc08_GQ260637.1               --------------------------------------------------
gltA_SL154_MZ970598                 --------------------------------------------------
gltA_Rsiciliensis_HM014438.1        --------------------------------------------------

gltA_NZ_BCMR01000005.1_5548-68      GGAGCTAACCCTTTTGCTTGTATTAGCACGGGTATTGCCTCACTTTGGGG
gltA_cmc08_GQ260637.1               --------------------------------------------------
gltA_SL154_MZ970598                 --------------------------------------------------
gltA_Rsiciliensis_HM014438.1        --------------------------------------------------

gltA_NZ_BCMR01000005.1_5548-68      ACCTGCTCACGGCGGGGCTAATGAAGCGGTAATAAATATGCTTAAAGAAA
gltA_cmc08_GQ260637.1               -----------------------AAGCAGTGATAAATATGCTTAAAGAAA
gltA_SL154_MZ970598                 ---------------GGCTAATGAAGCGGTAATAAATATGCTTAAAGAAA
gltA_Rsiciliensis_HM014438.1        ---------------GGCTAATGAAGCGGTAATAAATATGCTTAAAGAAA
                                                   ************ ** *******************

gltA_NZ_BCMR01000005.1_5548-68      TCGGTAGTTCAGAGTATATTCCTAAATATATAGCTAAAGCTAAGGATAAA
gltA_cmc08_GQ260637.1               TCGGTAGTTCAGAGTATATTCCTAAATATATAGCTAAAGCTAAGGATAAA
gltA_SL154_MZ970598                 TCGGTAGTTCAGAGTATATTCCTAAATATATAGCTAAAGCTAAGGATAAA
gltA_Rsiciliensis_HM014438.1        TCGGTAGTTCAGAGTATATTCCTAAATATATAGCTAAAGCTAAGGATAAA
                                    **************************************************

gltA_NZ_BCMR01000005.1_5548-68      AATGATCCATTTAGATTAATGGGTTTTGGTCATCGTGTATATAAAAACTA
gltA_cmc08_GQ260637.1               AATGATCCATTTAGATTAATGGGTTTTGGTCATCGTGTATATAAAAACTA
```

```
gltA_SL154_MZ970598                 AATGATCCATTTAGATTAATGGGTTTTGGTCATCGTGTATATAAAAACTA
gltA_Rsiciliensis_HM014438.1        AATGATCCATTTAGATTAATGGGTTTTGGTCATCGTGTATATAAAAACTA
                                    **************************************************

gltA_NZ_BCMR01000005.1_5548-68      TGACCCGCGTGCCGCAGTACTTAAAGAAACGTGCAAAGAAGTATTAAAGG
gltA_cmc08_GQ260637.1               TGACCCGCGTGCCGCAGTACTTAAAGAAACGTGCAAAGAAGTATTAAAGG
gltA_SL154_MZ970598                 TGACCCGCGTGCCGCAGTACTTAAAGAAACGTGCAAAGAAGTATTAAAGG
gltA_Rsiciliensis_HM014438.1        TGACCCGCGTGCCGCAGTACTTAAAGAAACGTGCAAAGAAGTATTAAAGG
                                    **************************************************

gltA_NZ_BCMR01000005.1_5548-68      AACTCGGGCAGCTAGACAACAATCCGCTCTTACAAATAGCAATAGAACTT
gltA_cmc08_GQ260637.1               AACTCGGGCAGCTAGACAACAATCCGCTCTTACAAATAGCAATAGAACTT
gltA_SL154_MZ970598                 AACTCGGGCAGCTAGACAACAATCCGCTCTTACAAATAGCAATAGAACTT
gltA_Rsiciliensis_HM014438.1        AACTCGGGCAGCTAGACAACAATCCGCTCTTACAAATAGCAATAGAACTT
                                    **************************************************

gltA_NZ_BCMR01000005.1_5548-68      GAAGCTATCGCTCTTAAAGATGAATATTTTATTGAGAGAAAATTATATCC
gltA_cmc08_GQ260637.1               GAAGCTATCGCTCTTAAAGATGAATATTTTATTGAGAGAAAATTATATCC
gltA_SL154_MZ970598                 GAAGCTATCGCTCTTAAAGATGAATATTTTATTGAGAGAAAATTATATCC
gltA_Rsiciliensis_HM014438.1        GAAGCTATCGCTCTTAAAGATGAATATTTTATTGAGAGAAAATTATATCC
                                    **************************************************

gltA_NZ_BCMR01000005.1_5548-68      AAATGTTGATTTTTATTCGGGTATTATCTATAAAGCTATGGGTATACCGT
gltA_cmc08_GQ260637.1               AAATGTTGATTTTTATTCGGGTATTATCTATAAAGCTATGGGTATACCGT
gltA_SL154_MZ970598                 AAATGTTGATTTTTATTCGGGTATTATCTATAAAGCTAGGGGTATACCGT
gltA_Rsiciliensis_HM014438.1        AAATGTTGATTTTTATTCGGGTATTATCTATAAAGCTA------------
                                    **************************************

gltA_NZ_BCMR01000005.1_5548-68      CGCAAATGTTCACGGTACTTTTTGCAATAGCAAGAACCGTAGGCTGGATG
gltA_cmc08_GQ260637.1               CGCA----------------------------------------------
gltA_SL154_MZ970598                 CGCAAA--------------------------------------------
gltA_Rsiciliensis_HM014438.1        --------------------------------------------------
                                    ******

gltA_NZ_BCMR01000005.1_5548-68      GCACAATGGAAAGAAATGCACGAAGACCCTGAACAAAAAATCAGCAGACC
gltA_cmc08_GQ260637.1               --------------------------------------------------
gltA_SL154_MZ970598                 --------------------------------------------------
gltA_Rsiciliensis_HM014438.1        --------------------------------------------------


gltA_NZ_BCMR01000005.1_5548-68      TAGACAGCTTTACACCGGTTATGTACATAGAGAGTATAAGGGTATTCGGG
gltA_cmc08_GQ260637.1               --------------------------------------------------
gltA_SL154_MZ970598                 --------------------------------------------------
gltA_Rsiciliensis_HM014438.1        --------------------------------------------------


gltA_NZ_BCMR01000005.1_5548-68      AGAGGTAA
gltA_cmc08_GQ260637.1               --------
gltA_SL154_MZ970598                 --------
gltA_Rsiciliensis_HM014438.1        --------
```

## ***sca4* alignment**

No sca4_SL154 or CMCMICRO_2 sequences obtained

Two Tenjiku01 sca4 sequences identical except for length:
sca4_NZ_BCMR01000008.1:35645-38707 Rickettsia sp. Tenjiku01,
>sca4_Tenjiku01_LC089863.1

```
sca4_Tenjiku01_LC089863.1           ------------------------------------------------AA
sca4_NZ_BCMR01000008.1_35645-3      ATGAGTAAAGACGGTAACCTAGATACAAGTGAATTTGATCCTTTAGCAAA
```

```
sca4_Rsiciliensis_HM014440.1        --------------------------------------------------
                                                                                  **

sca4_Tenjiku01_LC089863.1           TAAGGAATATACAGAAGCACAAAAGCAAACATTAGAACAAGAGCAAAAAG
sca4_NZ_BCMR01000008.1_35645-3      TAAGGAATATACAGAAGCACAAAAGCAAACATTAGAACAAGAGCAAAAAG
sca4_Rsiciliensis_HM014440.1        --------------------------------------------------
                                    **************************************************

sca4_Tenjiku01_LC089863.1           AATTTTTATCTCAAACTACAACCCCAGAACTAGAAGCTGACGATGGTTTT
sca4_NZ_BCMR01000008.1_35645-3      AATTTTTATCTCAAACTACAACCCCAGAACTAGAAGCTGACGATGGTTTT
sca4_Rsiciliensis_HM014440.1        --------------------------------------------------
                                    **************************************************

sca4_Tenjiku01_LC089863.1           ATCGTTACTTCTGCATCTTCTGCTCAATCTACCCCTTCAATTAGTGCTTT
sca4_NZ_BCMR01000008.1_35645-3      ATCGTTACTTCTGCATCTTCTGCTCAATCTACCCCTTCAATTAGTGCTTT
sca4_Rsiciliensis_HM014440.1        --------------ATCTTCTGCTCAATCTATCCCTTCAATTAGTGCTTT
                                    ******************************* ******************

sca4_Tenjiku01_LC089863.1           ATCGGACAATATCTCTCCTGACAGTCAGACATCAGACCCAATAACCAAGG
sca4_NZ_BCMR01000008.1_35645-3      ATCGGACAATATCTCTCCTGACAGTCAGACATCAGACCCAATAACCAAGG
sca4_Rsiciliensis_HM014440.1        ATCGGACAATATCTCTCCTGACAGTCAGACATCAGACCCAATAACCAAGG
                                    **************************************************

sca4_Tenjiku01_LC089863.1           CTGTAAGAGAAACAATTATACAACCGCAAAAAGATAATTTAATAGAACAA
sca4_NZ_BCMR01000008.1_35645-3      CTGTAAGAGAAACAATTATACAACCGCAAAAAGATAATTTAATAGAACAA
sca4_Rsiciliensis_HM014440.1        CTGTAAGAGAAACAATTATACAACCGCAAAAAGATAATTTAATAGAACAA
                                    **************************************************

sca4_Tenjiku01_LC089863.1           ATATTAAAAGACCTGGCAGCCCTTACAGACCGTGATTTAGCTGAACAAAA
sca4_NZ_BCMR01000008.1_35645-3      ATATTAAAAGACCTGGCAGCCCTTACAGACCGTGATTTAGCTGAACAAAA
sca4_Rsiciliensis_HM014440.1        ATATTAAAAGACCTTGCAGCCCTTACAGACCGTGATTTAGCTGAACAAAA
                                    ************** ***********************************

sca4_Tenjiku01_LC089863.1           AAGAAAAGAAATAGAAGAAGAAAAAGATAAAACATTAAGTACTTTTTTCG
sca4_NZ_BCMR01000008.1_35645-3      AAGAAAAGAAATAGAAGAAGAAAAAGATAAAACATTAAGTACTTTTTTCG
sca4_Rsiciliensis_HM014440.1        AAGAAAAGAAATAGAAGAAGAAAAAGCTAAAACATTAAGTACTTTTTTCG
                                    ************************** ***********************

sca4_Tenjiku01_LC089863.1           GTAATCCGGCTAATAGAGCATTTATTGATAAGGCTTTAGAAAATCCTGAG
sca4_NZ_BCMR01000008.1_35645-3      GTAATCCGGCTAATAGAGCATTTATTGATAAGGCTTTAGAAAATCCTGAG
sca4_Rsiciliensis_HM014440.1        GTAATCCGGCTAATAGAGCATTTATTGATAAGGCTTTAGAAAATCCTGAG
                                    **************************************************

sca4_Tenjiku01_LC089863.1           CTTAAAAAGAAATTAGAATCAATAGAAATAGCCGGCTATAAAAATGTGCA
sca4_NZ_BCMR01000008.1_35645-3      CTTAAAAAGAAATTAGAATCAATAGAAATAGCCGGCTATAAAAATGTGCA
sca4_Rsiciliensis_HM014440.1        CTTAAAAAGAAATTAGAATCAATAGAAATAGCCGGCTATAAAAATGTGCA
                                    **************************************************

sca4_Tenjiku01_LC089863.1           TAATACATTTAGTGCCGCTAGTGGATACCCTGGTGGATTTAAACCGGTAC
sca4_NZ_BCMR01000008.1_35645-3      TAATACATTTAGTGCCGCTAGTGGATACCCTGGTGGATTTAAACCGGTAC
sca4_Rsiciliensis_HM014440.1        TAATACATTTAGTGCCGCTAGTGGCTACCCTGGTGGATTTAAACCGGTAC
                                    ************************ *************************

sca4_Tenjiku01_LC089863.1           AGTGGGAAAATCACGTAAGTGCAAGCGATCTTAGAGCAACAGTAGTTAAA
sca4_NZ_BCMR01000008.1_35645-3      AGTGGGAAAATCACGTAAGTGCAAGCGATCTTAGAGCAACAGTAGTTAAA
sca4_Rsiciliensis_HM014440.1        AGTGGGAAAATCACGTAAGTGCAAGCGATCTTAGAGCAACAGTAGTTAAA
                                    **************************************************

sca4_Tenjiku01_LC089863.1           AATGATGCAGGTGATGAACTCTGTACCTTAAATGAAACAACTGTTAAAAC
sca4_NZ_BCMR01000008.1_35645-3      AATGATGCAGGTGATGAACTCTGTACCTTAAATGAAACAACTGTTAAAAC
sca4_Rsiciliensis_HM014440.1        AATGATGCAGGTGATGAACTCTGTACCTTAAATGAAACAACTGTTAAAAC
                                    **************************************************
```

```
sca4_Tenjiku01_LC089863.1         TAAGCCTTTTACTTTAGCTAAACAAGACGGTACTCAGGTTCAGATCAGCT
sca4_NZ_BCMR01000008.1_35645-3    TAAGCCTTTTACTTTAGCTAAACAAGACGGTACTCAGGTTCAGATCAGCT
sca4_Rsiciliensis_HM014440.1      TAAGCCTTTTACTTTAGCTAAACAAGACGGTACTCAGGTTCAGATCAGCT
                                  **************************************************

sca4_Tenjiku01_LC089863.1         CATATAGGGAAATAGATTTTCCTATAAAACTTGATAAAGCCGATGGGTCA
sca4_NZ_BCMR01000008.1_35645-3    CATATAGGGAAATAGATTTTCCTATAAAACTTGATAAAGCCGATGGGTCA
sca4_Rsiciliensis_HM014440.1      CATATAGGGAAATAGATTTTCCTATAAAACTTGATAAAGCCGATGGGTCA
                                  **************************************************

sca4_Tenjiku01_LC089863.1         ATGCATTTATCGATGGTAGCATTAAAAGCTGATGGCACAAAGCCCTTCAA
sca4_NZ_BCMR01000008.1_35645-3    ATGCATTTATCGATGGTAGCATTAAAAGCTGATGGCACAAAGCCCTTCAA
sca4_Rsiciliensis_HM014440.1      ATGCATTTATCGATGGTAGCATTAAAAGCTGATGGCACAAAGCCCTCCAA
                                  ********************************************** ***

sca4_Tenjiku01_LC089863.1         AGATAAAGCCGTATATTTCACTGCCCACTACGAAGAAGGACCAAATGGTA
sca4_NZ_BCMR01000008.1_35645-3    AGATAAAGCCGTATATTTCACTGCCCACTACGAAGAAGGACCAAATGGTA
sca4_Rsiciliensis_HM014440.1      AGATAAAGCCGTATATTTCACTGCCCACTACGAAGAAGGACCAAACGGTA
                                  ********************************************* ****

sca4_Tenjiku01_LC089863.1         AACCTCAACTTAAAGAAATAAGCTCACCAAAACCTTTAAAATTTGCCGGA
sca4_NZ_BCMR01000008.1_35645-3    AACCTCAACTTAAAGAAATAAGCTCACCAAAACCTTTAAAATTTGCCGGA
sca4_Rsiciliensis_HM014440.1      AACCTCAACTTAAAGAAATAAGCTC-------------------------
                                  **************************************************

sca4_Tenjiku01_LC089863.1         ACCGGAGATGACGCAATAGCTTATATAGAGCATGGTGGAGAAATTTATAC
sca4_NZ_BCMR01000008.1_35645-3    ACCGGAGATGACGCAATAGCTTATATAGAGCATGGTGGAGAAATTTATAC
sca4_Rsiciliensis_HM014440.1      --------------------------------------------------
                                  **************************************************

sca4_Tenjiku01_LC089863.1         ACTTGCGGTAACACGCGGTAAATATAAAGAAATGATGAAGGAGGTAGAAC
sca4_NZ_BCMR01000008.1_35645-3    ACTTGCGGTAACACGCGGTAAATATAAAGAAATGATGAAGGAGGTAGAAC
sca4_Rsiciliensis_HM014440.1      --------------------------------------------------
                                  **************************************************

sca4_Tenjiku01_LC089863.1         TAAATCAAGGGCAGAGCGTTGATTTATCGCAAGCTGAAGATATTATAATA
sca4_NZ_BCMR01000008.1_35645-3    TAAATCAAGGGCAGAGCGTTGATTTATCGCAAGCTGAAGATATTATAATA
sca4_Rsiciliensis_HM014440.1      --------------------------------------------------
                                  **************************************************

sca4_Tenjiku01_LC089863.1         GGACAAGGACAAAGTAAGGAACAACCGCTAATAACTCCACAGCAAACAAC
sca4_NZ_BCMR01000008.1_35645-3    GGACAAGGACAAAGTAAGGAACAACCGCTAATAACTCCACAGCAAACAAC
sca4_Rsiciliensis_HM014440.1      --------------------------------------------------
                                  **************************************************

sca4_Tenjiku01_LC089863.1         AAGTTCATCGGTTGAACCACCTCAGTATAAACAACAAGTACCACCAATTA
sca4_NZ_BCMR01000008.1_35645-3    AAGTTCATCGGTTGAACCACCTCAGTATAAACAACAAGTACCACCAATTA
sca4_Rsiciliensis_HM014440.1      --------------------------------------------------
                                  **************************************************

sca4_Tenjiku01_LC089863.1         CTCCTACTAACCAACCACTGCAACCTGAGACTTCACAAATGCCACAGTCG
sca4_NZ_BCMR01000008.1_35645-3    CTCCTACTAACCAACCACTGCAACCTGAGACTTCACAAATGCCACAGTCG
sca4_Rsiciliensis_HM014440.1      --------------------------------------------------
                                  **************************************************

sca4_Tenjiku01_LC089863.1         CAACAAGTGAATCCAAATCTTCTTAATGCAGCTACGGCTTTATCAGGCAG
sca4_NZ_BCMR01000008.1_35645-3    CAACAAGTGAATCCAAATCTTCTTAATGCAGCTACGGCTTTATCAGGCAG
sca4_Rsiciliensis_HM014440.1      --------------------------------------------------
                                  **************************************************

sca4_Tenjiku01_LC089863.1         CATGCAAGATTTATTAAATTATGTAAATACAGGTTTAACAAAAGAAATTG
sca4_NZ_BCMR01000008.1_35645-3    CATGCAAGATTTATTAAATTATGTAAATACAGGTTTAACAAAAGAAATTG
sca4_Rsiciliensis_HM014440.1      --------------------------------------------------
```

```
                                    **************************************************

sca4_Tenjiku01_LC089863.1           ATAGCAATAAACAAATTGATTTAATTAAAGAAGCAGCCACGGCAATTCTT
sca4_NZ_BCMR01000008.1_35645-3      ATAGCAATAAACAAATTGATTTAATTAAAGAAGCAGCCACGGCAATTCTT
sca4_Rsiciliensis_HM014440.1        --------------------------------------------------
                                    **************************************************

sca4_Tenjiku01_LC089863.1           AATAATGAGAAAAGTGATATTGCGGAAAAGCAGGCTAATATCATTGCTTT
sca4_NZ_BCMR01000008.1_35645-3      AATAATGAGAAAAGTGATATTGCGGAAAAGCAGGCTAATATCATTGCTTT
sca4_Rsiciliensis_HM014440.1        --------------------------------------------------
                                    **************************************************

sca4_Tenjiku01_LC089863.1           AGCTGAAAATACGGTCAATAATAAAAACCTCAAACCGGATGCAAAAGTAG
sca4_NZ_BCMR01000008.1_35645-3      AGCTGAAAATACGGTCAATAATAAAAACCTCAAACCGGATGCAAAAGTAG
sca4_Rsiciliensis_HM014440.1        --------------------------------------------------
                                    **************************************************

sca4_Tenjiku01_LC089863.1           CTGGAGTCAATGCAGTATTAGAAACCATAAAAAATGATCAGAATACCCCA
sca4_NZ_BCMR01000008.1_35645-3      CTGGAGTCAATGCAGTATTAGAAACCATAAAAAATGATCAGAATACCCCA
sca4_Rsiciliensis_HM014440.1        --------------------------------------------------
                                    **************************************************

sca4_Tenjiku01_LC089863.1           GACCTAGAAAAATCAAAAATGCTTGAAGCTACAGTAGCTATCGTTTTAAA
sca4_NZ_BCMR01000008.1_35645-3      GACCTAGAAAAATCAAAAATGCTTGAAGCTACAGTAGCTATCGTTTTAAA
sca4_Rsiciliensis_HM014440.1        --------------------------------------------------
                                    **************************************************

sca4_Tenjiku01_LC089863.1           TTCAGAGAATCTTGAGCCGAAGCAAAAACAGCAGATGTTAGAAAAGGCAG
sca4_NZ_BCMR01000008.1_35645-3      TTCAGAGAATCTTGAGCCGAAGCAAAAACAGCAGATGTTAGAAAAGGCAG
sca4_Rsiciliensis_HM014440.1        --------------------------------------------------
                                    **************************************************

sca4_Tenjiku01_LC089863.1           TAGACGTCGGTTTAAGTCTTAAAGATGATGCAAGTAGAGCTGCAACAATT
sca4_NZ_BCMR01000008.1_35645-3      TAGACGTCGGTTTAAGTCTTAAAGATGATGCAAGTAGAGCTGCAACAATT
sca4_Rsiciliensis_HM014440.1        --------------------------------------------------
                                    **************************************************

sca4_Tenjiku01_LC089863.1           GATGGTATTAAGGATATTGTAATAACAAGTAACCTTTCTACTGAAGATAC
sca4_NZ_BCMR01000008.1_35645-3      GATGGTATTAAGGATATTGTAATAACAAGTAACCTTTCTACTGAAGATAC
sca4_Rsiciliensis_HM014440.1        --------------------------------------------------
                                    **************************************************

sca4_Tenjiku01_LC089863.1           AATGCTTATAGCAGTAGGTGATAAGGTTAATGTCTCTGAATTAAGCAATG
sca4_NZ_BCMR01000008.1_35645-3      AATGCTTATAGCAGTAGGTGATAAGGTTAATGTCTCTGAATTAAGCAATG
sca4_Rsiciliensis_HM014440.1        --------------------------------------------------
                                    **************************************************

sca4_Tenjiku01_LC089863.1           CGGCAAAACAAAAATTATTAGGTTCTGTATTAAAGAAAGGTGTAGAAGCC
sca4_NZ_BCMR01000008.1_35645-3      CGGCAAAACAAAAATTATTAGGTTCTGTATTAAAGAAAGGTGTAGAAGCC
sca4_Rsiciliensis_HM014440.1        --------------------------------------------------
                                    **************************************************

sca4_Tenjiku01_LC089863.1           CAAGTTCTCAGTCTGGCACAACAACAATTGATGCAGCAGTATTTAGATAA
sca4_NZ_BCMR01000008.1_35645-3      CAAGTTCTCAGTCTGGCACAACAACAATTGATGCAGCAGTATTTAGATAA
sca4_Rsiciliensis_HM014440.1        --------------------------------------------------
                                    **************************************************

sca4_Tenjiku01_LC089863.1           GATTACGGCAGAACAAACTAAAAAAGATACAATAAAAAAAGTAAATGATA
sca4_NZ_BCMR01000008.1_35645-3      GATTACGGCAGAACAAACTAAAAAAGATACAATAAAAAAAGTAAATGATA
sca4_Rsiciliensis_HM014440.1        --------------------------------------------------
                                    **************************************************

sca4_Tenjiku01_LC089863.1           TTTTGTTTGATCCTCTAAGTAATACTGAATTAAAAACAACAAACATACAA
```

```
sca4_NZ_BCMR01000008.1_35645-3      TTTTGTTTGATCCTCTAAGTAATACTGAATTAAAAACAACAAACATACAA
sca4_Rsiciliensis_HM014440.1        --------------------------------------------------
                                    **************************************************

sca4_Tenjiku01_LC089863.1           GCCATTACGTCTAATGTTTTAGATGGTCCGGCTACAGCAGAAGTAAAAGG
sca4_NZ_BCMR01000008.1_35645-3      GCCATTACGTCTAATGTTTTAGATGGTCCGGCTACAGCAGAAGTAAAAGG
sca4_Rsiciliensis_HM014440.1        --------------------------------------------------
                                    **************************************************

sca4_Tenjiku01_LC089863.1           CGAAATAATTCAAGAAATTACTAACATAGTTGCAGGAAGCTCGCTTGAAG
sca4_NZ_BCMR01000008.1_35645-3      CGAAATAATTCAAGAAATTACTAACATAGTTGCAGGAAGCTCGCTTGAAG
sca4_Rsiciliensis_HM014440.1        --------------------------------------------------
                                    **************************************************

sca4_Tenjiku01_LC089863.1           CTCAAGACAAAGCAGCGATTGTTAAAGGGATAGGCGAGACTATAGCTACT
sca4_NZ_BCMR01000008.1_35645-3      CTCAAGACAAAGCAGCGATTGTTAAAGGGATAGGCGAGACTATAGCTACT
sca4_Rsiciliensis_HM014440.1        --------------------------------------------------
                                    **************************************************

sca4_Tenjiku01_LC089863.1           CATAGTGATACCTCTTTATCCTTACCTAATAAAGCACTTATTATGGCATC
sca4_NZ_BCMR01000008.1_35645-3      CATAGTGATACCTCTTTATCCTTACCTAATAAAGCACTTATTATGGCATC
sca4_Rsiciliensis_HM014440.1        --------------------------------------------------
                                    **************************************************

sca4_Tenjiku01_LC089863.1           AGCGGAAAAAGGTATTGCAGAAAGTCAGACCAATTTACCTGATAGAGAGC
sca4_NZ_BCMR01000008.1_35645-3      AGCGGAAAAAGGTATTGCAGAAAGTCAGACCAATTTACCTGATAGAGAGC
sca4_Rsiciliensis_HM014440.1        --------------------------------------------------
                                    **************************************************

sca4_Tenjiku01_LC089863.1           TAATGACTAAAGGGTTAGTAGACGGTATTTATGAAGGCAAAGGAGGTCCT
sca4_NZ_BCMR01000008.1_35645-3      TAATGACTAAAGGGTTAGTAGACGGTATTTATGAAGGCAAAGGAGGTCCT
sca4_Rsiciliensis_HM014440.1        --------------------------------------------------
                                    **************************************************

sca4_Tenjiku01_LC089863.1           GAAATAACTAAGGCAGTTTCTAGCGGGATCGATAATAGTAATATTAATGA
sca4_NZ_BCMR01000008.1_35645-3      GAAATAACTAAGGCAGTTTCTAGCGGGATCGATAATAGTAATATTAATGA
sca4_Rsiciliensis_HM014440.1        --------------------------------------------------
                                    **************************************************

sca4_Tenjiku01_LC089863.1           CTCTGAGAAAGAGGCACTTAAAAAAGCTAAAGATGCAGCGAGTGAGGCAG
sca4_NZ_BCMR01000008.1_35645-3      CTCTGAGAAAGAGGCACTTAAAAAAGCTAAAGATGCAGCGAGTGAGGCAG
sca4_Rsiciliensis_HM014440.1        --------------------------------------------------
                                    **************************************************

sca4_Tenjiku01_LC089863.1           CTTTAGATAGAGACACTCAAAATTTAACTGAAGGGTTAAAAGGACAGAAT
sca4_NZ_BCMR01000008.1_35645-3      CTTTAGATAGAGACACTCAAAATTTAACTGAAGGGTTAAAAGGACAGAAT
sca4_Rsiciliensis_HM014440.1        --------------------------------------------------
                                    **************************************************

sca4_Tenjiku01_LC089863.1           ATAGAAGAACACAAGCCTCACGATGATATATATAACAAAGCTCGAGAAGT
sca4_NZ_BCMR01000008.1_35645-3      ATAGAAGAACACAAGCCTCACGATGATATATATAACAAAGCTCGAGAAGT
sca4_Rsiciliensis_HM014440.1        --------------------------------------------------
                                    **************************************************

sca4_Tenjiku01_LC089863.1           AATTAACGCTGTTAATCCTGTTATAGAAGCATTAGAAAAATCTAAAGACC
sca4_NZ_BCMR01000008.1_35645-3      AATTAACGCTGTTAATCCTGTTATAGAAGCATTAGAAAAATCTAAAGACC
sca4_Rsiciliensis_HM014440.1        --------------------------------------------------
                                    **************************************************

sca4_Tenjiku01_LC089863.1           CGGTAGTGTCGGCAGAAGAAAGAATTGTACAAGAAACTTCTAGTATATTA
sca4_NZ_BCMR01000008.1_35645-3      CGGTAGTGTCGGCAGAAGAAAGAATTGTACAAGAAACTTCTAGTATATTA
sca4_Rsiciliensis_HM014440.1        --------------------------------------------------
                                    **************************************************
```

```
sca4_Tenjiku01_LC089863.1           AATAATATCTCTAAGCTTGCAGTTGAGAAAGTCAATAATTTTCGTACTAT
sca4_NZ_BCMR01000008.1_35645-3      AATAATATCTCTAAGCTTGCAGTTGAGAAAGTCAATAATTTTCGTACTAT
sca4_Rsiciliensis_HM014440.1        --------------------------------------------------
                                    **************************************************

sca4_Tenjiku01_LC089863.1           GCTTTCTCCAAATGGTAACCTTAAAACTCTTGAAGAAAAAAAAGAGGAAT
sca4_NZ_BCMR01000008.1_35645-3      GCTTTCTCCAAATGGTAACCTTAAAACTCTTGAAGAAAAAAAAGAGGAAT
sca4_Rsiciliensis_HM014440.1        --------------------------------------------------
                                    **************************************************

sca4_Tenjiku01_LC089863.1           CAATAAAAAAAGTAGATGAGCTGGTAAAGGCATTTGGTACTAAATCTTCT
sca4_NZ_BCMR01000008.1_35645-3      CAATAAAAAAAGTAGATGAGCTGGTAAAGGCATTTGGTACTAAATCTTCT
sca4_Rsiciliensis_HM014440.1        --------------------------------------------------
                                    **************************************************

sca4_Tenjiku01_LC089863.1           ACTGAAGAACAGCAAAGTTTCATTAAAGCTAATTTAATTGATGATAAAAC
sca4_NZ_BCMR01000008.1_35645-3      ACTGAAGAACAGCAAAGTTTCATTAAAGCTAATTTAATTGATGATAAAAC
sca4_Rsiciliensis_HM014440.1        --------------------------------------------------
                                    **************************************************

sca4_Tenjiku01_LC089863.1           TTTATCTAAAGAGGTACGTTTACAAGCTATAGATAAGTTATTACAAGAAC
sca4_NZ_BCMR01000008.1_35645-3      TTTATCTAAAGAGGTACGTTTACAAGCTATAGATAAGTTATTACAAGAAC
sca4_Rsiciliensis_HM014440.1        --------------------------------------------------
                                    **************************************************

sca4_Tenjiku01_LC089863.1           AAAAACGAGCAGAAGCAATTGAAAACCCTAGTGTTAAAACGGAAGATGTA
sca4_NZ_BCMR01000008.1_35645-3      AAAAACGAGCAGAAGCAATTGAAAACCCTAGTGTTAAAACGGAAGATGTA
sca4_Rsiciliensis_HM014440.1        --------------------------------------------------
                                    **************************************************

sca4_Tenjiku01_LC089863.1           AGGGTAGTATCAGGAAAGTCTAAATTAAAACCTATAAGTAAAGATAATCC
sca4_NZ_BCMR01000008.1_35645-3      AGGGTAGTATCAGGAAAGTCTAAATTAAAACCTATAAGTAAAGATAATCC
sca4_Rsiciliensis_HM014440.1        --------------------------------------------------
                                    **************************************************

sca4_Tenjiku01_LC089863.1           AGATATTGAAAAAGCTAAAATGGTAGTAGGAAGAGATAGAGTTAATATCA
sca4_NZ_BCMR01000008.1_35645-3      AGATATTGAAAAAGCTAAAATGGTAGTAGGAAGAGATAGAGTTAATATCA
sca4_Rsiciliensis_HM014440.1        --------------------------------------------------
                                    **************************************************

sca4_Tenjiku01_LC089863.1           AAGGGAATATAAAAATTATGGGAGCATTAATGAATGCAAGAGATATCATT
sca4_NZ_BCMR01000008.1_35645-3      AAGGGAATATAAAAATTATGGGAGCATTAATGAATGCAAGAGATATCATT
sca4_Rsiciliensis_HM014440.1        --------------------------------------------------
                                    **************************************************

sca4_Tenjiku01_LC089863.1           CAGTCGGAAAATTTAAATAAATCAACACCTATTAAAAGAGAGTCTTCCCC
sca4_NZ_BCMR01000008.1_35645-3      CAGTCGGAAAATTTAAATAAATCAACACCTATTAAAAGAGAGTCTTCCCC
sca4_Rsiciliensis_HM014440.1        --------------------------------------------------
                                    **************************************************

sca4_Tenjiku01_LC089863.1           TCCACAACGCTGAAA
sca4_NZ_BCMR01000008.1_35645-3      TCCACAACGCTGA--
sca4_Rsiciliensis_HM014440.1        ---------------
                                    *************
```

//////////////////////////////////////////////////////////////////////////

**Part B. SL94 sequence comparisons to CDC_RconJC480-related sequences**

## ***omp*A alignment**

```
ompA_SL94_MZ970588                  ---------------------TTATTTCAAAAAGCAATACAACAAGGTCT
```

```
ompA_RconJC480_MN548866.1         ---------------------TTATTTCAAAAAGCAATACAACAAGGTCT
ompA_Rslovaca13-B_U43808.1        ---------------------TTATTTCAAAAAGCAATACAACAAGGTCT
ompA_RslovacaCWPP-D_CP003375.1    ATGGCGAATATTTCTCCAAAATTATTTCAAAAAGCAATACAACAAGGTCT
ompA_Rhhaem_endosymb_KU895509.    --------------------------------------------------
ompA_RhhaemMeppadi_MF405464.2     ATGGCGAATATTTCTCCAAAATTATTTCAAAAAGCAATACAACAAGGTCT
                                  **************************************************

ompA_SL94_MZ970588                TAAAGCCGCTTTATTCACCACCTCAACCGCAGCGATAATGCTGAGTAGTA
ompA_RconJC480_MN548866.1         TAAAGCCGCTTTATTCACCACCTCAACCGCAGCGATAATGCTGAGTAGTA
ompA_Rslovaca13-B_U43808.1        TAAAGCCGCTTTATTCACCACCTCAACCGCAGCGATAATGCTGAGTAGTA
ompA_RslovacaCWPP-D_CP003375.1    TAAAGCCGCTTTATTCACCACCTCAACCGCAGCGATAATGCTGAGTAGTA
ompA_Rhhaem_endosymb_KU895509.    ----GCCGCTTTATTCACCACCTCAACCGCAGCGATAATGCTGAGTAGTA
ompA_RhhaemMeppadi_MF405464.2     TAAAGCTGCTTTATTCACCACCTCAACCGCAGCGATAATGCTGAGTAGTA
                                  ****** *******************************************

ompA_SL94_MZ970588                GCGGGGTACTCGGTGTTGCTGCAGGTGTTATTGCTACTAATAAT---GCA
ompA_RconJC480_MN548866.1         GCGGGGTACTCGGTGTTGCTGCAGGTGTTATTGCTACTAATAAT---GCA
ompA_Rslovaca13-B_U43808.1        GCGGGGCACTCGGTGTTGCTGCAGGTGTTATTGCTACTAATAATAATGCA
ompA_RslovacaCWPP-D_CP003375.1    GCGGGGCACTCGGTGTTGCTGCAGGTGTTATTGCTACTAATAATAATGCA
ompA_Rhhaem_endosymb_KU895509.    GCGGGGTACTCGGTGTTGCTGCAGGTGTTATTGCTACTAATAAT---GCA
ompA_RhhaemMeppadi_MF405464.2     GCGGGGCACTCGGTGTTGCTGTAGGTGTTATTTCTATTAATAATAATGCA
                                  ****** ************** ********** *** *******   ***

ompA_SL94_MZ970588                ACATTTAGTGATAATGTTGGCAATAATAATTGGAATGAGATAACGGCTGG
ompA_RconJC480_MN548866.1         ACATTTAGTGATAATGTTGGCAATAATAATTGGAATGAGATAACGGCTGA
ompA_Rslovaca13-B_U43808.1        GCATTTAGTGATAATGTTGGCAATAATAATTGGAATGAGATAACGGCTGG
ompA_RslovacaCWPP-D_CP003375.1    GCATTTAGTGATAATGTTGGCAATAATAATTGGAATGAGATAACGGCTGG
ompA_Rhhaem_endosymb_KU895509.    ACATTTAGTGATAATGTTGGCAATAATAATTGGAATGAGATAACGGCTGG
ompA_RhhaemMeppadi_MF405464.2     GCATTTAGTGATCTTGCTGTTGCCAATAATTGGAATGATATAACGGCTGA
                                   ***********  ** **     ************** ********** 

ompA_SL94_MZ970588                AGGGGTAGCTAATGGTACTCCTGCTGGCAGTCCTCAAAACAATTG---GG
ompA_RconJC480_MN548866.1         AGGGGTAGCTAATGGTACTCCTGCTGGCAGTCCTCAAAACAATTG---GG
ompA_Rslovaca13-B_U43808.1        AGGGGTAGCTAATGGTACTCCTGCTGGCAGTCCTCAAAACAATTG---GG
ompA_RslovacaCWPP-D_CP003375.1    AGGGGTAGCTAATGGTACTCCTGCTGGCAGTCCTCAAAACAATTG---GG
ompA_Rhhaem_endosymb_KU895509.    AGGGGTAGCTAATGGTACTCCTGCTGGCAGTCCTCAAAACAATTG---GG
ompA_RhhaemMeppadi_MF405464.2     AGGGGTAGTTAATGGTACTCCTGTTGACGGTCCTCAAGATGATGGTATGG
                                  ******** ************** ** * ******** *  ** *   **

ompA_SL94_MZ970588                CATTTACTTACGGTGGTGATTATAATATCACTGCAGATGCAGCCGATCGT
ompA_RconJC480_MN548866.1         CATTTACTTACGGTGGTGATTATAATATCACTGCAGATGCAGCCGATCGT
ompA_Rslovaca13-B_U43808.1        CATTTACTTACGGTGGTGATTATACTATCACTGCAGATGCAGCCGATCGT
ompA_RslovacaCWPP-D_CP003375.1    CATTTACTTACGGTGGTGATTATACTATCACTGCAGATGCAGCCGATCGT
ompA_Rhhaem_endosymb_KU895509.    CATTTACTTACGGTGGTGATTATAATATCACTGCAGATGCAGCCGATCGT
ompA_RhhaemMeppadi_MF405464.2     CATTTACTTACGGTGGTGATCATACTATCACTGCAGATGAAGCCGGTCGT
                                  ******************** *** ************** ***** ****

ompA_SL94_MZ970588                ATTATTACGGCTATAAATGTTGCGGGTACTACTCCTGTAGGTCTAAATAT
ompA_RconJC480_MN548866.1         ATTATTACGGCTATAAATGTTGCGGGTACTACTCCTGTAGGTCTAAATAT
ompA_Rslovaca13-B_U43808.1        ATTATTACGGCTATAAATGTTGCGGGTACTACTCCCGTAGGTCTAAATAT
ompA_RslovacaCWPP-D_CP003375.1    ATTATTACGGCTATAAATGTTGCGGGTACTACTCCCGTAGGTCTAAATAT
ompA_Rhhaem_endosymb_KU895509.    ATTATTACGGCTATAAATGTTGCGGGTACTACTCCTGTAGGTCTAAATAT
ompA_RhhaemMeppadi_MF405464.2     ATTATTACGGCTATAAATGTTGCGGGTACTACTCCAGTAGGTCTAAATAT
                                  *********************************** **************

ompA_SL94_MZ970588                TGCTCAAAATACCGTCGTTGGTTCGATTATAACGAGAGGTAACTTGTTGC
ompA_RconJC480_MN548866.1         TGCTCAAAATACCGTCGTTGGTTCGATTATAACGAGAGGTAACTTGTTGC
ompA_Rslovaca13-B_U43808.1        TGCTCAAAATACCGTCGTTGGTTCGATTATAACGAGAGGTAACTTGTTGC
ompA_RslovacaCWPP-D_CP003375.1    TGCTCAAAATACCGTCGTTGGTTCGATTATAACGAGAGGTAACTTGTTGC
ompA_Rhhaem_endosymb_KU895509.    TGCTCAAAATACCGTCGTTGGTTCGATTATAACGAGAGGTAACTTGTTGC
ompA_RhhaemMeppadi_MF405464.2     TACTCAAAATACCGTCGTTGGTTCGATTGTGACGGGAGGTAACTTGTTGC
                                  * ************************** * *** ***************
```

```
ompA_SL94_MZ970588                 CTGTTACTATTACTGCCGGCAAAAGCTTAACTTTAAACGGTAATAATGCT
ompA_RconJC480_MN548866.1          CTGTTACTATTACTGCCGGCAAAAGCTTAACTTTAAACGGTAATAATGCT
ompA_Rslovaca13-B_U43808.1         CTGTTACTATTAATGCCGGCAAAAGCTTAACTTTAAACGGTAATAATGCT
ompA_RslovacaCWPP-D_CP003375.1     CTGTTACTATTAATGCCGGCAAAAGCTTAACTTTAAACGGTAATAATGCT
ompA_Rhhaem_endosymb_KU895509.     CTGTTACTATTACTGCCGGCAAAAGCTTAACTTTAAACGGTAATAATGCT
ompA_RhhaemMeppadi_MF405464.2      CTGTTACTATTACTGCCGGTAAAAGCTTAACTTTAAACGGTACTAATGCT
                                   ************ ****** ********************** *******

ompA_SL94_MZ970588                 GTTGCTGCAAATAATGGTTTTGATGCTCCTGCCGATAATTATACAGGTTT
ompA_RconJC480_MN548866.1          GTTGCTGCAAATAATGGTTTTGATGCTCCTGCCGATAATTATACAGGTTT
ompA_Rslovaca13-B_U43808.1         GTTGCTGCAAATCATGGTTTTGATTCTCCTGCCGATAATTATACAGGTTT
ompA_RslovacaCWPP-D_CP003375.1     GTTGCTGCAAATCATGGTTTTGATTCTCCTGCCGATAATTATACAGGTTT
ompA_Rhhaem_endosymb_KU895509.     GTTGCTGCAAATAATGGTTTTGATGCTCCTGCCGATAATTATACAGGTTT
ompA_RhhaemMeppadi_MF405464.2      GTTGCTGCAAATCATGGTTTTGATGCTCCTGCCGATAATTATACAGGTTT
                                   ************ ********** **************************

ompA_SL94_MZ970588                 AGGAAATATAACTTT-----------------------------------
ompA_RconJC480_MN548866.1          AGGAAATATAACTTTAGGGGGAGCGAATGCTGCACTAATTATACAATCTG
ompA_Rslovaca13-B_U43808.1         AGGAAATATAGCTTTAGGGGGAGCGAATGCTGCACTAATTATACAATCTG
ompA_RslovacaCWPP-D_CP003375.1     AGGAAATATAGCTTTAGGGGGAGCGAATGCTGCACTAATTATACAATCTG
ompA_Rhhaem_endosymb_KU895509.     AGGAAATATAACTTTAGGGGGAGCGAATGCTGCACTAATTATACAATCTG
ompA_RhhaemMeppadi_MF405464.2      AGGAAATATAACTTTAGGGGGAGCGAATGCTGCACTAATTATACAATCTG
                                   ********** ***************************************

ompA_SL94_MZ970588                 --------------------------------------------------
ompA_RconJC480_MN548866.1          CAGCCCCGGCAAAGATAACACTTGCAGGAAATATAAATGGAGGAGGTATA
ompA_Rslovaca13-B_U43808.1         CAGCCCCGGCAAAGATAACACTTGCAGGAAATATAAATGGAGGAGGTATA
ompA_RslovacaCWPP-D_CP003375.1     CAGCCCCGGCAAAGATAACACTTGCAGGAAATATAAATGGAGGAGGTATA
ompA_Rhhaem_endosymb_KU895509.     CAGCCCCGGCAAAGATAACACTTGCAGGAAATATAAATGGAGGAGGTATA
ompA_RhhaemMeppadi_MF405464.2      TAACCCCGGCAAAGATAACACTTGCAGGAAATATAGATGGAGGAGGTATA
                                    * ******************************** **************

ompA_SL94_MZ970588                 --------------------------------------------------
ompA_RconJC480_MN548866.1          ATAACTGTCAAGAC------------------------------------
ompA_Rslovaca13-B_U43808.1         ATAACTGTCAAGAC------------------------------------
ompA_RslovacaCWPP-D_CP003375.1     ATAACTGTCAAGACAGATGCTGCCATTAACGGAACAATAGGTAATACAAA
ompA_Rhhaem_endosymb_KU895509.     --------------------------------------------------
ompA_RhhaemMeppadi_MF405464.2      ATAACTGTCAATACAGATGCTGCCATTAACGGAAC---------------
                                   *********** ***********************
```

***omp*B alignment**

```
ompB_SL94_MZ970572               --------------------------------------------------
ompB_RconJC480_MN581995.1        ---TACAGCTACCATAGTAGCCAGTTTTGCAGGTTCAGCTATGGGTGCTG
ompB_Rslovaca13-B_AF123723.2     TTCTACAGCTACCATAGTAGCCAGTTTTGCAGGTTCAGCTATGGGTGCTG
                                    ***********************************************

ompB_SL94_MZ970572               --------------------------------------------------
ompB_RconJC480_MN581995.1        CTGTACAGCAGAATAGAACAACAAACGGAGTTGCTACAACTGTTGATGGT
ompB_Rslovaca13-B_AF123723.2     CTATACAGCAGAATAGAACAACAAACGGAGTTGCTACAACTGTTGATGGT
                                 ** ***********************************************

ompB_SL94_MZ970572               -------------------------------------------CTCTAAA
ompB_RconJC480_MN581995.1        GCGGGATTTGACCAAGATGCAGTTCCTGCAAATGTTGCAGTTGCTCTAAA
ompB_Rslovaca13-B_AF123723.2     GCGGGATTTGACCAAACTGCAGTTCCTGCAAATGTTGCAGTTGCTCTAAA
                                 ***************  *********************************

ompB_SL94_MZ970572               TGCAGTTATTACTGCTAGTGCTAATAAGGCTATTAATTTAAATACTCCAG
ompB_RconJC480_MN581995.1        TGCAGTTATTACTGCTAGTGCTAATCAGGCTATTAATTTAAATACTCCAG
ompB_Rslovaca13-B_AF123723.2     TGCAGTTATTACTGCTAATGCTCATAAGGGTATTAATTTAAATACTCCAG
                                 ***************** **** ** *** ********************

ompB_SL94_MZ970572               CCGGTAGTTTTAACGGTTTGTTTTTAGATACTGCAAACAATTTAGCAGTG
```

```
ompB_RconJC480_MN581995.1        CCGGTAGTTTTAACGGTTTGTTTTTAGATACTGCAAACAATTTAGCAGTG
ompB_Rslovaca13-B_AF123723.2     CCGGTAGTTTTAACGGTTTGTTTTTAGATACTGCAAACAATTTAGCAGTG
                                 **************************************************

ompB_SL94_MZ970572               GCAGTGAGTGAAGATACTACCTTAGGGTTCATCACTAATACTGTTAATAA
ompB_RconJC480_MN581995.1        GCAGTGAGTGAAGATACTACCTTAGGGTTCATCACTAATACTGTTAATAA
ompB_Rslovaca13-B_AF123723.2     ACAGTGAGTGAAGATACTACCTTAGGGTTCATCACTAATGTTGTTAATAA
                                  **************************************  *********

ompB_SL94_MZ970572               CGCTAACTTCTTTAACCTTACGCTTCATGCCGGTAAAACTCTTACTATAA
ompB_RconJC480_MN581995.1        CGCTAACTTCTTTAACCTTACGCTTCATGCCGGTAAAACTCTTACTATAA
ompB_Rslovaca13-B_AF123723.2     CGCTAACTTCTTTAACCTTACGCTTAATGCCGGTAAAACTCTTACTATAA
                                 ************************* ************************

ompB_SL94_MZ970572               CAGGTCAAGGTATTACTAATGCACAAGCTGCTGCTACAAAAAATGTTCAA
ompB_RconJC480_MN581995.1        CAGGTCAAGGTATTACTAATGCACAAGCTGCTGCTACAAAAAATGTTCAA
ompB_Rslovaca13-B_AF123723.2     CAGGTCAAGGTATTACTAATGCACAAGCTGCTGCTACAAAAAATGCTCAA
                                 ********************************************* ****

ompB_SL94_MZ970572               AATCTTGTTGTACAATTTAATAATGGTGCTGCTATTGACAATAATGATCT
ompB_RconJC480_MN581995.1        AATCTTGTTGTACAATTTAATAATGGTGCTGCTATTGACAATAATGATCT
ompB_Rslovaca13-B_AF123723.2     AATGTTGTTGTACAATGTAATAATGGTGCTGCTATTGGCAATAATGATCT
                                 *** ************ ******************** ************

ompB_SL94_MZ970572               TAAAGGTGTAGGAAGAATAGACTGCGGTGCTGCGGCTTCTACATTAGTAT
ompB_RconJC480_MN581995.1        TAAAGGTGTAGGAAGAATAGACTGCGGTGCTGCGGCTTCTACATTAGTAT
ompB_Rslovaca13-B_AF123723.2     TAAAGGTGTAGGAAGCATAGACTTCGGTGCTGCGGCTTCTACATTAGTAT
                                 *************** ******* **************************

ompB_SL94_MZ970572               TTAATTTAGCAAATCCTACAACTCAAAAAGCTCCTCTTATACTTGGAGAT
ompB_RconJC480_MN581995.1        TTAATTTAGCAAATCCTACAACTCAAAAAGCTCCTCTTATACTTGGAGAT
ompB_Rslovaca13-B_AF123723.2     TTAATTTAGCAAATCCTACAACTCAAAAAGCTCCTCTTATACTTGGAGAT
                                 **************************************************

ompB_SL94_MZ970572               AATGCTGTAATAGTGAATGGTGCTAACGGTACATTAAATGTTACTAATGG
ompB_RconJC480_MN581995.1        AATGCTGTAATAGTTAATGGTGCTAACGGTACATTAAATGTTACTAATGG
ompB_Rslovaca13-B_AF123723.2     AATGCTTTAATAGTTAATGGTGCTAACGGTACATTAAATGTTACTAATGG
                                 ****** ******* ***********************************

ompB_SL94_MZ970572               ATTTATTCAAGTTTCAAATAAAAGTTTTGCTACTGTTAATGCAATTAATA
ompB_RconJC480_MN581995.1        ATTTATTCAAGTTTCAAATAAAAGTTTTGCTACTGTTAATGCAATTAATA
ompB_Rslovaca13-B_AF123723.2     ATTTATTCAAGTTTCAAGTAAAAGTTTTGCTACTGTTAATGCAATTAATA
                                 ***************** ********************************

ompB_SL94_MZ970572               TCGGTGACGGTCAAGGTATCATGTTCAATACTGATGCTGATAATGCTAAT
ompB_RconJC480_MN581995.1        TCGGTGACGGTCAAGGTATCATGTTCAATACTGATGCTGATAATGCTAAT
ompB_Rslovaca13-B_AF123723.2     TCGGTGACGGTCAAGGTATCATGTTCAATACTGATGCTAATAATGCTAAT
                                 ************************************** ***********

ompB_SL94_MZ970572               ACTTTAAATTTACAAGCAAATGGTACTACTATTAATTTTAATGGAACAGA
ompB_RconJC480_MN581995.1        ACTTTAAATTTACAAGCAAATGGTACTACTATTAATTTTAATGGAACAGA
ompB_Rslovaca13-B_AF123723.2     GCTTTAAATTTACAAGCAGGTGGTACTACTATTAATTTTAATGGAACAGA
                                  *****************  ******************************

ompB_SL94_MZ970572               CGG-----------------------------------------------
ompB_RconJC480_MN581995.1        CGGTACGGGTAGATTAGTATTATTAAGTAAGAATGCTGCTGCTACCGACT
ompB_Rslovaca13-B_AF123723.2     CGGTACGGGTAGATTAGTATTATTAAGTAAGAATGCTGCTGCTACCGACT
                                 **************************************************

ompB_SL94_MZ970572               --------------------------------------------------
ompB_RconJC480_MN581995.1        TTAACGTTACAGGAAGTTTAGGCGGTAATCTAAAAGGTATTATCGAATTT
ompB_Rslovaca13-B_AF123723.2     TTAACGTTACAGGAAGTTTAGGCGGTAATCTAAAAGGTATTATCGAATTT
                                 **************************************************
```

```
ompB_SL94_MZ970572              --------------------------------------------------
ompB_RconJC480_MN581995.1       AACACTGTTGCAGTAAACGGTCAACTTAAAGCTAATGCAGGCCCTGCTAA
ompB_Rslovaca13-B_AF123723.2    AACACTGTTGCAGTAGACGGTCAACTTAAAGCTAATGCAGGCCCTGCTAA
                                *************** **********************************

ompB_SL94_MZ970572              --------------------------------------------------
ompB_RconJC480_MN581995.1       TGCAGTAATAGGTACTAATAATGGCGCAGGTAGAGCTGCAGGATTTGTTG
ompB_Rslovaca13-B_AF123723.2    TGCAGTAATAGGTACTAATAATGGCGCAGGTAGAGCTGCAGGATTTGTTG
                                **************************************************

ompB_SL94_MZ970572              --------------------------------------------------
ompB_RconJC480_MN581995.1       TTAGCGTAGATAATGGTAAGGTAGCAACAATCGATGGACAAGTTTATGCT
ompB_Rslovaca13-B_AF123723.2    TTAGCGTAGATAATGGTAAGGTAGCAACAATCGATGGACAAGTTTATGCT
                                **************************************************

ompB_SL94_MZ970572              --------------------------------------------------
ompB_RconJC480_MN581995.1       AAAGACATGGTTATACAAAGTGCTAATGCAGCTGGACAAGTAAATTTTGG
ompB_Rslovaca13-B_AF123723.2    AAAGACATGGTTATACAAAGTGCTAATGCAGCTGGACAAGTAAATTTTAG
                                ************************************************ *

ompB_SL94_MZ970572              --------------------------------------------------
ompB_RconJC480_MN581995.1       ACACATAGTTGACGTTGGTACAGACGGTACTACTGCCTTTAAAACAGCAG
ompB_Rslovaca13-B_AF123723.2    ACACATAGTTGACGTTGGTACAGACGGTACTACTGCCTTTAAAACAGCAG
                                **************************************************

ompB_SL94_MZ970572              --------------------------------------------------
ompB_RconJC480_MN581995.1       CTTCTAAAGTTGCAATAACCCAAAACTCAAACTTTGGTACTACTGATTTC
ompB_Rslovaca13-B_AF123723.2    CTTCTAAAGTTGCAATAACCCAAAACTCAAACTTTGGTAATACTGATTTC
                                *************************************** **********

ompB_SL94_MZ970572              --------------------------------------------------
ompB_RconJC480_MN581995.1       GGTAATCTTGCAGCACAGATTACAGTTCCTAATAATATGACCCTTACCGG
ompB_Rslovaca13-B_AF123723.2    GGTAATCTTGCAGCACAGATTACAGTTCCTAATACTATGACCCTTACCGG
                                ********************************** ***************

ompB_SL94_MZ970572              --------------------------------------------------
ompB_RconJC480_MN581995.1       TAATTTTACAGGTGATGCTAGCAATCCCGGTAATACTGCAGGTGTGATAA
ompB_Rslovaca13-B_AF123723.2    TAATTTTACAGGTGATGCTAGCAATCCCGGTAACACTGCAGGTGTGATAA
                                ********************************* ****************

ompB_SL94_MZ970572              --------------------------------------------------
ompB_RconJC480_MN581995.1       CTTTTAATGCTAACGGTACTTTAGCAAGTGCTAGTGCAGATGCAAATGTT
ompB_Rslovaca13-B_AF123723.2    CTTTTGATGCTAACGGTACTTTAGCAAGTGCTAGTGCAGATGCAAATGTT
                                ***** ********************************************

ompB_SL94_MZ970572              --------------------------------------------------
ompB_RconJC480_MN581995.1       GCGGTGACAAATAATATTACAGCAATTGAAGCATCAGGTGCCGGAGTTGT
ompB_Rslovaca13-B_AF123723.2    GCGGTGACAAATAATATTACAGCAATTGAAGCATCAGGTGCCGGAGTTGT
                                **************************************************

ompB_SL94_MZ970572              --------------------------------------------------
ompB_RconJC480_MN581995.1       CCAATTATCAGGAACACATGCTGCCGAGTTACGTTTAGGAAATGCCGGTT
ompB_Rslovaca13-B_AF123723.2    CCAATTATCAGGAACACATGCTGCCGAGTTACGTTTAGGAAATGCCGGTT
                                **************************************************

ompB_SL94_MZ970572              --------------------------------------------------
ompB_RconJC480_MN581995.1       CTGTCTTTAAACTTGCTGACGGTACAGTTATAAACGGTAAAGTTAACCAA
ompB_Rslovaca13-B_AF123723.2    CTATCTTTAAACTTGCTGACGGTACAGTTATAAACGGTAAAGTTAACCAA
                                ** ***********************************************

ompB_SL94_MZ970572              --------------------------------------------------
ompB_RconJC480_MN581995.1       ACTGCTCTTGTCGCCGGTGTTCTTGCAGCCGGTGCTATTACCTTAGACGG
```

```
ompB_Rslovaca13-B_AF123723.2      ACTGCTCTTGTCGCCGGTGTTCTTGCAGCCGGTGCTATTACCTTAGACGG
                                  **************************************************

ompB_SL94_MZ970572                --------------------------------------------------
ompB_RconJC480_MN581995.1         TAGTGCTACAATTACCGGCGATATAGGGAACGCTGGTGGTGTTGCTGCGT
ompB_Rslovaca13-B_AF123723.2      TAGTGCTACAATTACCGGCGATATAGGGAACGCTGGTGGTGCTGCTGCGT
                                  ***************************************** ********

ompB_SL94_MZ970572                --------------------------------------------------
ompB_RconJC480_MN581995.1         TACAAGGCATTACTTTAGCTAACGATGCTACAAAAACATTAACACTTGGT
ompB_Rslovaca13-B_AF123723.2      TACAAGGCATTACTTTAGCTAACGATGCTACAAAAACATTAACACTTGGT
                                  **************************************************

ompB_SL94_MZ970572                --------------------------------------------------
ompB_RconJC480_MN581995.1         GGAGCAAATATTATCGGTGCTAATGGTGGAACGATTGATTTCCAAGCTAA
ompB_Rslovaca13-B_AF123723.2      GGAGCAAATATTATCGGTGCTAATGGTGGAACGATTGATTTCCAAGCTAA
                                  **************************************************

ompB_SL94_MZ970572                --------------------------------------------------
ompB_RconJC480_MN581995.1         CGGTGGTACTATTAAATTAACAAGCACTCAAAATAATATTGTAGTTGATT
ompB_Rslovaca13-B_AF123723.2      CGGTGGTACTATTAAATTAACAAGCACTCAAAATAATATTGTAGTTGATT
                                  **************************************************

ompB_SL94_MZ970572                --------------------------------------------------
ompB_RconJC480_MN581995.1         TTGATTTAGCTATAACTACTGATCAAACAGGCGTTGTTGATGCGAGTAGC
ompB_Rslovaca13-B_AF123723.2      TTGATTTAGCTATAACTACTGATCAAACAGGCGTTGTTGATGCGAGTAGC
                                  **************************************************

ompB_SL94_MZ970572                --------------------------------------------------
ompB_RconJC480_MN581995.1         CTAACAAATGCTCAAACTTTAAATATTAACGGTAAAATCGGTACTATTGG
ompB_Rslovaca13-B_AF123723.2      CTAACAAATGCTCAAACTTTAACTATTAACGGTAAAATCGGTACTATTGG
                                  ********************** ***************************

ompB_SL94_MZ970572                --------------------------------------------------
ompB_RconJC480_MN581995.1         AGCTAATAATAAAACTCTTGGACAATTTAATATTGGCTCAAGTAAAACAG
ompB_Rslovaca13-B_AF123723.2      AGCTAATAATAAAACTCTTGGACAATTTAATATTGGCTCAAGTAAAACAG
                                  **************************************************

ompB_SL94_MZ970572                --------------------------------------------------
ompB_RconJC480_MN581995.1         CTTTAAGTGACGGTAATGTTGCTATTAACGAGTTAGTTATTGGAAATAAC
ompB_Rslovaca13-B_AF123723.2      CTTTAAGTAACGGTAATGTTGCTATTAACGAGTTAGTTATTGGAAATAAC
                                  ******** *****************************************

ompB_SL94_MZ970572                --------------------------------------------------
ompB_RconJC480_MN581995.1         GGTGCAGTACAATTCGCTCATAATACTTATTTAATAACAAGAACTACAAA
ompB_Rslovaca13-B_AF123723.2      GGTGCAGTACAATTCGCTCATAATACTTATTTAATAACAAGAACTACAAA
                                  **************************************************

ompB_SL94_MZ970572                --------------------------------------------------
ompB_RconJC480_MN581995.1         TGCTGCCGGTCAAGGTAAAATAATATTTAATCCTGTTGTAAATGGTAATA
ompB_Rslovaca13-B_AF123723.2      TGCTGCCGGTCAAGGTAAAATAATATTTAATCCTGTTGTAAATAATAATA
                                  *******************************************  *****

ompB_SL94_MZ970572                --------------------------------------------------
ompB_RconJC480_MN581995.1         CAACTCTTGCAGCCGGTACAAATTTAGGTAGTGCTACAAATCCACTTGCA
ompB_Rslovaca13-B_AF123723.2      CAACTCTTGCAGCCGGTACAAATTTAGGTAGTGCTACAAATCCACTTGCA
                                  **************************************************

ompB_SL94_MZ970572                --------------------------------------------------
ompB_RconJC480_MN581995.1         GAAATTAATTTTGGATCTAAAGGGGTTAATGTTGATACTGTATTAAATGT
ompB_Rslovaca13-B_AF123723.2      GAAATTAATTTTGGATCTAAAGGGGTTAATGTTGATACTGTATTAAATGT
                                  **************************************************
```

```
ompB_SL94_MZ970572              --------------------------------------------------
ompB_RconJC480_MN581995.1       TGGTGAAGGTGTAAATTTATATGCTACTAATATTACTACTACCGATGCTA
ompB_Rslovaca13-B_AF123723.2    TGGTGAAGGTGTAAATTTATATGCTACTAATATTACTACTACCGATGCTA
                                **************************************************

ompB_SL94_MZ970572              --------------------------------------------------
ompB_RconJC480_MN581995.1       ACGTAGGTTCTTTCATCTTTAATGCCGGTGGAACAAATATAGTAAGCGGT
ompB_Rslovaca13-B_AF123723.2    ACGTAGGTTCTTTCATCTTTAATGCCGGTGGAACAAATATAGTAAGCGGT
                                **************************************************

ompB_SL94_MZ970572              --------------------------------------------------
ompB_RconJC480_MN581995.1       ACAGTCGGTGGACAGCAAGGTAATAAGTTTAATACTGTAGCATTAGGTAA
ompB_Rslovaca13-B_AF123723.2    ACAGTCGGTGGACAGCAAGGTAATAAGTTTAATACTGTAGCATTAGATAA
                                ********************************************** ***

ompB_SL94_MZ970572              --------------------------------------------------
ompB_RconJC480_MN581995.1       CGGTACTACTGTTAAGTTCTTAGGTAATGCAACATTTAACGGTAACACTA
ompB_Rslovaca13-B_AF123723.2    CGGTACTACTGTTAAGTTCTTAGGTAATGCAACATTTAACGGTAACACTA
                                **************************************************

ompB_SL94_MZ970572              --------------------------------------------------
ompB_RconJC480_MN581995.1       CAATTGCAGCTAATTCTACCTTACAAATCGGTGGTAACTATACTGCAGAC
ompB_Rslovaca13-B_AF123723.2    CAATTGCAGCTAATTCTACCTTACAAATCGGTGGTAACTATACTGCAGAC
                                **************************************************

ompB_SL94_MZ970572              --------------------------------------------------
ompB_RconJC480_MN581995.1       TTTGTTGCATCTGCCGATGGTACCGGTATAGTAGAATTCGTTAACACCGG
ompB_Rslovaca13-B_AF123723.2    TTTGTTGCATCTGCCGATGGTACCGGTATAGTAGAATTCGTTAACACCGG
                                **************************************************

ompB_SL94_MZ970572              --------------------------------------------------
ompB_RconJC480_MN581995.1       TCCTATTACCGTAACATTAAACAAACAAGCTGCACCTGTTGTTAATGCTT
ompB_Rslovaca13-B_AF123723.2    TCCTATTACCGTAACATTAAACAAACAAGCTGCACCTGTT---AATGCTT
                                ****************************************   *******

ompB_SL94_MZ970572              --------------------------------------------------
ompB_RconJC480_MN581995.1       TAAAACAAATAACGGTTTCTGGTCCCGGTGACGTAGTGATTAATGAGATA
ompB_Rslovaca13-B_AF123723.2    TAAAACAAATAACGGTTTCTGGTCCCGGTAACGTAGTGATTAATGAGATA
                                ***************************** ********************

ompB_SL94_MZ970572              --------------------------------------------------
ompB_RconJC480_MN581995.1       GGTAATGCAGGTAATTATCATGGTGCCGTGACTGATACGATTGCTTTTGA
ompB_Rslovaca13-B_AF123723.2    GGTAATGCAGGTAATTATCATGGTGCCGTGACTGATACGATTGCTTTTGA
                                **************************************************

ompB_SL94_MZ970572              --------------------------------------------------
ompB_RconJC480_MN581995.1       AAATTCAAGTTTAGGTGCAGTTGTATTCTTACCTAGAGGCATTCCATTCA
ompB_Rslovaca13-B_AF123723.2    AAATTCAAGTGTAGGTGCAGTTGTATTCTTACCTAGAGGCATTCCATTCA
                                ********** ***************************************

ompB_SL94_MZ970572              --------------------------------------------------
ompB_RconJC480_MN581995.1       ATGATGCAGGCAACATAATTCCTTTAACAATTAAAAGTACCGTAGGTAAT
ompB_Rslovaca13-B_AF123723.2    ATGATGCAGGCAACATAATTCCTTTAACAATTAAAAGTACCGTAGGTAAT
                                **************************************************

ompB_SL94_MZ970572              --------------------------------------------------
ompB_RconJC480_MN581995.1       AAAACAGCTACAGGTTTTAATGTTCCTAGCGTGGTTGTTTTAGGTGTTGA
ompB_Rslovaca13-B_AF123723.2    AAAACAGCTACAGGTTTTGATGTTCCTAGCGTGATTGTTTTAGGTGTTGA
                                ****************** ************** ****************

ompB_SL94_MZ970572              --------------------------------------------------
ompB_RconJC480_MN581995.1       TAGTGTCATCGCTGACGGTCAAGTAATCGGTGATCAAAATAATATCGTAG
ompB_Rslovaca13-B_AF123723.2    TAGTGTCATCGCTGACGGTCAAGTAATCGGTGATCAAAATAATATCGTAG
```

```
                                    **************************************************

ompB_SL94_MZ970572                  --------------------------------------------------
ompB_RconJC480_MN581995.1           GTCTAGGTCTTGGAAGCGATAACGACATAATCGTTAATGCTACTACATTA
ompB_Rslovaca13-B_AF123723.2        GTCTAGGTCTTGGAAGCGATAACGACATAATCGTTAATGCTACTACATTA
                                    **************************************************

ompB_SL94_MZ970572                  --------------------------------------------------
ompB_RconJC480_MN581995.1           TATGCAGGTATCGGTACTATAAACAATAATCAAGGTACTGTCACACTTAG
ompB_Rslovaca13-B_AF123723.2        TATGCAGGTATCGGTACTATAAACAATAATCAAGGTACTGTCACACTTAG
                                    **************************************************

ompB_SL94_MZ970572                  --------------------------------------------------
ompB_RconJC480_MN581995.1           CGGTGGTATTCCTAATACCCCTGGTACAGTTTATGGCTTAGGCACAGGTA
ompB_Rslovaca13-B_AF123723.2        CGGTGGTATTCCTAATACCCCTGGTACAGTTTATGGCTTAGGCACAGGTA
                                    **************************************************

ompB_SL94_MZ970572                  --------------------------------------------------
ompB_RconJC480_MN581995.1           TTGGCGCTTCAAAGTTCAAGCAAGTAACGTTTACTACAGACTATAACAAC
ompB_Rslovaca13-B_AF123723.2        TTAGCCCTTCAAAGTTCAAGCAAGTAACGTTTACTACAGACTATAACAAC
                                    ** ** ********************************************

ompB_SL94_MZ970572                  --------------------------------------------------
ompB_RconJC480_MN581995.1           TTAGGTAATATTATTGCAACTAACGCAACAATTAATGACGGTGTAACTGT
ompB_Rslovaca13-B_AF123723.2        TTAGGTAATATTATTGCAACTAACGCAACAATTAATGACGGTGTAACTGT
                                    **************************************************

ompB_SL94_MZ970572                  --------------------------------------------------
ompB_RconJC480_MN581995.1           TACTACAGGCGGTATAGCCGGAACAGGTTTTGACGGTAAAATTACTCTTG
ompB_Rslovaca13-B_AF123723.2        TACTACAGGCGGTATAGCCGGAACAAGTTTTAACGGTAAAATTACTCTTG
                                    ************************* ***** ******************

ompB_SL94_MZ970572                  --------------------------------------------------
ompB_RconJC480_MN581995.1           GAAGTGTTAACGGTAACGGTAATGTAAGATTTGCTGACGGTATATTGTCT
ompB_Rslovaca13-B_AF123723.2        GAAGTGTTAACGGTAACGGTAATGTAAGATTTGCTGACGGTATATTGTCT
                                    **************************************************

ompB_SL94_MZ970572                  --------------------------------------------------
ompB_RconJC480_MN581995.1           AATTCTACAAGTATGATTGGTACTACTAAGGCTAATAATGGTACTGTAAC
ompB_Rslovaca13-B_AF123723.2        AATTCTACAAGTATGATTGGTACTACTAAAGCTAATAATGGTACTGTAAC
                                    ***************************** ********************

ompB_SL94_MZ970572                  --------------------------------------------------
ompB_RconJC480_MN581995.1           TTATTTAGGTAATGCATTCGTCGGTAATATAGGTGATTCAGATACTCCTG
ompB_Rslovaca13-B_AF123723.2        TTATTTAGGTAATGCATTAGTCGGTAATATAGGTGATTCAGATACTCCTG
                                    ****************** *******************************

ompB_SL94_MZ970572                  --------------------------------------------------
ompB_RconJC480_MN581995.1           TAGCTTCTGTTAGATTTACAGGTAGTGATGATGGTGCAGGATTACAAGGA
ompB_Rslovaca13-B_AF123723.2        TAGCTTCTGTTAGATTTACAGGTAGTGATGGTAATGCAAGATTACAAGGA
                                    ******************************  * ****  **********

ompB_SL94_MZ970572                  --------------------------------------------------
ompB_RconJC480_MN581995.1           AATATTTATTCACAAGTCATAGACTTTGGTACTTATAACTTAGGTATTTT
ompB_Rslovaca13-B_AF123723.2        AATATTTATTCACAAGTCATAGACTTTGGTACTTATAACTTAGATATTTT
                                    ******************************************* ******

ompB_SL94_MZ970572                  --------------------------------------------------
ompB_RconJC480_MN581995.1           AAATTCTAATGTAATTTTAGGCGGCGGTACTACTGCTATTAACGGTAAAA
ompB_Rslovaca13-B_AF123723.2        AAATTCTAATGTAATTTTAGGCGGCGGTACTACTGCTATTAACGGTAAAA
                                    **************************************************

ompB_SL94_MZ970572                  --------------------------------------------------
```

```
ompB_RconJC480_MN581995.1         TCAATCTTCTTACAAATACTTTAACATTTGCAAGTGGTACTTCAACATGG
ompB_Rslovaca13-B_AF123723.2      TCAATCTTCTTACAAATACTTTAACATTTGCAAGTGGTACTTCAACATGG
                                  **************************************************

ompB_SL94_MZ970572                --------------------------------------------------
ompB_RconJC480_MN581995.1         GGAAACAATACTTCTATTGAAACTACTTTAACATTAGCAAACGGTAATGT
ompB_Rslovaca13-B_AF123723.2      GGAAACAATACTTCTATTGAAACTACTTTAACATTAGCAAACGGTAATAT
                                  ************************************************ *

ompB_SL94_MZ970572                --------------------------------------------------
ompB_RconJC480_MN581995.1         AGGTCACATCGTTATCTTGGAAGGTGCGCAAGTTAATGCAACAACCACAG
ompB_Rslovaca13-B_AF123723.2      AGGTCACATCGTTATCTTGGAAGGTGCACAAGTTAATGCAACAACCACAG
                                  *************************** **********************

ompB_SL94_MZ970572                --------------------------------------------------
ompB_RconJC480_MN581995.1         GAATTACAACCATTAAAGTACAAGATAATGCCAATGCAAATTTCAGTGGT
ompB_Rslovaca13-B_AF123723.2      GAACTACAACCATTAAAGTACAAGATAATGCCAATGCAAATTTCAGTGGT
                                  *** **********************************************

ompB_SL94_MZ970572                --------------------------------------------------
ompB_RconJC480_MN581995.1         ACACAAACTTATACTTTAATCCAAGGTGGTGCTAGATTTAACGGTACTTT
ompB_Rslovaca13-B_AF123723.2      ACACAAACTTATACTTTAATCCAAGGTGGTGCTAGATTTAACGGTACTTT
                                  **************************************************

ompB_SL94_MZ970572                --------------------------------------------------
ompB_RconJC480_MN581995.1         AGGAGGTCCCAACTTTGCTGTAACCGGAAGTAATCGTTTCGTAAATTACG
ompB_Rslovaca13-B_AF123723.2      AGGAGGTCCCAACTTTGCTGTAACCGGAAATAATCGTTTCGTAAATTACG
                                  ***************************** ********************

ompB_SL94_MZ970572                --------------------------------------------------
ompB_RconJC480_MN581995.1         GTTTAATACGTGCTGCTAACCAAGATTATGTAATAACACGTACTAACAAT
ompB_Rslovaca13-B_AF123723.2      GTTTAATACGTGCTGCTAACCAAGATTATGTAATAACACGTACTAACAAT
                                  **************************************************

ompB_SL94_MZ970572                --------------------------------------------------
ompB_RconJC480_MN581995.1         GCAGAAAACGTAGTTACTAATGATATCGCAAATAGTCCGTTTGGAGGTGC
ompB_Rslovaca13-B_AF123723.2      GCAGAAAACGTAGTTACTAATGATATCGCAAATAGTCCGTTTGGAGGTGC
                                  **************************************************

ompB_SL94_MZ970572                --------------------------------------------------
ompB_RconJC480_MN581995.1         ACCGGGTGTAGGTCAGAACGTTACAACATTTGTAAATGCAACTGATACTG
ompB_Rslovaca13-B_AF123723.2      ACCGGGTGTAGGTCAGAACGTTACAACATTTGTAAATGCAACTAATACTG
                                  ******************************************* ******

ompB_SL94_MZ970572                --------------------------------------------------
ompB_RconJC480_MN581995.1         CAGCATATAATAATCTTCTTTTAGCTAAAAATAGTGCTAATTCTGCTAAC
ompB_Rslovaca13-B_AF123723.2      CAGCATATAATAATCTTCTTTTAGCTAAAAATAGTGCTAATTCTGCTAAC
                                  **************************************************

ompB_SL94_MZ970572                --------------------------------------------------
ompB_RconJC480_MN581995.1         TTTGTTGGAGCTATCGTTACCGATACAAGTGCGGCCGTAACTAATGCACA
ompB_Rslovaca13-B_AF123723.2      TTTGTTGGAGCTATCGTTACCGATACAAGTGCGGCCATAACTAATGCACA
                                  ************************************ *************

ompB_SL94_MZ970572                --------------------------------------------------
ompB_RconJC480_MN581995.1         ATTAGATGTAGCTAAAGATATTCAAGCTCAACTTGGTAACAGATTAGGTG
ompB_Rslovaca13-B_AF123723.2      ATTAGAGGTAGCTAAAGATATCCAAGCTCAACTTGGTAACAGATTAGGTG
                                  ****** ************** ****************************

ompB_SL94_MZ970572                --------------------------------------------------
ompB_RconJC480_MN581995.1         CTCTTAGATATTTAGGTACTCCTGAAACTGCTGAAATGGCTGGACCTGAA
ompB_Rslovaca13-B_AF123723.2      CTCTTAGATATTTAGGTACTCCTGAAACTGCTGAAATGGCTGGACCTGAA
                                  **************************************************
```

```
ompB_SL94_MZ970572              --------------------------------------------------
ompB_RconJC480_MN581995.1       GCTGGAGCAATACCGGCTGCGGTTGCTGCAGGTGACGAGGCTGTTGATAA
ompB_Rslovaca13-B_AF123723.2    GCTGGAGCAATACCGGCTGCGGTTGCTGCAGGTGACGAGGCTGTTGATAA
                                **************************************************

ompB_SL94_MZ970572              --------------------------------------------------
ompB_RconJC480_MN581995.1       TGTAGCTTACGGTATATGGGCAAAACCTTTCTATACTGATGCGCATCAAA
ompB_Rslovaca13-B_AF123723.2    TGTAGCTTACGGTATATGGGCAAAACCTTTCTATACTGATGCACATCAAA
                                ****************************************** *******

ompB_SL94_MZ970572              --------------------------------------------------
ompB_RconJC480_MN581995.1       GTAAGAAAGGTGGTTTAGCTGGTTATAAAGCTAAAACCACCGGTGTCGTA
ompB_Rslovaca13-B_AF123723.2    GTAAGAAAGGTGGTTTAGCTGGTTATAAAGCTAAAACCACCGGTGTCGTA
                                **************************************************

ompB_SL94_MZ970572              --------------------------------------------------
ompB_RconJC480_MN581995.1       ATCGGTTTAGATACGCTAGCTAACGATAACTTAATGATCGGTGCTGCTAT
ompB_Rslovaca13-B_AF123723.2    ATCGGTTTAGATACGCTAGCTAACGATAACTTAATGATCGGTGCTGCTAT
                                **************************************************

ompB_SL94_MZ970572              --------------------------------------------------
ompB_RconJC480_MN581995.1       CGGTATCACTAAAACTGATATAAAACACCAAGATTATAAGCAAGGTGATA
ompB_Rslovaca13-B_AF123723.2    CGGTATCACTAAAACTGATATAAAACACCAAGATTATAAGAAAGGTGATA
                                **************************************** *********

ompB_SL94_MZ970572              --------------------------------------------------
ompB_RconJC480_MN581995.1       AAACCGACGTTAACGGTTTCTCATTCTCTTTATATGGTGCCCAGCAGCTT
ompB_Rslovaca13-B_AF123723.2    AAACCGACGTTAACGGTTTCTCATTCTCTCTATATGGTGCCCAGCAGCTT
                                ***************************** ********************

ompB_SL94_MZ970572              --------------------------------------------------
ompB_RconJC480_MN581995.1       GTTAAGAACTTCTTTGCTCAAGGTAGTGCAATATTTAGCTTAAACCAAGT
ompB_Rslovaca13-B_AF123723.2    GTTAAGAACTTCTTTGCTCAAGGTAGTGCAATATTTAGCTTAAACCAAGT
                                **************************************************

ompB_SL94_MZ970572              --------------------------------------------------
ompB_RconJC480_MN581995.1       GAAGAACAAAAGTCAGCGTTACTTCTTCGATGCTAACGGTAATATGAGCA
ompB_Rslovaca13-B_AF123723.2    GAAGAACAAAAGTCAGCGTTACTTCTTCGATGCTAACGGTAATATGAGCA
                                **************************************************

ompB_SL94_MZ970572              --------------------------------------------------
ompB_RconJC480_MN581995.1       AGCAAATTGCTGCCGGTCATTACGATAACATGACATTCGGTGGTAACTTA
ompB_Rslovaca13-B_AF123723.2    AGCAAATTGCTGCCGGTCATTACGATAACATGACATTCGGTGGTAACTTA
                                **************************************************

ompB_SL94_MZ970572              --------------------------------------------------
ompB_RconJC480_MN581995.1       ACAGTCGGTTATGATTACAATGCAATGCAAGGTGTGTTAGTAACTCCAAT
ompB_Rslovaca13-B_AF123723.2    ACAGTCGGTTATGATTACAATGCAATGCAAGGTGTGTTAGTAACTCCAAT
                                **************************************************

ompB_SL94_MZ970572              --------------------------------------------------
ompB_RconJC480_MN581995.1       GGCAGGACTTAGCTACTTAAAGTCTTCTGACGAAAACTACAAAGAAACCG
ompB_Rslovaca13-B_AF123723.2    GGCAGGACTTAGCTACTTAAAGTCTTCTGACGAAAACTACAAAGAAACCG
                                **************************************************

ompB_SL94_MZ970572              --------------------------------------------------
ompB_RconJC480_MN581995.1       GTACAACAGTTGCAAACAAGCAAGTTAACAGCAAATTTAGCGATAGAACC
ompB_Rslovaca13-B_AF123723.2    GTACAACAGTTGCAAACAAGCAAGTTAACAGCAAATTTAGCGATAGAACC
                                **************************************************

ompB_SL94_MZ970572              --------------------------------------------------
ompB_RconJC480_MN581995.1       GATTTAATAGTAGGTGCTAAAGTAGCCGGTAGTACTATGAACATAACTGA
```

```
ompB_Rslovaca13-B_AF123723.2      GATTTAATAGTAGGTGCTAAAGTAGCCGGCAGTACTATGAACATAACTGA
                                  ***************************** ********************

ompB_SL94_MZ970572                --------------------------------------------------
ompB_RconJC480_MN581995.1         TCTTGCGGTATATCCGGAAGTTCACGCTTTTGTGGTTCACAAAGTAACCG
ompB_Rslovaca13-B_AF123723.2      TCTTGCGGTATATCCGGAAGTTCACGCTTTTGTGGTTCACAAAGTAACCG
                                  **************************************************

ompB_SL94_MZ970572                --------------------------------------------------
ompB_RconJC480_MN581995.1         GTAGATTATCTAAAACTCAGTCTGTATTAGACGGACAAGTTACTCCGTGT
ompB_Rslovaca13-B_AF123723.2      GTAGATTATCTAAAACTCAGTCTGTATTAGACGGACAAGTTACTCCGTGT
                                  **************************************************

ompB_SL94_MZ970572                --------------------------------------------------
ompB_RconJC480_MN581995.1         ATCAGCCAGCCTGATAGAACCGCTAAAACATCTTATAATTTAGGTTTAAG
ompB_Rslovaca13-B_AF123723.2      ATCAGCCAGCCTGACAGAACCGCTAAAACATCTTATAATTTAGGTTTAAG
                                  ************** ***********************************

ompB_SL94_MZ970572                --------------------------------------------------
ompB_RconJC480_MN581995.1         TGCAAGCATTAGATCTGATGCTAAGATGGAGTACGGAATCGGTTACGATG
ompB_Rslovaca13-B_AF123723.2      TGCAAGCATAAGATCTGATGCTAAGATGGAGTACGGAATCGGTTACGATG
                                  ********* ****************************************

ompB_SL94_MZ970572                --------------------------------------------------
ompB_RconJC480_MN581995.1         CTCAGATTTCAAGTAAATATACTGCACATCAAGGTACTCTAAAAGTCCGT
ompB_Rslovaca13-B_AF123723.2      CTCAGATTTCAAGTAAATATACTGCACATCAAGGTACTCT----------
                                  ****************************************

ompB_SL94_MZ970572                ------------
ompB_RconJC480_MN581995.1         GTAAACTTCTAA
ompB_Rslovaca13-B_AF123723.2      ------------
```

## ***glt*A alignment**

```
gltA_SL94_MZ970594                  --------------------------------------------------
gltA_RendosymbRhhaem_KU895508.      --------------------------------------------------
gltA_RcconJC480_MN581991.1          --------------------TGATTCAGAATTTGCTGAATTAAAAATCAG
gltA_RslovacaD-CWPP_CP003375.1      ATGACCAATGAAAATAATAATGATTCAGAATTTGCTGAATTAAAAATCAG
gltA_RendosymbMeppadi_MF405463      --------------------------------------------------
                                                        ******************************

gltA_SL94_MZ970594                  --------------------------------------------------
gltA_RendosymbRhhaem_KU895508.      --------------------------------------------------
gltA_RconJC480_MN581991.1           AGGAAAAATATTTAAATTACCTATACTTAAAGCAAGTATCGGTGAGGATG
gltA_RslovacaD-CWPP_CP003375.1      AGGAAAAATATTTAAATTACCTATACTTAAAGCAAGTATCGGTGAGGATG
gltA_RendosymbMeppadi_MF405463      --------------------------------------------------
                                    **************************************************

gltA_SL94_MZ970594                  --------------------------------------------------
gltA_RendosymbRhhaem_KU895508.      --------------------------------------------------
gltA_RconJC480_MN581991.1           TAATCGATATAAGTAGGGTATCTGCGGAAGCCGATTGCTTTACTTACGAT
gltA_RslovacaD-CWPP_CP003375.1      TAATCGATATAAGTAGGGTATCTGCGGAAGCCGATTGCTTTACTTACGAC
gltA_RendosymbMeppadi_MF405463      --------------------------------------------------
                                    *************************************************

gltA_SL94_MZ970594                  --------------------------------------------------
gltA_RendosymbRhhaem_KU895508.      --------------------------------------------------
gltA_RconJC480_MN581991.1           CCGGGTTTTATGTCTACTGCTTCTTGTCAGTCTACTATCACCTATATAGA
gltA_RslovacaD-CWPP_CP003375.1      CCGGGTTTTATGTCTACTGCTTCTTGTCAGTCTACTATCACCTATATAGA
gltA_RendosymbMeppadi_MF405463      --------------------------------------------------
                                    **************************************************
```

```
gltA_SL94_MZ970594                    --------------------------------------------------
gltA_RendosymbRhhaem_KU895508.        --------------------------------------------------
gltA_RconJC480_MN581991.1             CGGTGATAAAGGAATCTTGCGGCATCGAGGATATGATATTAAAGACTTAG
gltA_RslovacaD-CWPP_CP003375.1        CGGTGATAAAGGAATCTTGCGGCATCGAGGATATGATATTAAAGACTTAG
gltA_RendosymbMeppadi_MF405463        --------------------------------------------------
                                      **************************************************

gltA_SL94_MZ970594                    --------------------------------------------------
gltA_RendosymbRhhaem_KU895508.        --------------------------------------------------
gltA_RconJC480_MN581991.1             CTGAGAAAAGTGATTTTTTAGAAGTGGCATATTTACTGATTTATGGGGAA
gltA_RslovacaD-CWPP_CP003375.1        CTGAGAAAAGTGATTTTTTAGAAGTGGCATATTTACTGATTTATGGGGAA
gltA_RendosymbMeppadi_MF405463        --------------------------------------------------
                                      **************************************************

gltA_SL94_MZ970594                    --------------------------------------------------
gltA_RendosymbRhhaem_KU895508.        --------------------------------------------------
gltA_RconJC480_MN581991.1             CTACCAAGTGGCGAGCAGTATAATAATTTCACTAAACAGGTTGCTCATCA
gltA_RslovacaD-CWPP_CP003375.1        CTACCAAGTGGTGAGCAGTATAATAATTTCACTAAACAGGTTGCTCATCA
gltA_RendosymbMeppadi_MF405463        --------------------------------------------------
                                      *********** **************************************

gltA_SL94_MZ970594                    --------------------------------------------------
gltA_RendosymbRhhaem_KU895508.        --------------------------------------------------
gltA_RconJC480_MN581991.1             TTCATTAGTGAATGAAAGATTACACTATTTATTTCAAACCTTTTGTAGCT
gltA_RslovacaD-CWPP_CP003375.1        TTCATTAGTGAATGAAAGATTACACTATTTATTTCAAACCTTTTGTAGCT
gltA_RendosymbMeppadi_MF405463        --------------------------------------------------
                                      **************************************************

gltA_SL94_MZ970594                    --------------------------------------------------
gltA_RendosymbRhhaem_KU895508.        --------------------------------------------------
gltA_RconJC480_MN581991.1             CTTCTCATCCTATGGCTATTATGCTTGCGGCTGTCGGTTCTCTTTCGGCA
gltA_RslovacaD-CWPP_CP003375.1        CTTCTCATCCTATGGCTATTATGCTTGCGGCTGTCGGTTCTCTTTCGGCA
gltA_RendosymbMeppadi_MF405463        --------------------------------------------------
                                      **************************************************

gltA_SL94_MZ970594                    --------------------------------------------------
gltA_RendosymbRhhaem_KU895508.        --------------------------------------------------
gltA_RconJC480_MN581991.1             TTTTATCCTGATTTATTGAATTTTAAGGAAGCAGATTACGAACTTACCGC
gltA_RslovacaD-CWPP_CP003375.1        TTTTATCCTGATTTATTGAATTTTAAGGAAGCAGATTACGAACTTACCGC
gltA_RendosymbMeppadi_MF405463        --------------------------------------------------
                                      **************************************************

gltA_SL94_MZ970594                    --------------------------------------------------
gltA_RendosymbRhhaem_KU895508.        --------------------------------------------------
gltA_RconJC480_MN581991.1             TATTAGAATGATTGCTAAGATACCTACCATCGCTGCAATGTCTTATAAAT
gltA_RslovacaD-CWPP_CP003375.1        TATTAGAATGATTGCTAAGATACCTACCATCGCTGCAATGTCTTATAAAT
gltA_RendosymbMeppadi_MF405463        --------------------------------------------------
                                      **************************************************

gltA_SL94_MZ970594                    --------------------------------------------------
gltA_RendosymbRhhaem_KU895508.        --------------------------------------------------
gltA_RconJC480_MN581991.1             ATTCTATAGGACAACCGTTTATTTATCCTGATAATTCGTTAGATTTTACC
gltA_RslovacaD-CWPP_CP003375.1        ATTCTATAGGACAACCGTTTATTTATCCTGATAATTCGTTAGATTTTACC
gltA_RendosymbMeppadi_MF405463        --------------------------------------------------
                                      **************************************************

gltA_SL94_MZ970594                    --------------------------------------------------
gltA_RendosymbRhhaem_KU895508.        --------------------------------------------------
gltA_RconJC480_MN581991.1             GAAAATTTTCTGCATATGATGTTTGCAACGCCTTGTACGAAATATACAGT
gltA_RslovacaD-CWPP_CP003375.1        GAAAATTTTCTGCATATGATGTTTGCAACGCCTTGTACGAAATATACAGT
gltA_RendosymbMeppadi_MF405463        --------------------------------------------------
                                      **************************************************
```

```
gltA_SL94_MZ970594               --------------------------------------------------
gltA_RendosymbRhhaem_KU895508.   --------------------------------------------------
gltA_RconJC480_MN581991.1        AAATCCAATAATAAAAAATGCTCTTAATAAGATATTTATCCTACATGCCG
gltA_RslovacaD-CWPP_CP003375.1   AAATCCAATAATAAAAAATGCTCTTAATAAGATATTTATCCTACATGCCG
gltA_RendosymbMeppadi_MF405463   --------------------------------------------------
                                 **************************************************

gltA_SL94_MZ970594               --------------------------------------------------
gltA_RendosymbRhhaem_KU895508.   --------------------------------------------------
gltA_RconJC480_MN581991.1        ATCATGAGCAGAATGCTTCTACTTCAACAGTCCGAATTGCCGGCTCATCC
gltA_RslovacaD-CWPP_CP003375.1   ATCATGAGCAGAATGCTTCTACTTCAACAGTCCGAATTGCCGGCTCATCC
gltA_RendosymbMeppadi_MF405463   --------------------------------------------------
                                 **************************************************

gltA_SL94_MZ970594               --------------------------------------------------
gltA_RendosymbRhhaem_KU895508.   ------------------------------------------------GG
gltA_RconJC480_MN581991.1        GGAGCTAACCCTTTTGCTTGTATTAGCACGGGTATTGCCTCACTTTGGGG
gltA_RslovacaD-CWPP_CP003375.1   GGAGCTAACCCTTTTGCTTGTATTAGCACGGGTATTGCCTCACTTTGGGG
gltA_RendosymbMeppadi_MF405463   --------------------------------------------------
                                 **************************************************

gltA_SL94_MZ970594               ---------------GGCTAATGAAGCGGTAATAAATATGCTTAAAGAAA
gltA_RendosymbRhhaem_KU895508.   ACCTGCTCACGGCGGGGCTAATGAAGCGGTAATAAATATGCTTAAAGAAA
gltA_RconJC480_MN581991.1        ACCTGCTCACGGCGGGGCTAATGAAGCGGTAATAAATATGCTTAAAGAAA
gltA_RslovacaD-CWPP_CP003375.1   ACCTGCTCACGGCGGGGCTAATGAAGCGGTAATAAATATGCTTAAAGAAA
gltA_RendosymbMeppadi_MF405463   -------------------------------------------------A
                                 **************************************************

gltA_SL94_MZ970594               TCGGTAGTTCTGAGTATATTCCTAAATATATAGCGAAAGCTAAGGATAAA
gltA_RendosymbRhhaem_KU895508.   TCGGTAGTTCTGAGTATATTCCTAAATATATAGCGAAAGCTAAGGATAAA
gltA_RconJC480_MN581991.1        TCGGTAGTTCTGAGTATATTCCTAAATATATAGCGAAAGCTAAGGATAAA
gltA_RslovacaD-CWPP_CP003375.1   TCGGTAGTTCTGAGTATATTCCTAAATATATAGCTAAAGCTAAGGATAAA
gltA_RendosymbMeppadi_MF405463   TCGGTAGTTCAGAGTATATTCCTAAATATATAGCTAAAGCTAAGGATAGA
                                 ********** *********************** ************* *

gltA_SL94_MZ970594               AATGATCCATTTAGATTAATGGGTTTTGGTCATCGTGTATATAAAAACTA
gltA_RendosymbRhhaem_KU895508.   AATGATCCATTTAGATTAATGGGTTTTGGTCATCGTGTATATAAAAACTA
gltA_RconJC480_MN581991.1        AATGATCCATTTAGATTAATGGGTTTTGGTCATCGTGTATATAAAAACTA
gltA_RslovacaD-CWPP_CP003375.1   AATGATCCATTTAGATTAATGGGTTTTGGTCATCGTGTATATAAAAACTA
gltA_RendosymbMeppadi_MF405463   AATGATCCATTTAGGTTAATGGGTTTTGGTCATCGTATATATAAAAACTA
                                 ************** ********************* *************

gltA_SL94_MZ970594               TGACCCGCGTGCCGCAGTACTTAAAGAAACGTGCAAAGAAGTATTAAAGG
gltA_RendosymbRhhaem_KU895508.   TGACCCGCGTGCCGCAGTACTTAAAGAAACGTGCAAAGAAGTATTAAAGG
gltA_RconJC480_MN581991.1        TGACCCGCGTGCCGCAGTACTTAAAGAAACGTGCAAAGAAGTATTAAAGG
gltA_RslovacaD-CWPP_CP003375.1   TGACCCGCGTGCCGCAGTACTTAAAGAAACGTGCAAAGAAGTATTAAAGG
gltA_RendosymbMeppadi_MF405463   TGACCCGCGTGCCGCAGTACTTAAAGAAACGTGCAAAGAAGTATTAAAGG
                                 **************************************************

gltA_SL94_MZ970594               AACTCGGGCAGCTAGACAACAATCCGCTCTTACAAATAGCAATAGAACTT
gltA_RendosymbRhhaem_KU895508.   AACTCGGGCAGCTAGACAACAATCCGCTCTTACAAATAGCAATAGAACTT
gltA_RconJC480_MN581991.1        AACTCGGGCAGCTAGACAACAATCCGCTCTTACAAATAGCAATAGAACTT
gltA_RslovacaD-CWPP_CP003375.1   AACTCGGGCAGCTAGACAACAATCCGCTCTTACAAATAGCAATAGAACTT
gltA_RendosymbMeppadi_MF405463   AACTCGGGCAGCTAGACAACAATCCGCTCTTACAAATAGCAATAGAACTT
                                 **************************************************

gltA_SL94_MZ970594               GAAGCTATCGCTCTTAAAGATGAATATTTTATTGAGAGAAAATTATATCC
gltA_RendosymbRhhaem_KU895508.   GAAGCTATCGCTCTTAAAGATGAATATTTTATTGAGAGAAAATTATATCC
gltA_RconJC480_MN581991.1        GAAGCTATCGCTCTTAAAGATGAATATTTTATTGAGAGAAAATTATATCC
gltA_RslovacaD-CWPP_CP003375.1   GAAGCTATTGCTCTTAAAGATGAATATTTTATTGAGAGAAAATTATATCC
gltA_RendosymbMeppadi_MF405463   GAAGCTATCGCTCTTAAAGATGAATATTTTATTGAGAGAAAATTATATCC
                                 ******** *****************************************
```

```
gltA_SL94_MZ970594                  AAATGTTGATTTTTATTCGGGTATTATCTATAAAGCTATGGGTATACCGT
gltA_RendosymbRhhaem_KU895508.      AAATGTTGATTTTTATTCGGGTATTATCTATAAAGCTATGGGTATACCGT
gltA_RconJC480_MN581991.1           AAATGTTGATTTTTATTCGGGTATTATCTATAAAGCTATGGGTATACCGT
gltA_RslovacaD-CWPP_CP003375.1      AAATGTTGATTTTTATTCGGGTATTATCTATAAAGCTATGGGTATACCGT
gltA_RendosymbMeppadi_MF405463      AAATGTTGATTTTTATTCGGGTATTATCTATAAAGCTATGGGTATACCGT
                                    **************************************************

gltA_SL94_MZ970594                  CGCAAA--------------------------------------------
gltA_RendosymbRhhaem_KU895508.      CGCAAATGTTCACTGTACTTTTTGCAAT----------------------
gltA_RconJC480_MN581991.1           CGCAAATGTTCACGGTACTTTTTGCAATAGCAAGAACCGTAGGCTGGATG
gltA_RslovacaD-CWPP_CP003375.1      CGCAAATGTTCACGGTACTTTTTGCAATAGCAAGAACCGTAGGCTGGATG
gltA_RendosymbMeppadi_MF405463      CGCAAATGT-----------------------------------------
                                    ************* ************************************

gltA_SL94_MZ970594                  --------------------------------------------------
gltA_RendosymbRhhaem_KU895508.      --------------------------------------------------
gltA_RconJC480_MN581991.1           GCACAATGGAAAGAAATGCACGAAGACCCTGAACAAAAAATCAGCAGACC
gltA_RslovacaD-CWPP_CP003375.1      GCACAATGGAAAGAAATGCACGAAGACCCTGAACAAAAAATCAGCAGACC
gltA_RendosymbMeppadi_MF405463      --------------------------------------------------
                                    **************************************************

gltA_SL94_MZ970594                  --------------------------------------------------
gltA_RendosymbRhhaem_KU895508.      --------------------------------------------------
gltA_RconJC480_MN581991.1           TAGA----------------------------------------------
gltA_RslovacaD-CWPP_CP003375.1      TAGACAGCTTTACACCGGTTATGTACATAGAGAGTATAAGGGTATTCGGG
gltA_RendosymbMeppadi_MF405463      --------------------------------------------------
                                    ****

gltA_SL94_MZ970594                  --------
gltA_RendosymbRhhaem_KU895508.      --------
gltA_RconJC480_MN581991.1           --------
gltA_RslovacaD-CWPP_CP003375.1      AGAGGTAA
gltA_RendosymbMeppadi_MF405463      --------
```

---

## ***sca*4 alignment**

```
sca4_SL94_MZ970562                  --------------------------------------------------
sca4_Rslovaca_D-CWPP_CP003375.      ATGAGTAAAGACGGTAACCTAGATACAAGTGAATTTGATCCTTTAGCAAA
sca4_RconJC480_MN581999.1           --------------------------------------------------

sca4_SL94_MZ970562                  -AAGGAATATACAGAAGAACAAAAGCAAACATTAGAACAAGAGCAAAAAG
sca4_Rslovaca_D-CWPP_CP003375.      TAAGGAATATACAGAAGAACAAAAGCAAACATTAGAACAAGAGCAAAAAG
sca4_RconJC480_MN581999.1           ---------------------------------AGAACAAGAGCAAAAAG
                                    **************************************************

sca4_SL94_MZ970562                  AATTTTTATCTCAAACTACAACCCCAGCACTAGAAGCTGACGATGGTTTT
sca4_Rslovaca_D-CWPP_CP003375.      AATTTTTATCTCAAACTACAACCCCAGCACTAGAAGCTGACGATGGTTTT
sca4_RconJC480_MN581999.1           AATTTTTATCTCAAACTACAACCCCAGCACTAGAAGCTGACGATGGTTTT
                                    **************************************************

sca4_SL94_MZ970562                  ATCGTTGCTTCTGCATCTTCTGCTCAATCTACCCCTTCAATGAGTGCTTT
sca4_Rslovaca_D-CWPP_CP003375.      ATCGTTACTTCTGCATCTTCTGCTCAATCTACCCCTTCAATGAGTGCTTT
sca4_RconJC480_MN581999.1           ATCGTTGCTTCTGCATCTTCTGCTCAATCTACCCCTTCAATGAGTGCTTT
                                    ****** *******************************************

sca4_SL94_MZ970562                  ATCGGGCAATATCTCTCCTGACAGTCAGACATCAGACCCAATAACCAAGG
sca4_Rslovaca_D-CWPP_CP003375.      ATCGGGCAATATCTCTCCTGACAGTCAGACATCAGACCCAATAACCAAGG
sca4_RconJC480_MN581999.1           ATCGGGCAATATCTCTCCTGACAGTCAGACATCAGACCCAATAACCAAGG
                                    **************************************************
```

```
sca4_SL94_MZ970562              CTGTAAGAGAAACAATTATACAACCGCAAAAAGATAATTTAATAGAACAA
sca4_Rslovaca_D-CWPP_CP003375.  CTGTAAGAGAAACAATTATACAACCGCAAAAAGATAATTTAATAGAACAA
sca4_RconJC480_MN581999.1       CTGTAAGAGAAACAATTATACAACCGCAAAAAGATAATTTAATAGAACAA
                                **************************************************

sca4_SL94_MZ970562              ATATTAAAAGACCTGGCAGCCCTTACAGACCGTGATTTAGCTGAACAAAA
sca4_Rslovaca_D-CWPP_CP003375.  ATATTAAAAGACCTGGCAGCCCTTACAGACCGTGATTTAGCTGAACAAAA
sca4_RconJC480_MN581999.1       ATATTAAAAGACCTGGCAGCCCTTACAGACCGTGATTTAGCTGAACAAAA
                                **************************************************

sca4_SL94_MZ970562              AAGAAAAGAAATAGAAGAAGAAAAAGAAAAAGATAAAACATTAAGTACTT
sca4_Rslovaca_D-CWPP_CP003375.  AAGAAAAGAAATAGAAGAAGAAAAAGAAAAAGATAAAACATTAAGTACTT
sca4_RconJC480_MN581999.1       AAGAAAAGAAATAGAAGAAGAAAAAGAAAAAGATAAAACATTAAGTACTT
                                **************************************************

sca4_SL94_MZ970562              TTTTCGGTAATCCGGCTAATAGAGAATTTATTGATAAGGCTTTAGAAAAT
sca4_Rslovaca_D-CWPP_CP003375.  TTTTCGGTAATCCGGCTAATAGAGAATTTATTGATAAGGCTTTAGAAAAT
sca4_RconJC480_MN581999.1       TTTTCGGTAATCCGGCTAATAGAGAATTTATTGATAAGGCTTTAGAAAAT
                                **************************************************

sca4_SL94_MZ970562              CCTGAGCTTAAAAAGAAATTAGAATCAATAGAAATAGCCGGCTATAAAAA
sca4_Rslovaca_D-CWPP_CP003375.  CCTGAGCTTAAAAAGAAATTAGAATCAATAGAAATAGCCGGCTATAAAAA
sca4_RconJC480_MN581999.1       CCTGAGCTTAAAAAGAAATTAGAATCAATAGAAATAGCCGGCTATAAAAA
                                **************************************************

sca4_SL94_MZ970562              TGTGCATAATACATTTAGCGCCGCTAGTGGATACCCTGGTGGATTTAAAC
sca4_Rslovaca_D-CWPP_CP003375.  TGTGCATAATACATTTAGCGCCGCTAGTGGATACCCTGGTGGATTTAAAC
sca4_RconJC480_MN581999.1       TGTGCATAATACATTTAGCGCCGCTAGTGGATACCCTGGTGGATTTAAAC
                                **************************************************

sca4_SL94_MZ970562              CGGTACAGTGGGAAAATCACGTAAGTGCAAGCGATCTTAGAGCAACAGTA
sca4_Rslovaca_D-CWPP_CP003375.  CGGTACAGTGGGAAAATCACGTAAGTGCAAGCGATCTTAGAGCAACAGTA
sca4_RconJC480_MN581999.1       CGGTACAGTGGGAAAATCACGTAAGTGCAAGCGATCTTAGAGCAACAGTA
                                **************************************************

sca4_SL94_MZ970562              GTTAAAAAATGATGCAGGTGATGAACTCTGTACCTTAAATGAAACAACTGT
sca4_Rslovaca_D-CWPP_CP003375.  GTTAAAAAATGATGCAGGTGATGAACTCTGTACCTTAAATGAAACAACTGT
sca4_RconJC480_MN581999.1       GTTAAAAAATGATGCAGGTGATGAACTCTGTACCTTAAATGAAACAACTGT
                                **************************************************

sca4_SL94_MZ970562              TAAAACTAAGCCTTTTACTTTAGCTAAACAAGACGGTACTCAGGTTCAGA
sca4_Rslovaca_D-CWPP_CP003375.  TAAAACTAAGCCTTTTACTTTAGCTAAACAAGACGGTACTCAGGTTCAGA
sca4_RconJC480_MN581999.1       TAAAACTAAGCCTTTTACTTTAGCTAAACAAGACGGTACTCAGGTTCAGA
                                **************************************************

sca4_SL94_MZ970562              TCAGCTCATATAGGGAAATAGATTTTCCTATAAAACTTGATAAAGCCGAT
sca4_Rslovaca_D-CWPP_CP003375.  TCAGCTCATATAGGGAAATAGATTTTCCTATAAAACTTGATAAAGCCGAT
sca4_RconJC480_MN581999.1       TCAGCTCATATAGGGAAATAGATTTTCCTATAAAACTTGATAAAGCCGAT
                                **************************************************

sca4_SL94_MZ970562              GGGTCAATGCATTTATCGATGGTAGCATTAAAAGCTGATGGCACAAAGCC
sca4_Rslovaca_D-CWPP_CP003375.  GGGTCAATGCATTTATCGATGGTAGCATTAAAAGCTGATGGCACAAAGCC
sca4_RconJC480_MN581999.1       GGGTCAATGCATTTATCGATGGTAGCATTAAAAGCTGATGGCACAAAGCC
                                **************************************************

sca4_SL94_MZ970562              CTCCAAAGATAAAGCCGTATATTTCACTGCCCACTACGAAGAAGGACCAA
sca4_Rslovaca_D-CWPP_CP003375.  CTCCAAAGATAAAGCCGTATATTTCACTGCCCACTACGAAGAAGGACCAA
sca4_RconJC480_MN581999.1       CTCCAAAGATAAAGCCGTATATTTCACTGCCCACTACGAAGAAGGACCAA
                                **************************************************

sca4_SL94_MZ970562              ACGGTAAACCTCAACTTAAAGAAATAAG----------------------
sca4_Rslovaca_D-CWPP_CP003375.  ACGGTAAACCTCAACTTAAAGAAATAAGCTCACCAAAACCTTTAAAATTT
sca4_RconJC480_MN581999.1       ACGGTAAACCTCAACTTAAAGAAATAAGCTCACCAAAACCTTTAAAATTT
```

```
                                       **************************************************

sca4_SL94_MZ970562                     --------------------------------------------------
sca4_Rslovaca_D-CWPP_CP003375.         GCCGGAACCGGAGATGACGCAATAGCTTATATAGAGCATGGTGGAGAAAT
sca4_RconJC480_MN581999.1              GCCGGAACCGGAGATGACGCAATAGCTTATATAGAGCATGGTGGAGAAAT
                                       **************************************************

sca4_SL94_MZ970562                     --------------------------------------------------
sca4_Rslovaca_D-CWPP_CP003375.         TTATACACTTGCGGTAACACGCGGTAAATATAAAGAAATGATGAAGGAGG
sca4_RconJC480_MN581999.1              TTATACACTTGCGGTAACACGCGGTAAATATAAAGAAATGATGAAGGAGG
                                       **************************************************

sca4_SL94_MZ970562                     --------------------------------------------------
sca4_Rslovaca_D-CWPP_CP003375.         TAGAACTAAACCAAGGGCAGAGCGTTGATTTATCGCAAGCTGAAGATATT
sca4_RconJC480_MN581999.1              TAGAACTAAACCAAGGGCAGAGCGTTGATTTATCGCAAGCTGAAGATATT
                                       **************************************************

sca4_SL94_MZ970562                     --------------------------------------------------
sca4_Rslovaca_D-CWPP_CP003375.         ATAATAGGACAAGGACAAAGTAAGGAACAACCGCTAATAACTCCACAGCA
sca4_RconJC480_MN581999.1              ATAATAGGACAAGGACAAAGTAAGGAACAACCGCTAATAACTCCACAGCA
                                       **************************************************

sca4_SL94_MZ970562                     --------------------------------------------------
sca4_Rslovaca_D-CWPP_CP003375.         AACAACAAGTTCATCGGTTGAACCACCTCAGTATAAACAACAAGTACCGC
sca4_RconJC480_MN581999.1              AACAACAAGTTCATCGGTTGAACCACCTCAGTATAAACAACAAGTACCGC
                                       **************************************************

sca4_SL94_MZ970562                     --------------------------------------------------
sca4_Rslovaca_D-CWPP_CP003375.         CAATTACTCCTACTAACCAACCACTGCAAGCTGAGACTTCACAAATGCCA
sca4_RconJC480_MN581999.1              CAATTACTCCTACTAACCAACCACTGCAACCTGAGACTTCACAAATGCCA
                                       ***************************** ********************

sca4_SL94_MZ970562                     --------------------------------------------------
sca4_Rslovaca_D-CWPP_CP003375.         CAGTCGCAACAAGTGAATCCAAATCTTCTTAATGCAGCTACGGCCTTATC
sca4_RconJC480_MN581999.1              CAGTCGCAACAAGTGAATCCAAATCTTCTTAATGCAGCTACGGCTTTATC
                                       ******************************************** *****

sca4_SL94_MZ970562                     --------------------------------------------------
sca4_Rslovaca_D-CWPP_CP003375.         AGGCAGCATGCAAGATTTATTAAATTATGTAAATGCAGGTTTAACAAAAG
sca4_RconJC480_MN581999.1              AGGCAGCATGCAAGATTTATTAAATTATGTAAATGCAGGTTTAACAAAAG
                                       **************************************************

sca4_SL94_MZ970562                     --------------------------------------------------
sca4_Rslovaca_D-CWPP_CP003375.         AAATTGATAGCAATAAACAAATTGATTTAATTAAAGAAGCAGCCAAGGCA
sca4_RconJC480_MN581999.1              AAATTGATAGCAATAAACAAATTGATTTAATTAAAGAAGCAGCCACGGCA
                                       ********************************************* ****

sca4_SL94_MZ970562                     --------------------------------------------------
sca4_Rslovaca_D-CWPP_CP003375.         ATTCTTAATAATGAGAAAAGTGATATTGCTGAAAAGCAGGCTAATATCAT
sca4_RconJC480_MN581999.1              ATTCTTAATAATGAGAAAAGTGATATTGCTGAAAAGCAGGCTAATATCAT
                                       **************************************************

sca4_SL94_MZ970562                     --------------------------------------------------
sca4_Rslovaca_D-CWPP_CP003375.         TGCTTTAGCTGAAAATACGGTCAATAATAAAAACCTCAAACCGGATGCAA
sca4_RconJC480_MN581999.1              TGCTTTA CTGAAAATACGGTCAATAATAAAAACCTCAAACCGGATGCAA
                                       ******* ******************************************

sca4_SL94_MZ970562                     --------------------------------------------------
sca4_Rslovaca_D-CWPP_CP003375.         AAGTAGCTGGAGTCAATGCAGTATTAGAAACCATAAAAAATGATCAGAAT
sca4_RconJC480_MN581999.1              AAGTAGCTGGAGTCAATGCAGTATTAGAAACCATAAAAAATGATCAGAAT
                                       **************************************************

sca4_SL94_MZ970562                     --------------------------------------------------
```

```
sca4_Rslovaca_D-CWPP_CP003375.      ACCCCAGACCTAGAAAAATCAAAAATGCTTGAAGCTACAGTAGCTATCGT
sca4_RconJC480_MN581999.1           ACCCCAGCCCTAGAAAAATCAAAAATGCTTGAAGCTACAGTAGCTATCGT
                                    ******* ******************************************

sca4_SL94_MZ970562                  --------------------------------------------------
sca4_Rslovaca_D-CWPP_CP003375.      TTTAAATTCAGAGAATCTTGAGCCGAAGCAAAAACAGCAGATGTTAGAAA
sca4_RconJC480_MN581999.1           TTTAAATTCAGAGAATCTTGAGCCGAAGCAAAAACAGCAGATGTTAGAAA
                                    **************************************************

sca4_SL94_MZ970562                  --------------------------------------------------
sca4_Rslovaca_D-CWPP_CP003375.      AGGCAGTAGATGTCGGTTTAAGTCTTAAAGATGATGCAAGTAGAGCTGCA
sca4_RconJC480_MN581999.1           AGGCAGTAGATGTCGGTTTAAGTCTTAAAGATGATGCAAGTAGAACTGCA
                                    ******************************************** *****

sca4_SL94_MZ970562                  --------------------------------------------------
sca4_Rslovaca_D-CWPP_CP003375.      GCAATTGACGGTATTAAGGATGTTGTAATAAAAAGTAACCTTTCTACTGA
sca4_RconJC480_MN581999.1           GCAATTGACGGTATTAAGGATGTTGTAATAAAAAGTAACCTTTCTACTGA
                                    **************************************************

sca4_SL94_MZ970562                  --------------------------------------------------
sca4_Rslovaca_D-CWPP_CP003375.      AGATAAAGGGACAATGCTTATAGCAGTAGGTGATAAGGTTAATGTCTCTG
sca4_RconJC480_MN581999.1           AGATAAA---ACAATGCTTATAACAGTAGGTGATAAGGTTAATGTCTCTG
                                    *******   ************ ***************************

sca4_SL94_MZ970562                  --------------------------------------------------
sca4_Rslovaca_D-CWPP_CP003375.      AATTAAGCAATGCGGAAAAACAAAAATTATTAGGTTCTGTATTAAAGAAA
sca4_RconJC480_MN581999.1           AATTAAGCAATGCGGAAAAACAAAAATTATTAGGTTCTGTATTAAAG---
                                    ***********************************************

sca4_SL94_MZ970562                  --------------------------------------------------
sca4_Rslovaca_D-CWPP_CP003375.      AAAGGTGTAGAAGCCCAAGTTCTCAGTCCGGCACAACAACAATTGATGCA
sca4_RconJC480_MN581999.1           AAAGGTGTAGAAGCCCAAGTTCTCAGTCCGGCACAACAACAATTGATGCA
                                    **************************************************

sca4_SL94_MZ970562                  --------------------------------------------------
sca4_Rslovaca_D-CWPP_CP003375.      GCAGCATTTAGATAAGATTACGGCAGAACAAACTAAAAAAGATACAATAA
sca4_RconJC480_MN581999.1           GCAGCATTTAGATAAGATTATGGCAGAACAAATTAAAAAAGATACAATAA
                                    ******************** ************ ****************

sca4_SL94_MZ970562                  --------------------------------------------------
sca4_Rslovaca_D-CWPP_CP003375.      AAAAAGTAAATGATATTTTGTTTGATCCTCTAAGTAATACTGAATTAAAA
sca4_RconJC480_MN581999.1           AAAAAGTAAATGATATTTTGTTTGATCCTCTAAGTAATACTGAATTAAAA
                                    **************************************************

sca4_SL94_MZ970562                  --------------------------------------------------
sca4_Rslovaca_D-CWPP_CP003375.      ACAACAAACATACAAGCCATTACGTCTAATGTTTTAGATGGTCCGGCTAC
sca4_RconJC480_MN581999.1           ACAACAAACATACAAGCTATTACGTCTAATGTTTTAGATGGTCCGGCTAC
                                    ***************** ********************************

sca4_SL94_MZ970562                  --------------------------------------------------
sca4_Rslovaca_D-CWPP_CP003375.      AGCAGAAGTCAAAGGCGAAATAATTCAAGAAATTACTAACACAGTTGCAG
sca4_RconJC480_MN581999.1           AGCAGAAGTCAAAGGCAAAATAATTCAAGAAATTACTAACACAGTTGCAG
                                    **************** *********************************

sca4_SL94_MZ970562                  --------------------------------------------------
sca4_Rslovaca_D-CWPP_CP003375.      GAAGCTCGCTTGAAGCTCAAGACAAAGCAGCGATTATTAAAGGGGTAGGC
sca4_RconJC480_MN581999.1           GAAGCTCGCTTGAAGCTCAAGACAAAGCAGCGATTCTTAAAGGGGTAGGC
                                    *********************************** **************

sca4_SL94_MZ970562                  --------------------------------------------------
sca4_Rslovaca_D-CWPP_CP003375.      GAGACTATAGCTACTCATAGTGATACCTCTTTATCCTTACCTAATAAAGC
sca4_RconJC480_MN581999.1           GAGACTATAGCTACTCATAGTGATACCTCTTTATCCTTACCTAATAAAGC
                                    **************************************************
```

```
sca4_SL94_MZ970562                 --------------------------------------------------
sca4_Rslovaca_D-CWPP_CP003375.     ACTTATTATGGCATCAGCGGAAAAAGGTATTGCAGAAAGTCAGACCAATT
sca4_RconJC480_MN581999.1          ACTTATTATGGCATCAGCGGAAAAAGGTATTGCAGAAAGTCAGACCAATT
                                   **************************************************

sca4_SL94_MZ970562                 --------------------------------------------------
sca4_Rslovaca_D-CWPP_CP003375.     TACCTGATAGAGAGCTAATGACTAAAGGGTTAGTAGACGGTATTTATGAA
sca4_RconJC480_MN581999.1          TACCTGATAGAGAGCTAATGACTAAAGGGTTAGTAGACGGTATTTATGAA
                                   **************************************************

sca4_SL94_MZ970562                 --------------------------------------------------
sca4_Rslovaca_D-CWPP_CP003375.     GGCAAAGGAGGTCCTGAAATAACTAAGGCAGTTTCTAGCGGGATCGATAA
sca4_RconJC480_MN581999.1          GGCAAAGGAGGTCCTGAAATAACTAAGGCAGTTTCTAGCGGGATCGATAA
                                   **************************************************

sca4_SL94_MZ970562                 --------------------------------------------------
sca4_Rslovaca_D-CWPP_CP003375.     TAGTAATATTAATGACTCTGAGAAAGAGGCACTTAAAAAAGCTAAAGATG
sca4_RconJC480_MN581999.1          TAGTAATATTAATGACTCTGAGAAAGAGGCACTTAAAAAAGCTAAAGATG
                                   **************************************************

sca4_SL94_MZ970562                 --------------------------------------------------
sca4_Rslovaca_D-CWPP_CP003375.     CAGCGAGTGAGGCAGCTTTAGATAGAGACACTCAAAATTTAACTGAAGGG
sca4_RconJC480_MN581999.1          CAGCGAGTGAGGCCGCTTTAGATAGAGACACTCAAAATTTAACTGAAGGG
                                   ************* ************************************

sca4_SL94_MZ970562                 --------------------------------------------------
sca4_Rslovaca_D-CWPP_CP003375.     TTAAAAGGACAGAATATAGAAGAACACAAGCCTCACGATGATATATATAA
sca4_RconJC480_MN581999.1          TTAAAAGGACAGAATATAGAAGAACACAAGCCTCACGATGATATATATAA
                                   **************************************************

sca4_SL94_MZ970562                 --------------------------------------------------
sca4_Rslovaca_D-CWPP_CP003375.     CAAAGCTCGAGAAGTAATTAACGCTGTTAATCCTGTTATAGAAGCATTAG
sca4_RconJC480_MN581999.1          CCAAGCTCGAGAAGTAATTAACGCTGTTAATCCTGTTATAGAAGCATTAG
                                   * ************************************************

sca4_SL94_MZ970562                 --------------------------------------------------
sca4_Rslovaca_D-CWPP_CP003375.     AAAAATTTAAAGAACCGGTAGTGTCGGCAGAAGAAAGAATTGTACAAGAA
sca4_RconJC480_MN581999.1          AAAAATCTAAAGAACCGGTAGTGTCGGCGGAAGAAAGAACTGTACAAGAA
                                   ****** ********************* ********** **********

sca4_SL94_MZ970562                 --------------------------------------------------
sca4_Rslovaca_D-CWPP_CP003375.     ACTTCTAGTATATTAAATAATATCTCTAAGCTTGCAGTTGAGAAAGTCAA
sca4_RconJC480_MN581999.1          ACTTCTAGTATATTAAATAATATCTCTAAGCTTGCAGTTGAGAAAGTCAA
                                   ************************** ***********************

sca4_SL94_MZ970562                 --------------------------------------------------
sca4_Rslovaca_D-CWPP_CP003375.     TAATTTTCGTGCTATGCTTTCTCCAACTGGTAACCTTAAAACTCTTGAAG
sca4_RconJC480_MN581999.1          TAATTTTCGTGCTATGCTTTCTCCAAATGGTAACCTTAAAACTCTTGAAG
                                   ************************** ***********************

sca4_SL94_MZ970562                 --------------------------------------------------
sca4_Rslovaca_D-CWPP_CP003375.     AAAAAAAAGAGGAATCAATAAAAAAAGTAGATGAGCTGGTAAAGGCATTT
sca4_RconJC480_MN581999.1          AAAAAAAAGAGGAATCAATAAAAAAAGTAGATGAGCTGGTCAAGGCATTT
                                   **************************************** *********

sca4_SL94_MZ970562                 --------------------------------------------------
sca4_Rslovaca_D-CWPP_CP003375.     GGTACTAAATCTTCTACTGAAGAACAGCAAAGTTTCATTAAAACTAATTT
sca4_RconJC480_MN581999.1          GGTACTAAATCTTCTACTGAAGAACAGCAAAGTTTCATTAAAACTAATTT
                                   **************************************************

sca4_SL94_MZ970562                 --------------------------------------------------
sca4_Rslovaca_D-CWPP_CP003375.     AATTGATGATAAAACTTTATCTAAAGAGGTACGTTTACAAACTATAGATA
```

```
sca4_RconJC480_MN581999.1           AATTGATGATAAAACTTTATCTAAAGAGGTACGTTTACAAACTATAGATA
                                    **************************************************

sca4_SL94_MZ970562                  --------------------------------------------------
sca4_Rslovaca_D-CWPP_CP003375.      AGTTATTACAAGAACAAAAACGAGCAGAAGCAATTGAAAACCCTAGTGTT
sca4_RconJC480_MN581999.1           GGTTATTACAAGAACAAAAACGAGCAGAAGCAATTGAAAACCCTAGTGTT
                                     *************************************************

sca4_SL94_MZ970562                  --------------------------------------------------
sca4_Rslovaca_D-CWPP_CP003375.      AAAACGGAAGATGTAAGGGTAGTATCAGGAAAGTCTAAATTAAAACCTAT
sca4_RconJC480_MN581999.1           AAAACGGAAGATGTAAGGGTAGTATCAGGAAAGTCTAAATTAAAACCCAT
                                    *********************************************** **

sca4_SL94_MZ970562                  --------------------------------------------------
sca4_Rslovaca_D-CWPP_CP003375.      AAGTAAAGATAATCCAGATATTGAAAAAGCTAAAATGGTAGTAGGAAGAG
sca4_RconJC480_MN581999.1           AAGTAAAGATAATCCAGATATTGACAAAGCCAAAATGGTATTAGGAAGAG
                                    ************************ ***** ********* *********

sca4_SL94_MZ970562                  --------------------------------------------------
sca4_Rslovaca_D-CWPP_CP003375.      ATAGAGTTAATATCAAAGGGAATATAAAAATTATGGGAGCATTAATGAAT
sca4_RconJC480_MN581999.1           ATAGAGTTAATATCAAACGGAATATAAAAACTATGGGAGCATTAATG---
                                    ***************** ************ ****************

sca4_SL94_MZ970562                  --------------------------------------------------
sca4_Rslovaca_D-CWPP_CP003375.      GCAAGAGATATCATTCAGTCGGAAAATTTAAATAAATCAACACCTATTAA
sca4_RconJC480_MN581999.1           --------------------------------------------------


sca4_SL94_MZ970562                  ----------------------------
sca4_Rslovaca_D-CWPP_CP003375.      AAGAGAGTCTTCCCCTCCACAACGCTGA
sca4_RconJC480_MN581999.1           ----------------------------
```

**Supplemental Figure 2:** Comparisons of gene sequences of *Rickettsia* SL154 (part A) and *Rickettsia* SL94 (part B) and with the gene sequences of their respective near relatives.
